# Compilation of parasitic immunogenic proteins from 30 years of published research using machine learning and natural language processing

**DOI:** 10.1038/s41598-022-13790-1

**Published:** 2022-06-20

**Authors:** Stephen J. Goodswen, Paul J. Kennedy, John T. Ellis

**Affiliations:** 1 School of Life Sciences, University of Technology Sydney, 15 Broadway, Ultimo, NSW 2007 Australia; 2 School of Computer Science, Faculty of Engineering and Information Technology and the Australian Artificial Intelligence Institute, University of Technology Sydney, 15 Broadway, Ultimo, NSW 2007 Australia

**Keywords:** Computational biology and bioinformatics, Microbiology

## Abstract

The World Health Organisation reported in 2020 that six of the top 10 sources of death in low-income countries are parasites. Parasites are microorganisms in a relationship with a larger organism, the host. They acquire all benefits at the host’s expense. A disease develops if the parasitic infection disrupts normal functioning of the host. This disruption can range from mild to severe, including death. Humans and livestock continue to be challenged by established and emerging infectious disease threats. Vaccination is the most efficient tool for preventing current and future threats. Immunogenic proteins sourced from the disease-causing parasite are worthwhile vaccine components (subunits) due to reliable safety and manufacturing capacity. Publications with ‘subunit vaccine’ in their title have accumulated to thousands over the last three decades. However, there are possibly thousands more reporting immunogenicity results without mentioning ‘subunit’ and/or ‘vaccine’. The exact number is unclear given the non-standardised keywords in publications. The study aim is to identify parasite proteins that induce a protective response in an animal model as reported in the scientific literature within the last 30 years using machine learning and natural language processing. Source code to fulfil this aim and the vaccine candidate list obtained is made available.

## Introduction

Microorganisms exhibit diverse complex relationships with larger forms of life that harbour them^1^. These symbiotic relationships encompass a spectrum governed by the benefits or detriments experienced by the microorganism and host. At the extreme end of this spectrum is parasitism, in which the microorganism acquires all the benefits at the host’s expense^2^. Parasitism is thought to be the most common mode of life on this planet^3^. Parasitic relationships can vary between asymptomatic infections to one that kills the host^4^. Organisms known to display parasitic relationships by living on or in a host include bacteria, viruses, fungi, protozoa, and helminths. In this study, the term parasite refers only to protozoa, helminths and ectoparasites (parasites that exist on the external surface of hosts); and the hosts of interest are humans and livestock.

Humans and livestock have evolved over millennia in a constant balance between their immune-system defences and parasite virulence. Infection occurs when the balance shifts in favour of parasites as they multiply within or on the host’s body. A disease develops if the infection disrupts the normal functioning of the host. This disruption can range from mild to severe. Table 1 lists notable parasites of medical and veterinary importance and their associated infection/disease. Due to progressively improving methods for treatment and control of infectious diseases, human mortality and morbidity especially in the developing world has significantly declined over the last 50 years^5^. Despite the global decline, the World Health Organisation (WHO) reported in December 2020 that six of the top 10 causes of death in low-income countries are infectious diseases. These countries are mainly in tropical regions, with marginalized populations living in impoverished conditions^6^. Malaria tops the list of parasitic induced diseases that cause the greatest burden. WHO in 2019, estimated that there were 229 million cases of malaria worldwide with 409,000 deaths. The list of burdensome parasites and diseases is not static^7^. Rapid population growth in areas with weak health systems, urbanization, globalization, climate change, civil conflict, antimicrobial resistance, and the changing nature of pathogen transmission between human and animal populations^8^ entails that the world will continue to be challenged by established, unknown, neglected tropical diseases (NTDs), emerging, and re-emerging infectious disease threats. Infectious diseases also cost the livestock industries billions of dollars annually, aside from substantial animal suffering^9,10^.

**Table 1:** Important parasites and their associated infectious disease.

Parasite (*Genus*)^a^	Class^b^	Phylum	Disease^c^	Priority^d^
*Babesia*	Protozoa	Apicomplexa	Babesiosis	
*Cryptosporidium*	Protozoa	Apicomplexa	Cryptosporidiosis	F,W
*Cyclospora*	Protozoa	Apicomplexa	Cyclosporiasis	F
*Eimeria*	Protozoa	Apicomplexa	Coccidiosis	
*Neospora*	Protozoa	Apicomplexa	Neosporosis	
*Plasmodium*	Protozoa	Apicomplexa	Malaria	
*Sarcocystis*	Protozoa	Apicomplexa	Sarcocystosis	F
*Theileria*	Protozoa	Apicomplexa	Theileriosis	
*Toxoplasma*	Protozoa	Apicomplexa	Toxoplasmosis	F,W
*Trypanosoma*	Protozoa	Euglenozoa	Trypanosomiasis, dourine, surra	F,G,W
*Balantidium*	Protozoa	Ciliophora	Balantidiasis	F
*Ichthyophthirius*	Protozoa	Ciliophora	White spot	
*Entamoeba*	Protozoa	Evosea	Amebiasis	F,W
*Leishmania*	Protozoa	Euglenozoa	Leishmaniasis	G,W
*Dientamoeba*	Protozoa	Metamonada	Dientamoebaisis	
*Giardia*	Protozoa	Metamonada	Giardiasis	F,W
*Histomonas*	Protozoa	Metamonada	Histomoniasis	
*Trichomonas*	Protozoa	Metamonada	Trichomoniasis	
*Ancylostoma*	Helminthic	Nematoda	Ancylostomiasis, hookworm	G,W
*Angiostrongylus*	Helminthic	Nematoda	Angiostrongyliasis	
*Ascaris*	Helminthic	Nematoda	Ascariasis	F,G,W
*Baylisascaris*	Helminthic	Nematoda	Baylisascariasis	
*Cooperia*	Helminthic	Nematoda	Infection only	
*Dirofilaria*	Helminthic	Nematoda	Dirofilariasis/heartworm	
*Dracunculus*	Helminthic	Nematoda	dracunculiasis, guinea worm	G,W
*Enterobius*	Helminthic	Nematoda	Enterobiasis	
*Gnathostoma*	Helminthic	Nematoda	Gnathostomiasis	
*Haemonchus*	Helminthic	Nematoda	Haemonchosis	
*Loa*	Helminthic	Nematoda	Loiasis	
*Necator*	Helminthic	Nematoda	Necatoriasis, hookworm	G,W
*Onchocerca*	Helminthic	Nematoda	Onchocerciasis	G,W
*Pseudoterranova*	Helminthic	Nematoda	Anisakiasis	F
*Strongyloides*	Helminthic	Nematoda	Strongyloidiasis	
*Teladorsagia*	Helminthic	Nematoda	Teladorsagiosis	
*Toxocara*	Helminthic	Nematoda	Toxocariasis	
*Trichinella*	Helminthic	Nematoda	Trichinellosis	F,W
*Trichostrongylus*	Helminthic	Nematoda	Trichostrongylosis	
*Trichuris*	Helminthic	Nematoda	Trichuriasis	F,G,W
*Wuchereria*	Helminthic	Nematoda	Filariasis	G,W
*Clonorchis*	Helminthic	Platyhelminthes	Clonorchiasis	W
*Diphyllobothrium*	Helminthic	Platyhelminthes	Diphyllobothriasis	F
*Dipylidium*	Helminthic	Platyhelminthes	Infection only	
*Echinococcus*	Helminthic	Platyhelminthes	Echinococcosis	F,W
*Fasciola*	Helminthic	Platyhelminthes	Fascioliasis	F,W
*Fasciolopsis*	Helminthic	Platyhelminthes	Fasciolopsiasis	
*Hymenolepis*	Helminthic	Platyhelminthes	Hymenolepiasis	
*Moniezia*	Helminthic	Platyhelminthes	Infection only	
*Opisthorchis*	Helminthic	Platyhelminthes	Opisthorchiasis	F,W
*Paragonimus*	Helminthic	Platyhelminthes	Paragonimiasis	F,W
*Schistosoma*	Helminthic	Platyhelminthes	Schistosomiasis	G,W
*Taenia*	Helminthic	Platyhelminthes	Cysticercosis	F,W
*Haemaphysalis*	Ectoparasite	Arthropoda	Disease vector	
*Ixodes*	Ectoparasite	Arthropoda	Paralysis	
*Lucilia*	Ectoparasite	Arthropoda	Flystrike	

^a^Parasite = the genus of an organism that lives on or in a host organism and typically at the detriment of the host (genus is a taxonomic name defining a group of related living organisms made up of one or more species).

^b^Class = there are three main classes of parasites that can cause disease in humans and livestock: (1) protozoa (microscopic single-celled eukaryotes); (2) helminthic (multicellular organisms generally visible to the naked eye in their adult stages); and (3) ectoparasite (ticks, fleas, lice, and mites that attach or burrow into the skin).

^c^Disease = the name given to an abnormal condition detrimentally affecting the structure or function of all or part of a host organism due to parasite infection. ‘Infection only’ signifies multiplication of parasites occurs within or on a host’s body but does not disrupt the normal functioning of the host.

^d^Priority = F,G,W denotes priority diseases in need of a vaccine as determined by: (F) Food and Agriculture Organization of the United Nations (FAO)—Microbiological Risk Assessment series (https://www.who.int/publications/i/item/microbiological-risk-assessment-series); (G) Bill and Melinda Gates Foundation—Neglected Tropical Diseases (https://www.gatesfoundation.org/our-work/programs/global-health/neglected-tropical-diseases) and Uniting to Combat Neglected Tropical Diseases (https://unitingtocombatntds.org/ntds/); and (W) World Health Organisation (WHO) – Ending the neglect to attain the Sustainable Development Goals: A road map for neglected tropical diseases 2021–2030 (https://www.who.int/publications/i/item/9789240010352). URLs last viewed September 2021.

Vaccination is considered the most efficient tool for preventing current and future infectious disease threats^11^. The millions of lives saved due to vaccines against polio, smallpox, measles, diphtheria, tetanus, and rabies^12^ is testament to their effectiveness and future potential. An effective vaccine induces a pathogen-specific immune response providing long-lasting protection against infection^13^. The most effective type of vaccine so far has been live but attenuated whole-organisms with reduced virulence^11,14^. However, live attenuated vaccines have the potential to cause disease in immunosuppressed individuals; and/or are impracticable to grow in culture; and/or contain components likely to trigger detrimental side-effects, allergenic and/or reactogenic responses^15^; and/or present the possibility of reversion to a virulent form^16^.

Subunit vaccines contain only antigenic components sourced from the disease-causing organism^17^, such as specific proteins and/or polysaccharides. Although these non-living components on their own have generally proven to be less immunogenic than attenuated organisms, their safety superiority and easier manufacturing capacity without having to culture the pathogen^16^ makes them worthwhile endeavours for vaccine developers. Moreover, subunit components supported by appropriate vectors and adjuvants have the potential to enhance the efficacy^18^. Subunit vaccines can be further categorized into protein-based (e.g., acellular pertussis, hepatitis B, and human papillomavirus vaccines); polysaccharide (e.g., meningococcal vaccine); and conjugate (e.g. *Haemophilus influenza* type b vaccine)^7^. In this study, the subunit components of interest are proteins that possess immunogenic properties, which are expected to be proteins accessible to the immune system^19^.

The traditional approach to identifying subunit vaccine components involves first cultivating and dissecting the pathogen in the laboratory; followed by the identification of each isolated component. In 2000, reverse vaccinology was first proposed as a revolutionary idea to identify protein antigens in silico^20^. Previous studies detail the in silico vaccine discovery approach inspired by reverse vaccinology for parasites^21,22^, and for bacteria^23^. The following summarises this approach. Protein sequences, at the heart of the in silico approach, have been shown to encode signals that provide informative characteristics about the source protein, such as its destined subcellular location, the presence of transmembrane domains and epitopes, or whether it is anchored to a membrane. As to date however, there has been no detection of a sequence-derived characteristic of a parasitic protein indicating protective immunity in a host. Some predicted characteristics such as epitopes suggest immunogenicity, although direct methods of predicting epitopes recognised by T-cells and B-cells remain problematic^24,25^ (indirect prediction methods focusing on peptide binding to major histocompatibility complex (MHC) molecules have proved more computationally practicable but requires training data containing a sufficiently large set of MHC-peptide binding affinities that are experimentally validated and specific to the host of the target pathogen^26^). The in silico strategy, as a compromise, is based on the premise that immunogenic proteins will have a different profile of characteristics to non-immunogenic proteins, and immunogenic proteins are more likely to provide protective immunity. This profile difference is not distinguishable to an observer and requires a trained binary classifier implemented via supervised machine learning (ML). Training data are the initial information used to teach supervised ML algorithms in the process of developing a model, from which the model creates and refines its approaches required for classification. Consequently, quantity and quality of training data are paramount to the ML algorithm’s performance when given an unseen profile to classify. Ideal training data for the in silico strategy would comprise two labelled datasets of characteristic profiles: one set derived from proteins shown to induce a protective immune response in a host, and an opposing set derived from known non-immunogenic proteins. This ideal is currently not readily forthcoming for parasites and raises a fundamental cyclic conundrum that currently limits the in silico vaccine discovery potential. That is, a sufficient number of proteins verified to provide protective immunity are required in the training data to predict proteins likely to provide protection. The only known repository that contains protective antigens associated with parasites is a web database created in 2011 called Protegen^27^. It currently contains 167 unique parasitic antigens manually curated from selected peer-reviewed publications.

The PubMed database maintained by the National Center for Biotechnology Information (NCBI) at the U.S. National Library of Medicine (NLM) indicates that the first published investigation of a subunit vaccine^28^ was in 1966. Over the following decades an increasing trickle of publications has accumulated to 1112 with ‘subunit vaccine’ or ‘subunit vaccines’ in their title. It is difficult to ascertain from PubMed search terms and keywords, how many of these publications are specific to ‘protein-based’ subunit vaccines. Furthermore, it is estimated here that there are possibly hundreds of published studies over the past several decades without ‘subunit vaccine’ in their title, but still report immunogenicity results of parasitic proteins. The exact number is unclear, however, given the non-standardised keywords in publications. Despite this uncertainty in exact numbers, PubMed searches suggest there is a potential pool of immunogenic proteins that could fulfil the elusive training data requirement for the in silico vaccine discovery approach.

The aim of the current study is to ‘automatically’ identify all parasite proteins that induce a protective response in an animal model as reported in the scientific literature within the last 30 years. To achieve this aim, we have developed a computational pipeline that first classifies published abstracts using ML, and then extracts protein and/or gene names from the classified ‘abstracts of interest’ using natural language processing (NLP). Source code for the pipeline is provided via GitHub (a source code repository hosting service). The pipeline extracted 606 parasitic proteins from four phyla (Apicomplexa, Euglenozoa, Nematoda, and Platyhelminthes). All these proteins are reported in highly cited publications; and 485 of the 606 have evidence supporting their accessibility to the immune system. We judge them to have vaccine candidacy merit, and therefore relevant for ML training data and/or further investigation. Furthermore, protein characteristics of the candidates were extracted from existing resources or predicted from their sequences. A comparative analysis of these characteristics from different phyla is presented via tables and graphs. Unresolved limitations remain with the pipeline and in particular, it has a programmed inclination to identify more popular well-reported candidates to reduce the numbers of false positives. However, we believe this is the first reported attempt to ‘automatically’ generate a vaccine candidacy list from the scientific literature as a starting point for investigation, and is a superior time-saving alternative to a manual gathering process.

## Results

**Figure 1:**
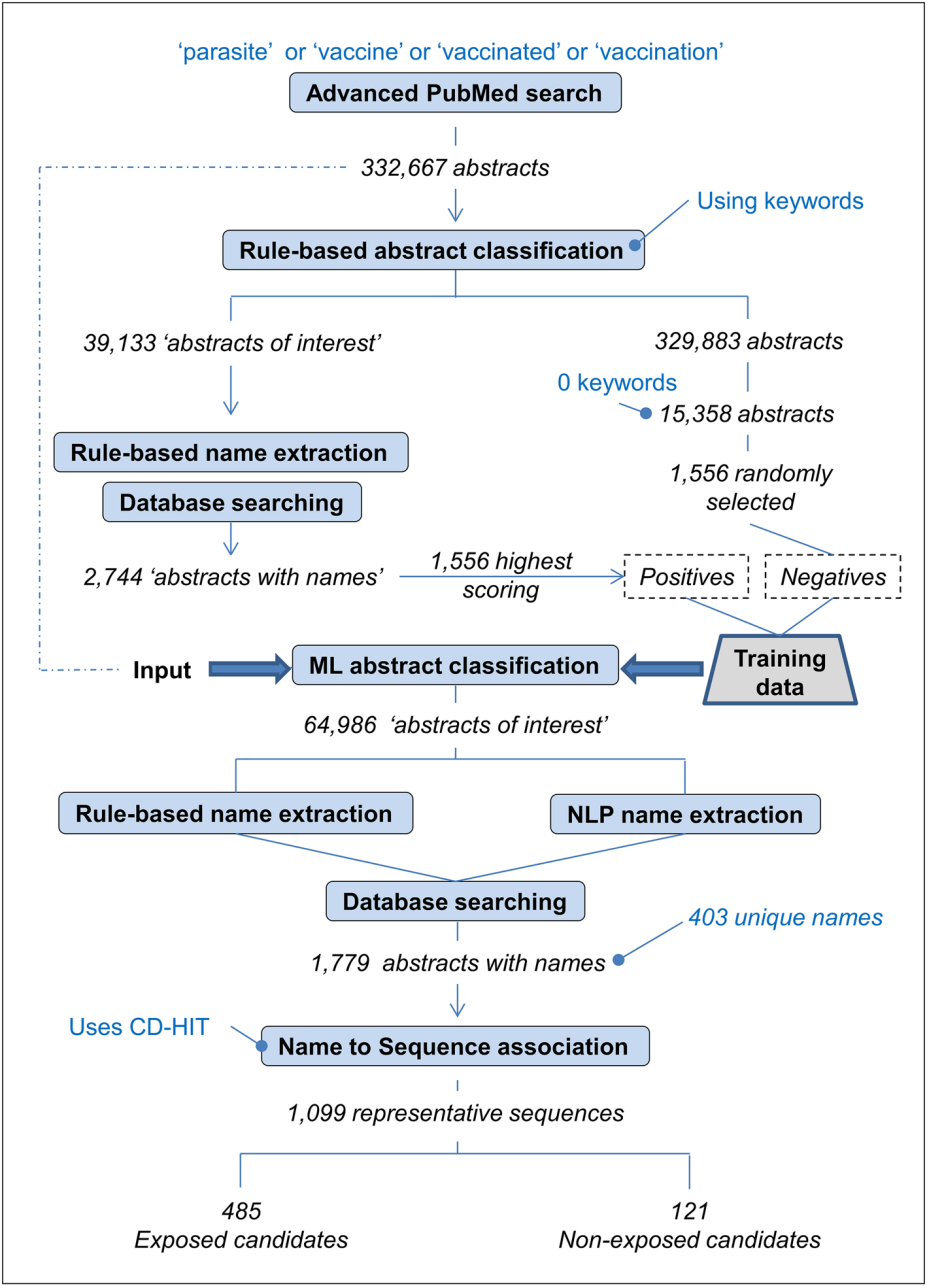
A schematic of the pipeline processes that takes abstracts as input and provides vaccine candidates as output. PubMed is a database maintained by the National Center for Biotechnology Information (NCBI) and contains over 30 million abstracts on life sciences and biomedical topics. The advanced search query was parasite, vaccine, vaccinated, OR vaccination in Title or Abstract text AND publication year greater or equal to 1991 and less than 2022. **Keywords** for the rule-based abstract classification were related to protective immunity, animal models, parasite species, and parasitic diseases. Note keywords were searched and counted in both title *and* abstract. The term ‘**abstract of interes**t’ refers to abstracts that potentially contain a protein name of a vaccine candidate. **Database searching** involves checking for a match of an extracted protein name in an in-house protein and gene database compiled from The Universal Protein Resource (UniProt) and NCBI. **Training data** consisted of abstracts converted to a vectorised format (i.e., a numerical representation) using the text vectorization technique, Bag of Words (BoW). **NLP** is an acronym for natural language processing. Named entity recognition (NER) is a sub-task of NLP and was used to classify named entities in abstracts into a pre-defined category of protein name. **CD-HIT** (cluster database at high identity with tolerance) was used to cluster 3731 sequences associated with 403 unique protein names into 1099 clusters, in which each member had a sequence similarity identity greater than 90%. A **representative sequence** is the longest sequence in a cluster. **Exposed candidates** are proteins naturally exposed to the immune system, whereas **non-exposed** are normally located in the pathogen’s interior.

Figure 1 shows a schematic of the entire pipeline that is designed to take abstracts as input and provide as output, a list of vaccine candidates. The pipeline consists of different stages and the presented results are in accordance to the stage’s approach used to obtain them: (1) rule-based abstract classification; (2) ML abstract classification, (3) rule-based and NLP protein name extraction, and (4) protein name to sequence association.

### Classification of abstracts using a rule-based approach

All publications over the last 30 years that contained either the word ‘parasite’, ‘vaccine’, ‘vaccinated’, or ‘vaccination’ in its title or abstract text were downloaded from PubMed—332,627 publications met this selection criteria. Each title and associated abstract was assessed to determine whether it contained matching words in keyword files (see Material and methods—Abstract classification using a rule-based approach). For instance, an abstract was classified as one of importance (a positive) if it contained defining words for the following: a parasite species, protective immunity, an animal model, a parasitic disease, and a gene or protein name associated with parasites. Supplementary Table S1 (an Excel file, sheet [Rule_based]) lists 2744 PubMed IDs and their relative keyword counts that were classified as positives e.g., the ‘title+abstract’ for PubMed ID ‘31815006’ contains 16 ‘protective immunity’ keywords, one animal model, one parasite species, one parasitic disease, and one protein name (surface antigen protein). Sheet [Identified_protein_names] lists 1752 unique protein names identified within these abstracts along with the number of publications containing the name. Abstracts with the greatest number of protective immunity keywords are considered here as the most likely to contain a vaccine candidate. Similarly, protein names associated with a greater number of publications are considered more likely vaccine candidates than those with fewer publications. For example, ‘Circumsporozoite protein’ is mentioned in 319 publications, whereas ‘1-cys peroxiredoxin’ only one (718 of the 1752 unique protein names have only one publication).

Two sets of 100 abstracts were randomly selected from the 2744 classified abstracts. One set contained only abstracts with more than one protective immunity keyword (2308 met this criteria); whilst the other set contained only one keyword, excluding those with ‘vaccine’ or ‘vaccination’ (381 met this criteria). These abstracts were manually verified. Supplementary Table S1 (sheets [Verified abstracts > 1] and [Verified abstracts = 1]) shows PubMed IDs and their keyword counts for the two sets. An additional column indicates a manually assessed ‘yes’ or ‘no’ as to whether the abstract is truly one of interest *and* has a relevant protein name. This manual assessment suggests the rule-based approach has a 42.5% accuracy of selecting an ‘abstract of interest' but increases to 85% when the classifying threshold is greater than one protective immunity keyword. This accuracy, however, reduces to 80% in its capacity to fully identify the relevant protein name. These manually assessed abstracts are referred to henceforth as the ‘Verified positive’ and ‘Verified negative’ abstracts, and used later to evaluate the ML algorithm’s performance.

As a further independent test, the title and abstract were taken from 50 publications known to contain vaccine candidates (see Material and methods–Evidence abstracts). The rule-based criteria were applied to the 50 ‘title+abstracts’ (referred to henceforth as the ‘Evidence’ abstracts). The results are shown in Supplementary Table S1—sheet [Rule_based_evidence]. Blank cells or text in bold indicates that no keyword was found for specific selection criteria e.g., the ‘title+abstract’ for PubMed ID ‘24349483’ does not contain an animal model. Using the stringent rule-based selection criteria, only 58% would be classified abstracts of interest. This is because most of the abstracts contain no ‘disease’ keyword. Ignoring the disease criterion, the relevant protein name is correctly identified in 82% of the abstracts.

### Classification of abstracts using machine learning

**Figure 2:**
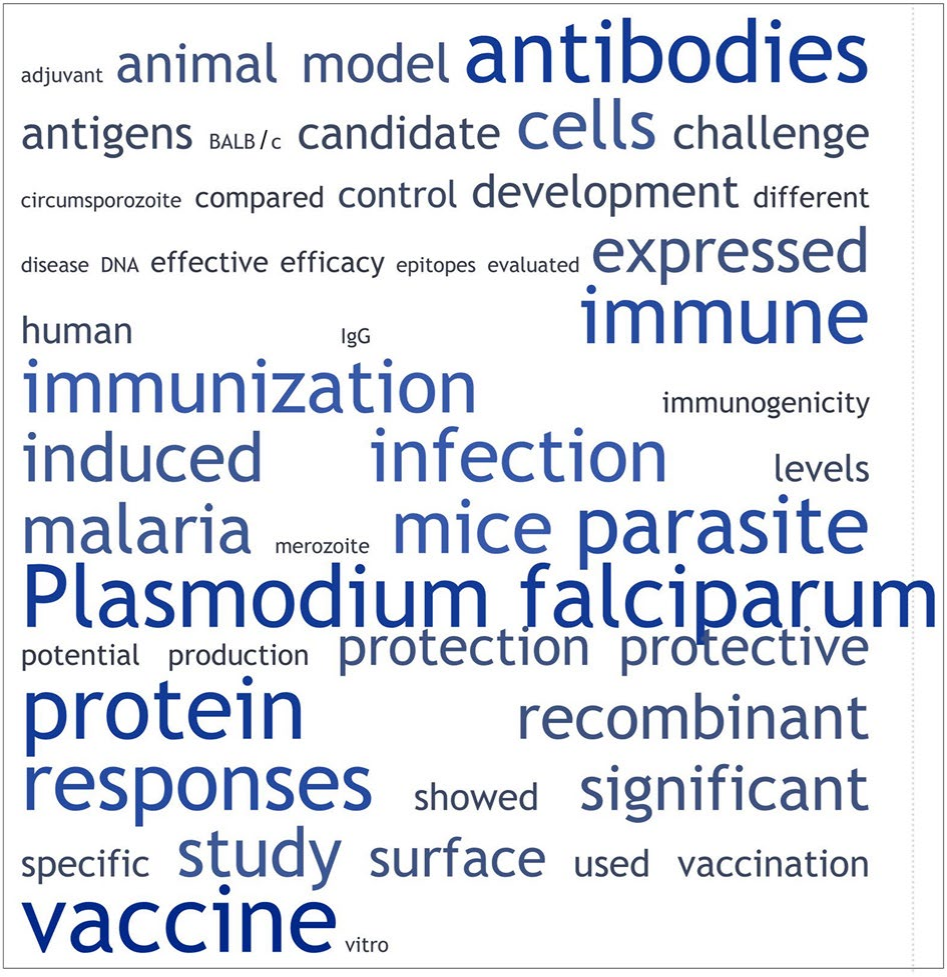
A word cloud showing the 50 most frequent words in the positives training data applied in the classification of abstracts using machine learning. Note that stop words e.g., “a”, “the”, “is”, “are” etc. were removed and a standard Porter Stemming algorithm applied to detect and combine similar words e.g., words such as responses and response or significant and significantly are combined (the most frequent of the variants is chosen to represent them). TagCrowd (https://tagcrowd.com/) was used to generate the word cloud.

An in-house ML pipeline was created that classifies an unseen abstract as either one of interest i.e., one potentially containing a vaccine candidate (a positive) or one that is not of interest (a negative). Materials and methods—Abstract classification using machine learning—describes the pipeline. The pipeline accuracy as determined from tenfold cross-validation of the ML training data (1556 positives and 1556 negatives) was 99.6% (see Supplementary Table S2 [SVM performance measures] for other measures used to evaluate the pipeline’s predictive performance). This high accuracy comes with a caveat. The positive or negative categorisation of training data abstracts was determined by the rule-based approach. Given the previous verification results, the expectation is that an unknown 15% of this categorisation is potentially incorrect. Figure 2 shows a word cloud of the 50 most frequent words in the positives training data. Supplementary Table S2 (sheets [Positives] and [Negatives]) shows the cross-validation-derived probability for each training abstract that it has been correctly classified e.g., 4 out of 1556 positives have a less than 50% probability that the classification is correct.

The in-house ML pipeline was applied to the Evidence and Verified abstracts, which are independent of the training abstracts. Supplementary Table S2 shows the results. Only one Evidence abstract had a probability less than 0.5, and therefore 49/50 (98%) were correctly classified as abstracts of interest—see sheet [Evidence abstracts]. The results from the Verified abstracts indicate the ML pipeline accuracy in correctly classifying an abstract of interest is 83% with a sensitivity and specificity of 98.8% and 71.3%, respectively. This means the classifier correctly predicts a positive more often than a negative e.g., with respect to the Verified abstracts, one ‘abstract of interest’ would be incorrectly rejected and 33 abstracts that are not of interest would be incorrectly accepted for the next stage of protein/gene name extraction—see sheets [Verified positive abstracts] and [Verified negative abstracts].

**Figure 3:**
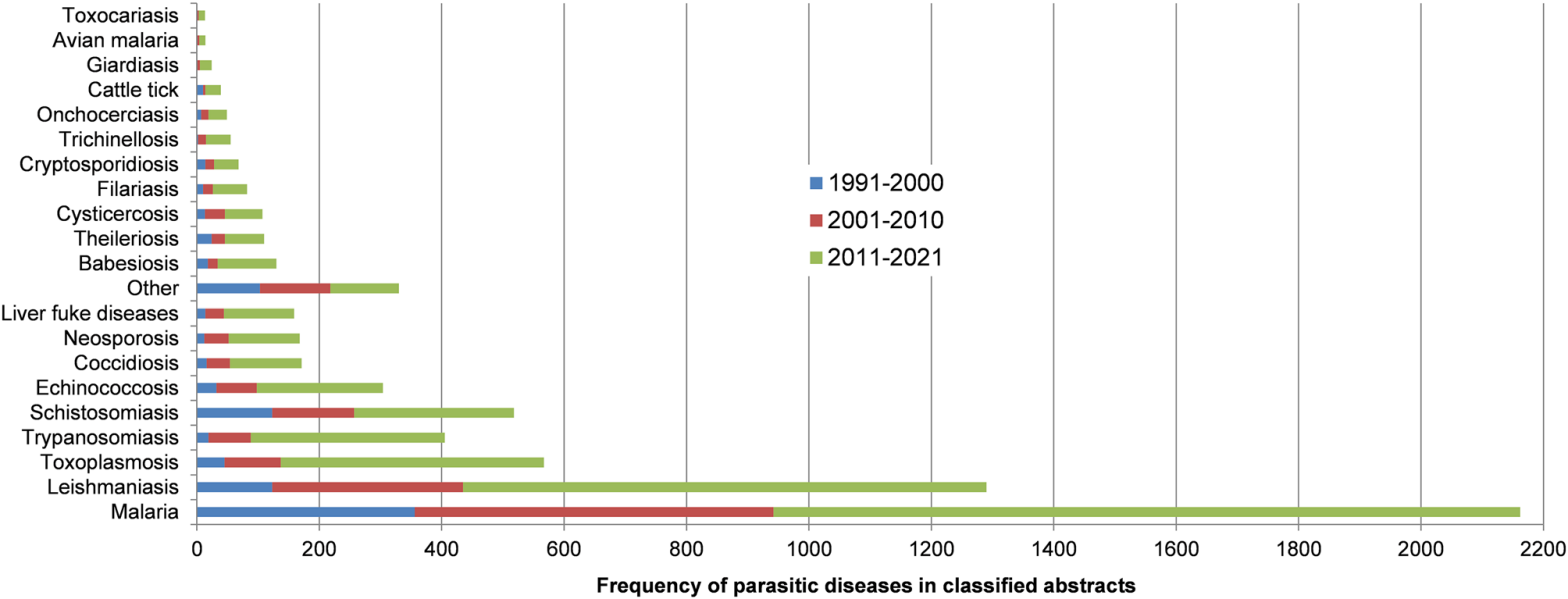
A bar chart showing frequency of disease words in classified abstracts over three decades from 1991 to 2021. The classified abstracts are ‘title+abstract’ text output from the machine learning abstract classification stage of the current study i.e., given an initial input of 332,627 ‘title+abstract’ texts downloaded from PubMed, 64,986 had a classification probability greater than or equal to 50% and were deemed ‘abstracts of interest’ (e.g.; an abstract that potentially contains a protein name of a vaccine candidate). Each word or a series of words associated with a parasitic disease were counted in the abstracts of interest e.g., the word ‘malaria’ appears 2162 times and ‘toxocariasis’ 13 times in the abstracts. The bar chart shows that each decade has a greater disease frequency than the decade before; and the frequency has more than doubled in the last 10 years (except for schistosomiasis and cysticercosis). Note that for brevity, counts of words related to the same or similar diseases were combined e.g., the diseases Chagas disease, American trypanosomiasis, African trypanosomiasis, and sleeping sickness are all caused by trypanosomes. The word counts associated with these diseases were combined and presented under trypanosomiasis.

**Figure 4:**
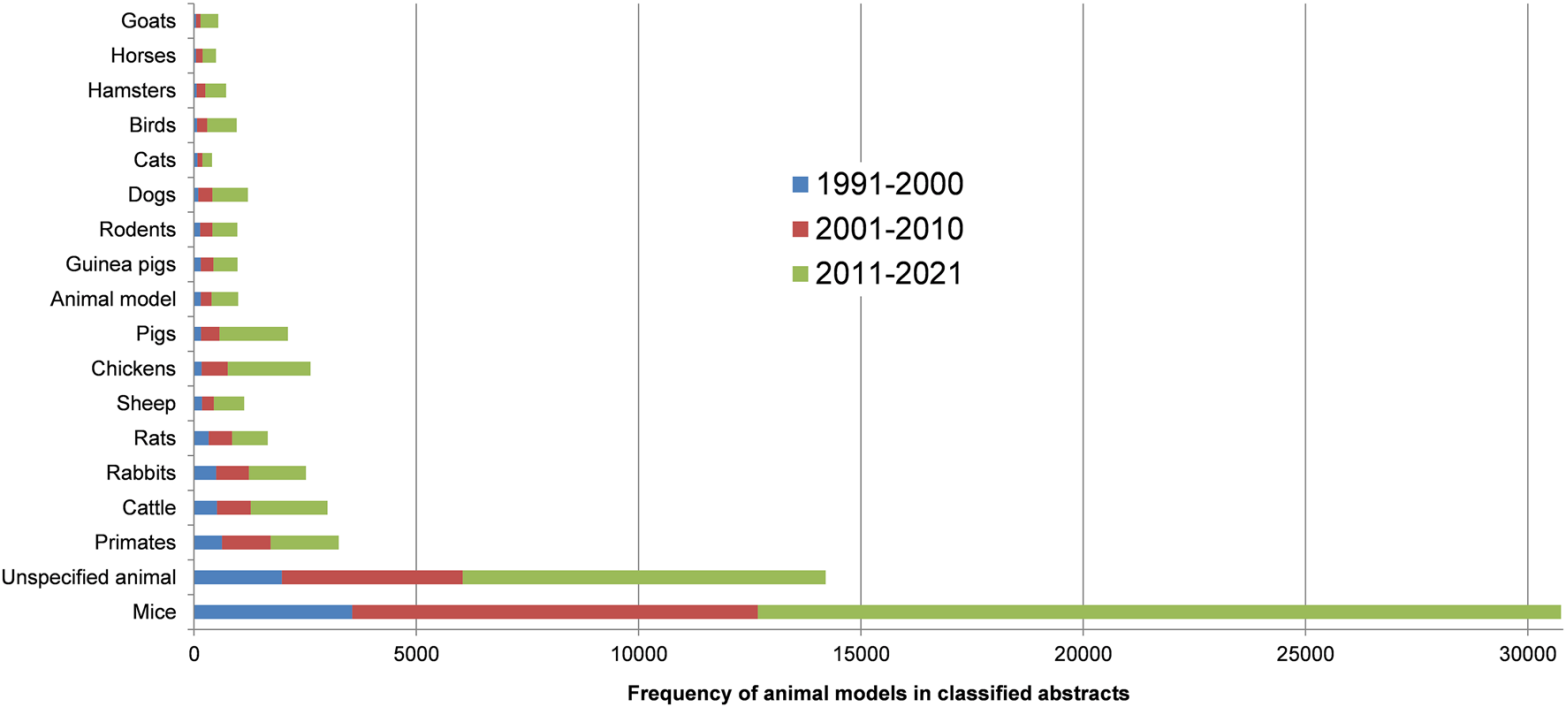
A bar chart showing frequency of ‘animal model’ words in classified abstracts over three decades from 1991 to 2021. The classified abstracts are ‘title+abstract’ text output from the machine learning abstract classification stage of the current study i.e., given an initial input of 332,627 ‘title+abstract’ texts downloaded from PubMed, 64,986 had a classification probability greater than or equal to 50% and were deemed ‘abstracts of interest’ (e.g.; an abstract that potentially contains a protein name of a vaccine candidate). Each word or a series of words describing an animal were counted in the abstracts of interest e.g., the word ‘mice’ appears 30,749 times and ‘goats’ 545 times in the abstracts. Note that the automated approach does not distinguish whether the animal words relate to a model for candidate verification or reference to another context such as an animal host. The bar chart shows that each decade has a greater frequency for each ‘animal model’ word than the decade before. The rate of increase in frequency has doubled in the last 10 years for the following (listed in descending rates): pigs, chickens, cattle, birds, goats, dogs, and sheep. Conversely, the rate of increase has slowed for the following (listed in ascending rates): primates, rats, rabbits, mice, and guinea pigs. Note that for brevity, counts of words related to the same or similar animal model were combined e.g., the ‘cattle’ animal model comprises word counts for cow, cows, calf, calves, and cattle.

The ‘title+abstract’ from all 332,627 downloaded publications were input into the ML pipeline. Approximately 22% of these input abstracts had an equal or greater than 50% probability of being correctly classified as an ‘abstract of interest’—16.8% with probability greater than 75%, and 12.5% greater than 90% (see sheet [All abstracts >  = 0.5]). Abstracts of interest have steadily increased from 420 publications in 1991 to 4619 in 2020 (except years 2002 and 2016 showed declines from the year before). Supplementary Data S1 displays a graph of these publication numbers. Figures 3 and 4 show the frequency of words related to animal models, parasitic species, and parasitic diseases within classified abstracts over the last three decades.

### Protein name extraction using rules and natural language processing

SpaCy is an open-source library for advanced NLP in Python (https://spacy.io/—last viewed September 2021). It provides named entity recognition (NER) functionality as one of its options. NER, a sub-task of information extraction, finds and classifies named entities in text into pre-defined categories such as names of persons, organizations, and countries. In this study, our entities of interest are protein and gene names. We investigated three types of NER trained models: SpaCy default models (https://spacy.io/models), scispaCy, and a SpaCy custom model trained for this study (see later ‘Protein name detection using natural language processing’). scispaCy is a Python package containing spaCy models trained for processing biomedical, scientific or clinical text^29^. As an illustration, Supplementary Data S2 contains two example abstracts that were processed with various spaCy, scispaCy, and custom NER models, with the aim to identify protein and genes, either via a symbol or name e.g., identify EG95 in Example #1, and TgPI-1, ROP2, GRA4, and serine protease inhibitor-1 in Example #2 (expected entities are highlighted in bold). Following the examples are the identified entities. All expected entities were identified by the evaluated scispaCy NER models. However, from this study’s perspective, there were numerous false positive entities related to scientific terminology, which was not a surprise considering scispaCy is designed to identify biomedical and scientific entities. The custom NER model, trained specifically to extract gene and protein name entities, successfully identified the expected names with only one false positive.

The Evidence and the Verified abstracts were processed with the custom NER model and a rule-based approach (see later ‘Protein name extraction using a rule-based approach’). An important point is that entities identified by both approaches are checked for a match in an in-house compiled protein and gene database (see Materials and methods). The accuracies in identifying ‘database verified’ protein or gene names were 88% and 91% (custom NER model), and 95% and 97% (rule-based) given Evidence and Verified abstracts, respectively. The rule-based accuracy is higher entirely because of the database verification e.g., a greater percentage of the entities checked for database verification are not valid protein names, whereas a substantially smaller percentage are invalid for the custom NER model. This equates to the rule-based approach generating more false positives and less false negatives; and conversely, the custom NER model generating less false positives and more false negatives. Using a consensus of the ‘rule-based+custom NER model’ entities, the accuracies reduce to 86% and 90% given Evidence and Verified abstracts, respectively.

### Combined abstract classification and subsequent protein extraction

Supplementary Table S3 (sheet [Candidates per PubID]) lists 1776 PubMed IDs and their relative keyword counts that were classified as abstracts containing protein names considered worthy vaccine candidates for further investigation. This list was achieved by first performing a ML classification of ‘title+abstract’ from 332,627 publications to obtain ‘abstracts of interest’, and then a protein name extraction using a consensus of the ‘rule-based+custom NER model’ approaches. An important point is that different selection criteria and thresholds can be applied that greatly dictate the number and quality of proteins in the output list. Namely: start and end year for publications; a threshold applied to ‘abstract of interest’ probability i.e., the output probability from the ML abstract classification; a threshold applied to the number of publications containing a candidate protein; and a threshold applied to the number of animal models referenced in the ‘abstract of interest’. Supplementary Data S3 demonstrates the impact of different selection criteria and thresholds. The main impact is that the more stringent the selection criteria, the more false negatives and potentially less false positives. The criteria used to obtain the 1776 proteins were Probability threshold >  = 0.99, Publication threshold >  = 3, Animal model threshold >  = 1, Year Start >  = 1991; Year End <  = 2021 (Discussion expands on the rationale for the thresholds chosen).

The protein names extracted from the 1776 classified abstracts were compiled into one list of 403 unique names (see Supplementary Table S3—sheet [Candidates]). This list also includes the number of publications that mention the unique ‘protein name’ given the 332,627 abstracts. Note that the uniqueness of the name is with reference to the usage in the abstracts e.g., Apcial membrane antigen I, Apical membrane antigen, apical membrane antigen 1, Apical membrane antigen 1, and Apical membrane antigen-1 are the exact names extracted from ‘abstracts of interest’. It is likely these names all represent the same protein, however, they are valid names with unique records in The Universal Protein Resource **(**UniProt) database^30^ e.g., the assumed misspelt ‘Apcial membrane antigen I’ has the UniProt ID Q26162. Several names were incorrectly extracted from abstracts due to the following two reasons: (1) an incorrect link of a ‘gene name from an abstract’ to a ‘protein in a database’ e.g., some abstracts contain the words ‘circumsporozoite (CS) protein’. CS is the gene name for both Chorismate synthase and Citrate synthase. Protein names extracted from such abstracts included Circumsporozoite protein, Chorismate synthase, and Citrate synthase; (2) an incorrect name extraction from a larger name e.g., for abstracts containing ‘Calcium-dependent protein kinase’, ‘Apical membrane antigen 1’, and ‘heat shock protein 70’, the names ‘protein kinase’, ‘Apical membrane antigen’, and ‘heat shock protein’ were also extracted because they are valid names associated with unique Uniprot IDs.

### Protein name to sequence association

The study aim was to not only obtain a list of vaccine candidate names but to associate the name to a relevant protein sequence. Two challenges had to be overcome to fulfil this aim. First, the inconsistency in protein names as previously highlighted with the ‘Apical membrane antigen’ example; and second, the name association with more than one sequence e.g., ‘Circumsporozoite protein’ is one of the 403 protein names extracted. In the UniProtKB database (release 2021_03), there are 3281 (unreviewed i.e., computationally analysed records) and 26 (reviewed i.e., manually annotated records) proteins with the name ‘Circumsporozoite protein’. Given only abstracts, it was not possible to determine which of these proteins was used in the related study’s vaccine candidacy evaluation. We did, nonetheless, narrow down the number of protein possibilities by using only proteins from the species specified in the abstract e.g., *Plasmodium falciparum* has 1098 proteins named ‘Circumsporozoite protein’. Despite using only species-related protein names, 29,648 sequences could be associated with the 403 protein names.

The following approach was implemented to overcome the two challenges. First, all ‘partially’ sequenced proteins (i.e., proteins annotated as being a fragment) were removed leaving 3731 sequences. Second, a CD-HIT (cluster database at high identity with tolerance)^31^ analysis was performed on the 3731 sequences. CD-HIT provides functionality to cluster protein sequences that meet a sequence similarity identity threshold. The threshold chosen here was 90%. The CD-HIT analysis created 1099 clusters (see Supplementary Table S4—sheet [Clusters]). As an example, cluster #19 has 73 sequences all with the same name ‘Transmission-blocking target antigen P230’. These sequences formed one cluster because they meet a sequence similarity identity greater than 90%. The longest sequence from each cluster was chosen as the cluster’s ‘representative’ e.g., UniProt ID H1AAD5_PLAVI is the representative sequence for cluster #19. All representative sequences are denoted by a ‘*’ in the Identity column. Note that the identity threshold is another variable that can greatly impact results.

**Figure 5:**
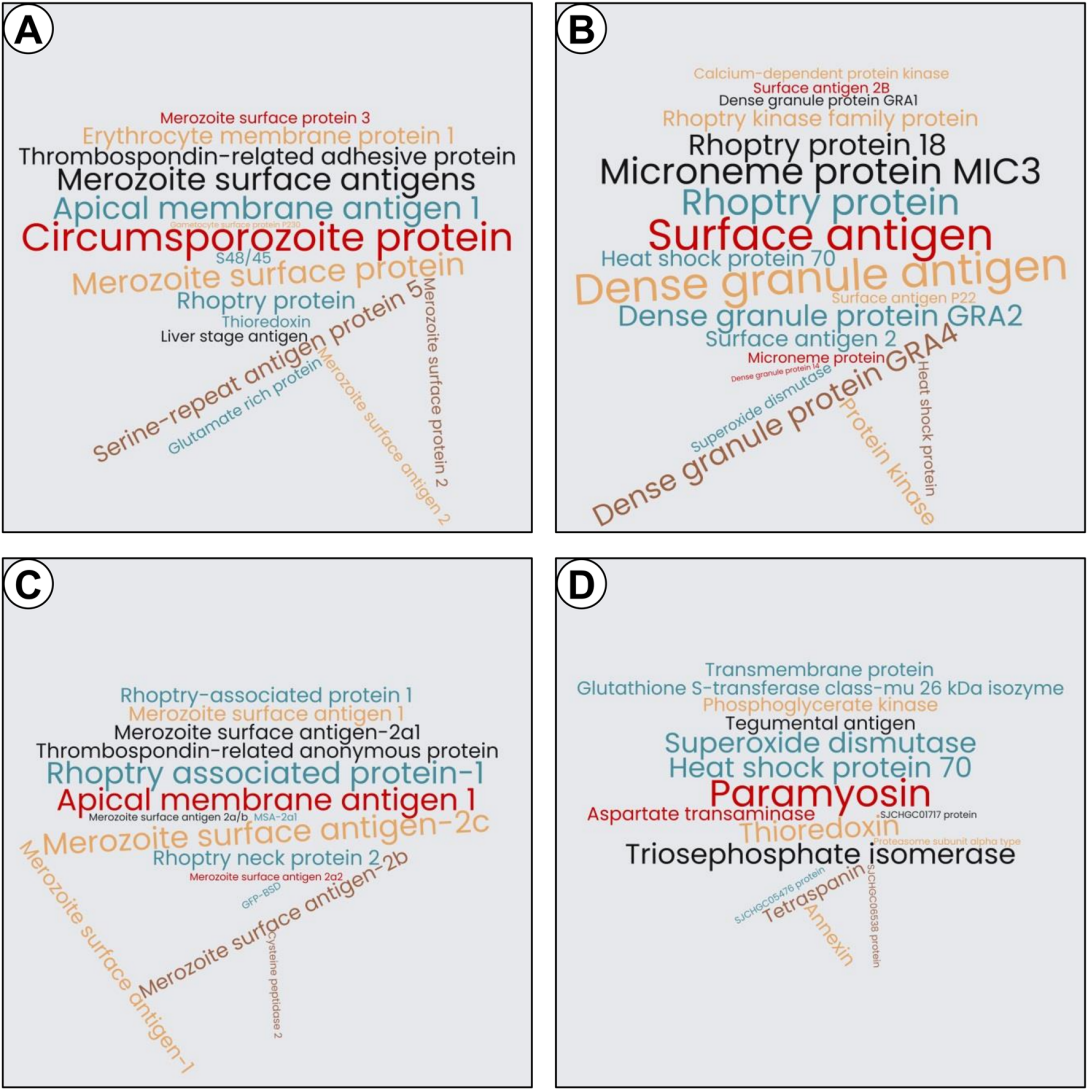
A word cloud showing the 15 most reported protein names per organism in the last 30 years of published research for four important parasite species. The size of the name is proportional to the number of publications reporting the protein. These protein names were ‘automatically’ extracted by the current study’s computational pipeline, which is designed to identify, from publication abstracts, parasite proteins that induce a protective response in an animal model. The presented names are from the top four species based on the total number of proteins identified: (**A**) *Plasmodium Falciparum*, (**B**) *Toxoplasma gondii*, (**C**) *Babesia bovis,* and (**D**) *Schistosoma japonicum*. Wordclouds.com (https://classic.wordclouds.com/) was used to generate the word cloud.

Supplementary Table S4 (sheet [Representative Names]) shows the representative names for the 1099 clusters. The representative name chosen is the majority name in a cluster. This resolved the problem of having the same protein but with slight variations in the annotation e.g., the 47 members in cluster #724 have three variations: one ‘Merozoite surface antigen 2’, 18 ‘Merozoite surface antigen 2C’, and 28 ‘Merozoite surface antigen-2c’. Sheet [Unique Names] lists 438 unique names from the 1099 representative names. Note that some names are similar e.g., Hsp70 and HSP70. Despite the name similarity, the associated sequences have a sequence similarity identity less than 90%, and in most cases associated with different species e.g., Hsp70 from *Leishmania infantum* and *Leishmania donovani*, and HSP70 from *Schistosoma japonicum*. Figure 5 is a graphical representation proportional to the frequency of the unique names given publications over the last 30 years for four important parasitic species. Supplementary Table S4 (sheet [Unique Names per Species]) shows for every parasitic species extracted from the abstracts, their unique names along with the number of source publications.

The lists of ‘representative names’ and ‘unique names’ in Supplementary Table S4 also include the following metrics per protein: number of publications containing the name, number of times each publication has been cited, an h-index and g-index of the names’ source publications (see later ‘Calculating h-index and g-index’), first year the name was reported in a publication (over the last 30 years), most recent year the name was reported, time period in years covering the number of publications and citations, and the number of years since last reported. Collectively, these metrics give a guideline as to the importance of each protein in the list. For example, ‘Circumsporozoite protein’ is without doubt an important protein worthy of inclusion in the list. Conversely, the one publication containing ‘Calpain’ has not been cited since 1996, and although the one publication containing ‘Bifunctional dihydrofolate reductase-thymidylate synthase’ has been cited 141 times, the protein has not been reported in any other ‘abstract of interest’ since 1998. Questionable proteins were flagged where the h-index was less than or equal to one *and* if the publication was more than 5 years old—114 of the 438 unique proteins were questionable with respect to vaccine candidacy. We therefore propose that 324 proteins are worthy candidates for further investigation.

To provide a guide to the reliability of the proposed candidates, a random selection of publications associated with the candidates were manually read. Table 2 shows a sample of publications that were automatically classified in this study to contain parasite proteins that induce a protective response in an animal model. A manual verification confirmed that nine out of 10 were correctly classified (e.g., calcium-dependent protein kinase 1 is not considered a potential vaccine candidate), although only six publications support the vaccine potential of the associated protein. Similarly, Table 2 shows a sample of proposed candidates with their mapped UniProt IDs associated with a publication. A manual verification confirmed that the vaccine potential of seven candidates out of 10 is supported by publications. Tables 2 and 3 also highlight some of the challenges restricting the reliability. First example, the protein name in a publication was ‘merozoite surface protein 1’, but the study’s automated pipeline incorrectly used ‘merozoite surface protein’ as the name to link to a Uniprot ID. Second example, the protein ‘Citrate synthase’ is not a valid candidate, despite the h-index. The automated pipeline incorrectly used this protein name instead of ‘Circumsporozoite protein’. This is because many publications have used the term ‘circumsporozoite (CS)’ antigen or protein and CS is one of the gene names for ‘Citrate synthase’.

**Table 2:** Manual verification of proteins linked to positively classified publications.

PubMed ID	Year	Row	Protein(s)	UniProt ID	e_Protein(s)	e_UniProt	Species	Prot
28424680	2017	2	Nucleoside triphosphate hydrolase-II^a^	A0A7J6JVX4	Hydrolase, NTPase II	A0A7J6K3V0 A0A7J6JVX4	*T. gondii*	Yes
23928460	2013	11	Rhoptry protein 5 (ROP5)	Q3YJR4	Rhoptry protein ROP5, Type II rhoptry protein 5A, Type III rhoptry protein 5A, Type I rhoptry protein 5A	A0A125YQ30 *Q3YJR4* B9QLN8 B9Q3F2	*T. gondii*	Yes
26421596	2014	22	*Toxoplasma gondii* 10 kDa excretory–secretory antigen (TgESA10)	R4H6A8	Ubiquitin	*R4H6A8*	*T. gondii*	Yes
14500491	2003	33	Merozoite surface protein 1 (MSP1)^a^	P13828	Merozoite surface protein 1	Q9GSA3	*P. yoelii*	Yes
29599776	2018	44	Amastigote 2 (A2)^a^	A4HZU7 Q26351	Stage-specific S antigen-like protein, stage-specific S antigen homolog,	A4HZU7 Q26351	*L. infantum*	Yes
7595214	1995	1737	Sporozoite surface protein 2 (PfSSP2)^a^	Q26020	Sporozoite surface protein 2	Q26020	*P. falciparum*	No^b^
21715579	2011	1747	Pfs230^a^	P68874	Gametocyte surface protein P230	P68874	*P. falciparum*	Yes
9106193	1996	1757	Merozoite surface protein 1^a^	P04933	Merozoite surface protein 1	Q8IJ53	*P. falciparum*	No^b^
29524527	2017	1767		False positive			*T. gondii*	No
11349025	2001	1777	Paramyosin (Pmy)^a^	A0A3Q0KD88	Paramyosin	A0A3Q0KD88	*S. mansoni*	No^b^

Year = year of publication; Row = row position on sheet [Candidates per PubID] in Supplementary Table S3. This sheet contains 1776 PubMed IDs and their relative keyword counts that were classified as abstracts containing protein names considered worthy vaccine candidates for further investigation. The rows are in descending order based on the ‘Protection’ column. Rows were chosen at regular intervals from the top and bottom for manual verification; Protein(s) = protein name(s) specified in publication. Names with strikethrough are not vaccine candidates and therefore the classification is a false positive; UniProt ID. = UniProt ID(s) linked to protein name; e_Protein(s) = names automatically extracted from publication title+abstract; e_UniProt = UniProt ID(s) linked to protein names, which in effect are the representatives of the vaccine candidates listed in Supplementary Table S4. UniProt IDs underlined exactly match to the protein identifier in the publication. UniProt IDs in bold are incorrect with respect to the protein name e.g., Q9GSA3 is the ID for ‘merozoite surface protein’ and not ‘merozoite surface protein 1’; Species = the source species for the proteins; Prot. = ‘Yes’ or ‘No’ whether the publication reports testing in an animal model for protein immunogenicity.

^a^No formal identifier e.g., GenBank accession No. or UniProt ID given in publication. The UniProt ID shown is based on the protein name only.

^b^Protein reported in other publications as a possible vaccine candidate.

**Table 3:** Manual verification of vaccine candidates derived from automated extraction of names from published abstracts.

Candidate	UniProt ID	H	PubMed ID	Year	Species	Prot
Circumsporozoite protein	A0A4V0KF74 Q03752 A0A077Y0S6	80	22252877	2011	*Plasmodium. yoelii*	Yes
Citrate synthase	False positive	27	8817831	1996	*Plasmodium berghei*	No
Microneme protein MIC3	*B2D1U3*	20	21632181	2011	*Toxoplasma gondii*	Yes
Liver stage antigen	Q25893	15	8609407	1996	*Plasmodium falciparum*	Yes
Rhoptry associated protein-1	A7AS21	12	12933845	2003	*Babesia bovis*	No^a^
Putative kunitz-type protease inhibitor	A0A3Q0KUE0 A0A3Q0KFV5 A0A5K4F6V0 *G4VEE0* A0A3Q0KN03 G4VBB1 G4VED8	1	31736947	2019	*Schistosoma mansoni*	Yes
Ribonuclease T2	*Q6PYW1*	1	28212670	2017	*Schistosoma japonicum*	Yes
Thioredoxin-like	A0A1N6LW58	1	29335000	2018	*Babesia microti* (strain RI)	No
Hydatid disease diagnostic antigen P-29	*Q9U8G7*	0	32908913		*Echinococcus granulosus*	Yes
Surface antigen 22	*Q70CC3*	0	33689009	2021	*Eimeria tenella*	Yes

Candidate = a protein name taken from sheet [Unique Names] in Supplementary Table S4. This sheet lists 438 unique names from the 1099 representative names extracted from the 1776 positively classified abstracts. The 324 unique names without a warning are considered possible vaccine candidates. Candidates with the highest and lowest h-indexes were chosen from the list at regular row intervals for manual verification; UniProt ID. = UniProt ID(s) linked to candidate name (names are mapped via sheets [Representative Names] and [Clusters] in Supplementary Table S4). Multiple IDs per candidate are representatives from different clusters. UniProt IDs underlined exactly match to the protein identifier in the publication; H. = h-index of the protein names’ source publications; Year = year of publication; Species = the source species for the protein; Prot. = ‘Yes’ or ‘No’ whether the publication reports testing for protein immunogenicity in an animal model.

^a^Protein reported in other publications as a possible vaccine candidate.

### Comparison of representative sequences

Parasites are composed of thousands of proteins. Theoretically, any protein from a parasite irrespective of its normal location could potentially trigger a response when presented to the immune system. This is because all parasite proteins are ultimately foreign material to the host. It is currently impractical to investigate all proteins for their vaccine candidacy potential. A compromised strategy is therefore to identify those proteins more ‘likely’ to induce an immune response and consequently more worthy of laboratory investigation. A protein that is either external to or located on, or in, the membrane of a pathogen are strong indicators that it is more likely to be accessible to surveillance by the immune system than a protein within the interior of a pathogen^32^. Focusing on only proteins naturally exposed to the immune system greatly narrows down the candidates for investigation. It must be acknowledged, however, that this strategy excludes potential candidates from the interior. For example, peptides from interior proteins can be presented to the immune system via MHC Class II molecules on professional antigen presenting cells (APCs) that have engulfed and digested protozoan parasites.

Characteristics of the 1099 representative proteins were extracted from existing resources or predicted from their sequences using freely available bioinformatic programs. Supplementary Table S5 (sheet [Representative proteins]) lists 96 characteristics collected for each representative protein. Note that characteristics sourced from resources that were unavailable are denoted by ‘na’. A primary aim of the characteristics collection was to provide circumstantial evidence of a protein’s natural accessibility to the immune system. Each protein was scored based on the number of accessibility indicators e.g., indicators such as presence of transmembrane (TM) domains, a signal peptide (SP), glycosylphosphatidylinositol (GPI) anchors, published epitopes; subcellular locations related to membrane or secreted; and Gene Ontology (GO) terms related to pathogenesis (see section ‘Calculating immune system accessibility score’). The accessibility score in addition to the ‘questionable’ flag provided a reliability guideline of a representative protein’s potential as a vaccine candidate; only a guideline because many of the indicators are predictions. With this in mind, we propose that the 152 representative proteins with no accessibility indicators and associated with questionable publications (i.e. more than 5 years old with h-indexes less than or equal to one) are the least reliable. However, proteins with the highest accessibility scores are not necessarily more worthy than a protein with only one accessibility indicator. Of the 1099 proteins, 62.5% have accessibility scores greater than zero. Grouping these proteins under phyla, Apicomplexa proportionally contains the most reliable proteins (77.3%), then Nematoda (52%), Euglenozoa (49.3), Platyhelminthes (41%), and Arthropoda (25%).

**Figure 6:**
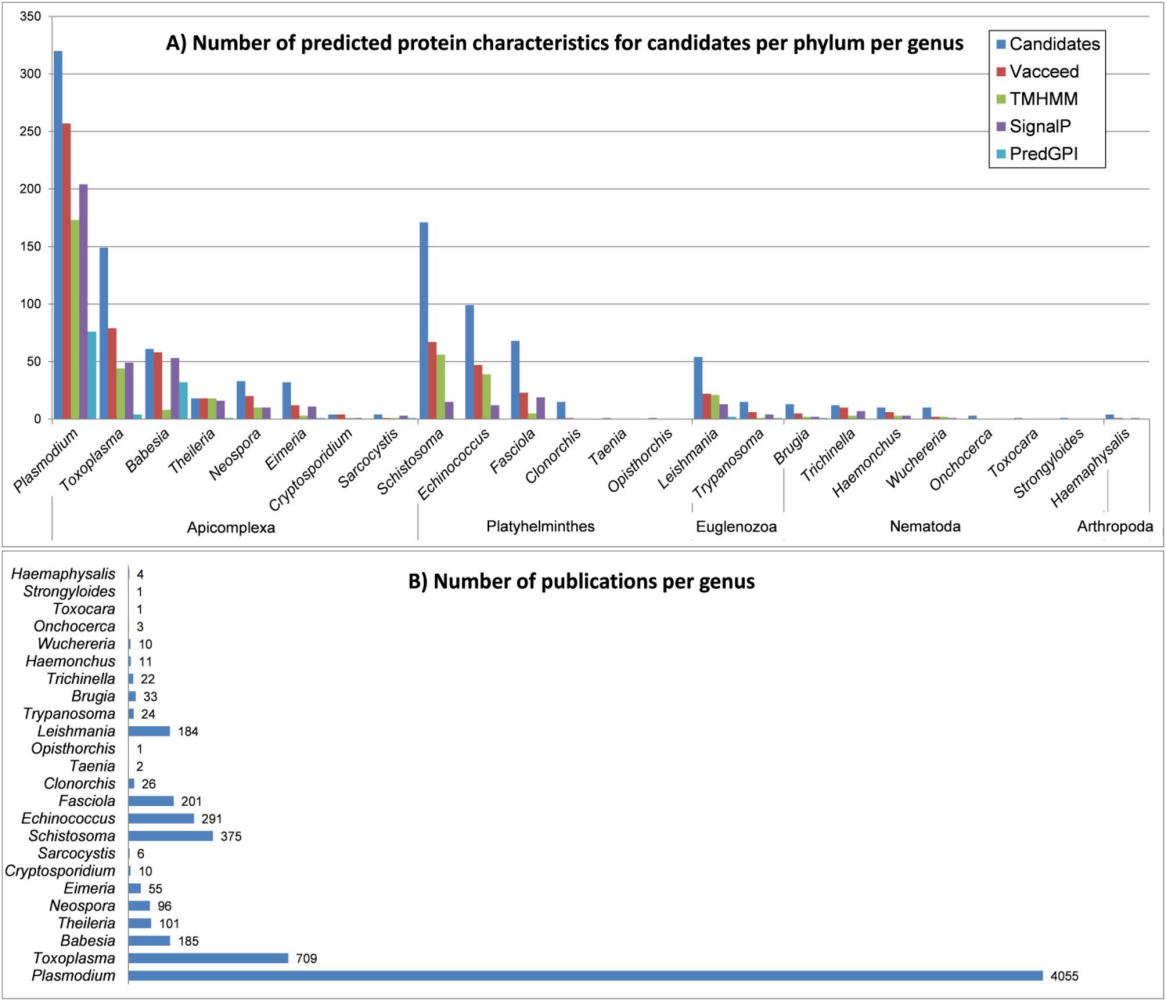
A column graph depicting the number of predicted characteristics in candidate proteins per phylum per genus (**A**); and a bar graph showing the number of publications associated with the candidates (**B**). Protein characteristics were predicted from 1099 representative sequences related to protein names extracted from 332,627 PubMed ‘title+abstract’ texts using the presented study’s pipeline. The 1099 proteins are considered here as potential vaccine **candidates**. Characteristics predicted are accessibility to the immune system by **Vacceed**, transmembrane (TM) domains by **TMHMM**, the presence of a signal peptide (SP) by **signalP,** and glycosylphosphatidylinositol (GPI) anchors by **PredGPI**. As an example of how to interpret the graphs, there are 1099 candidates of which 320 are proteins from the genus *Plasmodium* (a member of the Apicomplexa phylum)—257 of the 320 proteins are predicted to be naturally accessible to the immune system, 173 have at least one TM, 204 have SPs, and 76 GPI-anchors. The 320 candidates appear collectively in 4055 publications.

The characteristics collection also provided a resource for comparative analysis between the representative proteins (i.e., potential vaccine candidates)—see Fig. 6. Supplementary Data S4 presents via comparative tables and graphs, a candidates breakdown into phylum and/or genus groups for a selection of characteristics such as number of publications, infectious disease, average protein lengths, subcellular location, TMs, SPs, SP cleavage sites, GPI-anchors, published epitopes, and GO terms.

The 1099 representative proteins were further filtered on accessibility score and h-index thresholds to create two vaccine candidate groups: exposed and non-exposed (see Supplementary Table S5 sheets [Exposed candidates] and [Non-exposed candidates]). The exposed group contains 485 proteins. These proteins are considered the ones most likely to be naturally exposed to the immune system. The non-exposed group contains 121 proteins normally located in the interior of the cell. Proteins in both groups have prominent publication histories as determined by the h-index. Note that within each group many protein names are identical, but they are considered here as separate proteins because their sequences have a less than 90% similarity. Furthermore, the same name can appear in both groups. This inconsistency is addressed in the [Non-exposed candidates] sheet with a column called ‘Name type’, which contains ‘Unique’, ‘Non-exposed common’, ‘Exposed common’, or ‘Common’ with respect to the protein name—where ‘Unique’ is a name exclusive to the non-exposed group (e.g., Nucleoside hydrolase); ‘Non-exposed common’ is a name appearing in both groups but is more common in the non-exposed group (e.g., Paramyosin); conversely, ‘Exposed common’ is a name appearing in both groups but is more common in the exposed groups (e.g., Heat shock protein 70); and ‘Common’ is a name occurring equally in both groups. In this study, the sequence and the protein characteristics it encodes takes precedence over the name, which may contradict expectations when given a name. For example, the names ‘Surface antigen 2B’ and ‘Apical membrane antigen’ imply that their sequences belong to the exposed group. Nevertheless, the proteins associated with these names (UniProt IDs B9VQX5 and R4JAM5) are classified as non-exposed because their sequences provide no evidence of natural exposure to the immune system.

### Predicting vaccine candidates using an in silico approach

As one example of how the collated candidates from this study can be used for identifying further vaccine candidates, the program Vacceed^33^ was used. This program is a high-throughput computational platform designed to automate the process of predicting, for eukaryotic pathogens, those proteins most likely to be naturally exposed to the immune system. A crucial component of Vacceed is an ensemble of supervised ML algorithms for binary classification that can be trained. The 485 proteins identified here as exposed candidates were used as the source for the positives training data. In contrast, 485 proteins with predicted cytoplasmic and nuclear subcellular locations formed the negatives—see Supplementary Table S6 sheet [Negatives] and later ‘Predicting vaccine candidates for three apicomplexan species using Vacceed’ that describes the negatives creation. Based on a tenfold cross-validation of training data, the classification accuracy is 89.3% (see Supplementary Table S6 [Vacceed performance measures] for other measures used to evaluate Vacceed’s predictive performance).

Every known protein for *Plasmodium falciparum* (strain 3D7), *Toxoplasma gondii* (strain ME49), and *Babesia bovis* (strain T2Bo) were processed through Vacceed to predict their probability of being exposed to the immune system. All three species are apicomplexans. *Plasmodium* causes malaria (the most fatal parasitic disease); *T. gondii* causes toxoplasmosis (responsible for birth defects in humans) and considered the model organism for Apicomplexa^34^; and *B. bovis* causes babesiosis, a significant economic disease burden to livestock industries^35,36^*.*

Supplementary Table S6 lists the predicted probabilities for the three apicomplexan species. The reliability of annotated protein names is considered poor by the current study (see later Annotation analysis). The high scoring probabilities presented must therefore come with a caveat that their names may be misleading with regard to their sequence signals encoded and true function. The *Plasmodium* list has 354 unique names associated with 704 proteins having a greater than 90% probability of being correctly classified as naturally exposed to the immune system. Note that 582 of these 704 proteins are predicted, 102 are inferred from homology, 19 have evidence at the protein level, and 150 have ‘putative’ in the name. Notable in the 354 names are those reported to have been explored as vaccine candidates, namely: apical membrane antigen 1^37^, merozoite surface protein 1^38^, merozoite surface protein 3^39^, circumsporozoite protein^40^, glutamate-rich protein GLURP^41^, serine repeat antigen 5^42^, erythrocyte binding antigen-175^43^, gpi-anchored micronemal antigen^44^, sporozoite invasion-associated protein 2^45^, thrombospondin-related anonymous protein^46^, cell traversal protein for ookinetes and sporozoites^47^, liver stage antigen 3^46^, liver stage antigen 1^48^, rhoptry neck protein 2^49^, cytoadherence linked asexual protein 3.1^45^, 6-cysteine protein P12/P38^50^, and duffy binding-like merozoite surface protein^45^. Notable names explored as candidates but classified here with a less than 90% probability were sporozoite threonine and asparagine-rich protein (82.7%)^51^, merozoite surface protein 2 (80%)^52^, rhoptry-associated leucine zipper-like protein 1 (75.3%)^53^, reticulocyte binding protein homologue 5 (45.5%)^54^, ring-infected erythrocyte surface antigen (60.3% and 38.8%)^55^, and erythrocyte membrane protein 1 (PfEMP1)^56^. There are 61 PfEMP1 proteins and all were less than 70% (25 less than 50%). PfEMP1 proteins do not contain signal peptides and are exported by distinct non-classical secretion pathways^56^, which may explain the low Vacceed scores. Of the 704 proteins, 144 are named ‘conserved protein, unknown function’. These proteins of unknown function are considered promising candidates worth exploring because they have characteristics encoded in their sequences similar to those proteins previously explored.

The *Toxoplasma* list has 393 unique names associated with 1022 proteins having a greater than 90% probability of being correctly classified as naturally exposed to the immune system (582 of the 1022 proteins are predicted, 153 inferred from homology, 2 have evidence at the protein level, and 52 have ‘putative’ in the name). Notable names previously explored as vaccine candidates are dense granule proteins GRA2 to GRA8, GRA14 (GRA6 an exception with 85.9%)^57–61^; Microneme protein MIC2^62^; MIC3^63^; Protein disulfide-isomerase^64^; MIC12, SAG-related sequence SRS13, and Rhoptry protein ROP6^65^. Notable names explored as candidates but classified here with a less than 90% probability are SAG-related sequence SRS29A (89.9%)^65^, MIC1 (88.8%)^66^, MAG1 (87.7%)^67^, Rhoptry protein ROP18 (77.4%)^64^, ROP1 (40.4%)^68^, Profilin (1.3%)^69^. Of the 1022 proteins, 474 are named ‘Uncharacterized protein’.

The *Babesia* list has 106 unique names associated with 435 proteins having a greater than 90% probability of being correctly classified as naturally exposed (279 of the 435 proteins are predicted, 100 with evidence at transcript level, 56 inferred from homology, and 153 have ‘putative’ in the name). Notable names previously explored are 12D3 antigen^70^, merozoite surface antigen (MSA) 1 and 2^71,72^, thrombospondin-related anonymous protein^73^, spherical body protein 2 (SBP2)^74^. A notable name explored as candidate but classified here with a less than 90% probability is rhoptry-associated protein 1 (RAP-1) (77.4%)^75^. Proteins that are exported from *Babesia* into the host’s red blood cell (RBC) cytoplasm and/or the RBC membrane are considered worthy vaccine candidates^76^. Variant erythrocyte surface antigen (VESA) proteins are the main known *B. bovis* RBC surface-exposed proteins^77^. There are 133 VESA proteins, nine have a greater than 90%, 79 between 50 and 90%, and 45 less than 50% probability of being naturally exposed. Small open reading frame (smORF) proteins are proposed to play a role in sequestration together with VESA proteins^78^. Sequestration is the attachment of parasitised RBCs to ligands on endothelial cells of blood vessels. There are 44 smORF proteins, 41 have a greater than 90% probability of being exposed (the three exceptions have probabilities: 89.8%, 85.9%, and 7.1%). Of the 435 proteins, 39 are named ‘Uncharacterized protein’, 126 ‘conserved hypothetical protein’, and 107 ‘membrane protein, putative’.

Only laboratory testing can verify that the proteins with predicted high probabilities are truly commendable for continued investigation. Given the thousands of proteins constituting a pathogen, the purpose of the lists is to highlight the more worthy ones for laboratory testing. The expectation is that a reduced list will ultimately save time and money over the traditional identification approach.

## Discussion

Previous studies^22,23^ have shown that reverse vaccinology can be improved with a machine learning approach. Crucial requisites to this approach are quantity and quality training data consisting of positives and negatives. Ideally, positives are sourced from hundreds of proteins shown to induce a protective immune response in a host. The catalyst for this study was a need for positives to conduct an in silico vaccine discovery approach against *Babesia bovis.* A search for single or multiple public repositories that might contain a sufficient quantity of *B. bovis* positives was not forthcoming. The only repository distinguishing proteins with immunogenic potential is Protegen with currently 167 unique parasitic antigens, including five for *B. bovis.* Given there are hundreds of parasite species causing infectious diseases (see Supplementary Table S7 for examples), it is reasonable to assume there are hundreds, theoretically thousands of immunogenic proteins to be discovered. An unknown number of these proteins have already been discovered and published. However, as our unsuccessful search highlighted, there is no single resource listing all the discovered proteins. Our motivation for this study was the lack of such a list and the knowledge that research publications collectively contain an unexploited pool of potential immunogenic proteins.

PubMed, a major search engine for accessing publication abstracts, contains over 30 million abstracts on life sciences and biomedical topics. It provides an advanced search builder to query text within each publication’s abstract with terms and logical operators e.g., AND, OR, and NOT. The challenge is in determining the optimum query terms and logic to return the most pertinent publications. In our instance, abstracts containing a reference to an immunogenic protein. For example, PubMed returns 332,627 abstracts released over the last 30 years containing either the word ‘parasite’, ‘vaccine’, ‘vaccinated’, OR ‘vaccination’ when using the ‘title/abstract’ term. Including more appropriate keywords such as immunogenic or antigen returns 830,107. This number can be reduced by using the ‘AND’ logic such as parasite AND immunogenic, but at the cost of incorrectly enforcing a notion that every abstract of interest contains these words. Furthermore, there is no obvious indication given 1000 s of returned abstracts that they truly contain immunogenic parasite proteins. Regrettably, no standardised set of keywords for summarising immunological studies has been adopted over the last 30 years. Our conclusion is that PubMed searches alone are an impracticable option towards obtaining abstracts of interest.

The aim of this study was to ‘automatically’ identify all parasite proteins that induce a protective response in an animal model as reported in the scientific literature within the last 30 years. More specifically, the aim was to develop a pipeline that takes PubMed-derived ‘title+abstract’ text as input, and then outputs a list of vaccine candidate proteins that can be linked to their sequences. The developed pipeline comprised two linked stages—first, classification of abstracts using ML, and second, protein name extraction from classified abstracts using a hybrid of NLP, rules, and database searching.

Training data was required for the abstract classification. We were faced with a similar cyclic conundrum to the one initiating the catalyst for this study. That is, a sufficient number of abstracts—verified to contain a parasite protein ‘shown to induce a protective response in an animal model’—were required for the training data to predict the classification of unseen abstracts. A rule-based approach using keyword selection criteria provided the sufficient number. Given 332,627 PubMed ‘title+abstracts’, the rule-based approach identified 2744 abstracts of interest i.e., positives. A manual assessment of 100 randomly chosen abstracts from the 2744, suggested the rule-based approach has an 85% accuracy of selecting an abstract of interest, but only 80% in its capacity to fully identify the relevant protein name. The manual assessment, however, could not provide an indication of how many abstracts of interest from the 332,627 were missed. We speculate that many were missed due to the restrictive nature of a fixed set of keywords underlying the rule-based approach. This issue of missed important abstracts is not unlike that encountered through PubMed advanced search builder.

The pipeline ML training data comprised 1556 positives and 1556 negatives obtained from the highest and lowest scoring rule-based derived abstracts, respectively. An advantage of the ML over the rule-based approach is that every word in the ‘title+abstract’ other than stop words are considered during the classification process rather than only a fixed set of keywords. That is, the ML classifier recognizes distinguishing patterns between positive and negative abstracts that can be used to detect similar patterns in unseen abstracts. This considerable advantage overcomes the ‘missed abstracts’ issue. The classification accuracy as determined from tenfold cross-validation of the training data was 99.6%. This high accuracy is acknowledged, nonetheless, as misleading because the categorisation of training data abstracts was determined by the rule-based approach. An unknown 15% of this categorisation is potentially incorrect based on the manual assessment. As a more reliable performance indicator, the accuracy in correctly classifying ‘verified’ abstracts independent of training data was 83% with a sensitivity and specificity of 98.8% and 71.3%, respectively. A threshold of 0.5 was applied to predicted probabilities to determine a positive or negative classification. The threshold technically can be changed to alter the sensitivity and specificity ratio. In this study, we deemed that a higher sensitivity than specificity was preferable i.e., have more false positives than false negatives filtering to the next stage of protein name extraction. Approximately 22% of the 332,627 PubMed ‘title+abstracts’ have a classification probability greater than 0.5. We expect at least 15% (10,976) to be false positives.

The second pipeline stage, protein extraction, presented the most challenges. To reiterate, the extraction goal was to determine whether any word or ‘series of words’ in the classified abstract matched a known gene or protein in UniProt and/or NCBI databases. The challenges were because (1) abstracts rarely contain a protein’s unique identifier (e.g., UniProt ID and NCBI accession #), which would unambiguously provide the link to the protein associated with the abstract’s study. Protein or gene names in UniProt and NCBI are not unique (hence the reason for IDs); (2) many proteins and genes are poorly annotated with single names like ‘protein’, ’antigen’, ‘putative’, ‘raw’, ’dead’, ‘sand’, ‘impact’ and a multitude of other names with an English language meaning; (3) names in abstracts and databases have inconsistency in spelling and/or capitalising names e.g., ‘*Apcial* membrane antigen I’; (4) proteins that are possibly the same have slight name variations in the abstract and/or databases e.g., Rhoptry associated protein-1 and Rhoptry-associated protein 1 are recorded as separate proteins in UniProt; and (5) all protein names extracted from abstracts are not necessarily immunogenic proteins.

In an attempt to overcome the protein extraction challenges, we used spaCy to build a custom NER model, trained specifically to extract gene and protein name entities. The NER model was trained with protein names from the training positives used to build the abstract classifier. Testing the custom NER model with the task of protein extraction on verified abstracts showed that it identifies expected names with less false positives but more false negatives than a rule-based approach. Notably, every protein and gene was correctly identified as an entity in an abstract if they also appeared in the training data, whereas other valid proteins and genes in the abstract were sometimes missed if not used in the training data. We propose that the number of false negatives can be greatly reduced by including more protein names in the NER training data. Ideally, the training data should contain a BIO tag for every parasite protein and gene name in the UniProt and NCBI databases. These databases represent the entire real world domain for protein name extraction, which means it would be impossible to overtrain the NER model given the ideal training data.

The final output from the entire pipeline given 332,627 was 1099 protein names and their associated sequences. An important point is that this output number is variable due to the user-defined thresholds governing the final output e.g., separate thresholds can be applied to the ML abstract classification probability, CD-HIT identity, number of publications, number of animal models, and start and end year of publications. We acknowledge that it is debatable whether the most appropriate thresholds were used, but our aim was to obtain the optimum acceptable level of false predictions i.e., to find the optimal sensitivity and specificity. For example, both sensitivity and specificity would both be 100% in a perfect system. Changing thresholds in our real world system increased sensitivity whilst decreasing specificity, or conversely, decreased sensitivity while increasing specificity. Our considered optimum was the maximum obtainable sensitivity and specificity but with a higher sensitivity than specificity i.e., more false positives than negatives for further evaluation.

The 1099 proteins were further filtered into two vaccine candidate groups, exposed and non-exposed, based on a protein’s normal location in the context of the entire parasite. The ‘exposed’ candidates are those proteins naturally exposed to the immune system i.e., membrane-associated or secreted, and ‘non-exposed’ are hidden within the cell interior. The groups were determined from circumstantial evidence of a protein’s accessibility to the immune system i.e., collected or predicted protein characteristics indicating a protein’s potential to be in view of a host’s immune system surveillance. Furthermore, proteins calculated here with high rather than low h-index were considered more favourably. Taken together, accessibility indicators and h-indexes, the final list of proposed exposed candidates contained 485 proteins (363 Apicomplexa, 75 Platyhelminthes, 31 Euglenozoa, and 16 Nematoda). Although four arthropod proteins are in the 1099 representative names, all four had poor accessibility indicators and h-indexes and were consequently filtered from the candidate list. The unexposed group comprised 121 proteins with no accessibility indicators but high h-indexes (55 Platyhelminthes, 49 Apicomplexa, 11 Euglenozoa, and 5 Nematoda).

A vaccine is in effect a simulated infection to deceive the immune system into making memory B or T cell responses specific to an antigen. These memory cells then help protect against future infections from parasites possessing the same antigen i.e., provide a protective immune response. In the current study, we focused on naturally exposed immunogens under the premise that if they induced memory cells as a vaccine component, these memory cells are more likely to encounter the same immunogen during a real infection. We acknowledge that the benefits of reducing the number of candidates for investigation by maintaining this focus, comes at the cost of potentially missing important immunogenic peptides residing on interior proteins. This focus was further illustrated with a reverse vaccinology inspired program called Vacceed, which is designed to predict those proteins naturally exposed to the immune system. Probabilities of exposure were predicted for every protein from three important apicomplexan species (*P. falciparum*, *T. gondii*, and *B. bovis*) with the expectation 10% are incorrectly predicted given the training data tenfold cross-validation metrics; and the caveat that only laboratory testing can truly verify the predictions. The proteins with the highest probabilities are judged to be the optimum within the current quality constraints of protein sequences. Reverse vaccinology has the potential to rapidly advance vaccine development against parasites, but its potential has been hindered since its inception by the lack of verified protein sequences. Most parasite sequences are predicted from poor quality genomes. For example, a recent study^79^ reveals misassembly, karyotype differences, and chromosomal rearrangements of the *T. gondii* and *Neospora caninum* genomes following a re-evaluation. These genomes were originally sequenced using conventional Sanger sequencing technology^80^ i.e., first generation sequencing. Next-generation sequencing (NGS) has mostly superseded Sanger sequencing for genome research but has limitations caused by inadequacies of short-read outputs with repetitive regions scattered across the genome^81^. These limitations have caused assembly artefacts that are currently widely distributed in genome and proteome databases^82^. NGS is now regarded as second-generation sequencing. Third-generation sequencing (TGS), still under active development, has the capability to produce substantially longer reads than second generation sequencing. Reads that are longer than a repetitive region provides a solution to assembling long contigs spanning an entire genome. TGS potentially provides this solution but has a higher sequencing error rate and systematic error than NGS^83^. Nevertheless, the recent combination of NGS and TGS data with the advances in assembly technologies has resulted in greatly improved genome sequence quality, which is epitomised by the new TGS-derived *T. gondii* and *N. caninum* genomes^79^. An increase in quality parasite genomes ultimately equates to reverse vaccinology progressing towards its full potential.

It remains unclear how many parasite proteins reported to ‘induce a protective response in an animal model’ were missed by the pipeline. We expect, nevertheless, that our final candidate lists are far from complete given the limitations of the study’s pipeline. Given only abstracts, extracting a protein name that links to the exact protein evaluated in the related study was the foremost challenge e.g., a protein name is not a unique identifier to its true sequence. A pipeline limitation is that there is no measurable indicator that the CD-HIT clustering solution made the correct link. Another limitation was that in order to reduce the numbers of false positives, candidates reported in many publications are considered more favourably in the selection process than those reported in only one or two. This consideration potentially excludes a true and perhaps novel candidate that is under-reported. We conclude that the pipeline succeeded in capturing the low hanging fruit (i.e., the popularly reported candidates) with an 86–90% accuracy based on independent testing. It is unclear how many candidates were missed because the percentage of publications reporting immunogenic parasitic proteins tested on animal models is unknown. A key closing point is that the current alternative to the pipeline for identifying pertinent parasitic proteins for further investigation is a manually, time-consuming approach using judicious PubMed keyword searching and colossal amounts of reading.

## Materials and methods

### Computer platform used for study

All experiments and data generation was performed on a high performance computing (HPC) cluster node with 64 bit kernel, 32 MB memory, and 8 cores. The pipelines were designed for a Linux operating system and have only been tested on Red Hat Enterprise Linux 7.9, but are expected to work on most Linux distributions. Python version used was 3.6.8.

### Source code availability

Source codes indicated in this article as being provided are available via GitHub: https://github.com/goodswen/abstract_classification.

### Obtaining abstracts

PubMed was the source for all abstracts. There is a limit of 10,000 abstracts that can be downloaded from the PubMed webpage. The limitation was overcome using the Entrez Programming Utilities (E-utilities—https://www.ncbi.nlm.nih.gov/books/NBK25497—last viewed September 2021). The utilities provide an interface into the Entrez query. Entrez is a data retrieval system that provides users access to NCBI's databases such as PubMed. An in-house Python script (source code provided: ‘get_abstract.py’) used the module ‘Entrez’ from Biopython (version 1.79)^84^ to implement the E-utility interface.

All abstracts dating from 1991 to present that contained either the word ‘parasite’, ‘vaccine’, ‘vaccinated’, or ‘vaccination’ in the abstract or title text were downloaded in 10,000 batches using the in-house Python script. The search query was ((parasite[Title/Abstract]) OR (vaccine[Title/Abstract]) OR (vaccinated[Title/Abstract]) OR (vaccination [Title/Abstract])) AND (("1991"[Date-Publication] : "2022"[Date-Publication])).

The ‘Entrez.efetch’ return mode parameter (retmode) defined ‘text’ as the format of the PubMed retrieved data (XML is another option). The retrieved data contained more information than required e.g., author information. An in-house Python script (source code provided: ‘process_abstract.py’) created a more workable file that included only PubMed ID, Title, and Abstract per row for each publication (referred to henceforth as the Abstracts).

### Keyword files

A list of notable parasite species of medical and veterinary importance and their associated infection/disease was compiled—three classes of parasites with 75 classed as protozoan, 88 helminthic, and seven ectoparasite (170 in total—see Supplementary Table S7 sheet [Species + Diseases]. Its contents were compiled with reference to Centers for Disease Control and Prevention (CDC)—Parasitic Diseases (https://www.cdc.gov/parasites/), Protegen database (http://www.violinet.org/protegen/index.php, ParaBoss website (Australia's resource for parasite management information for sheep, goats and cattle—https://www.paraboss.com.au), and Meat and Livestock Australia (MLA) website on ticks (https://www.mla.com.au/research-and-development/animal-health-welfare-and-biosecurity/parasites/identification/ticks/. All websites were last viewed in September 2021. The compiled list is referred to henceforth as the ‘taxonomy_ disease_ link’ and is used in abstract scoring.

All diseases from ‘taxonomy_ disease_ link’ were extracted to create a unique list of diseases, referred to henceforth as the ‘Disease_keywords’ (see Supplementary Table S7). A list of common animal models was compiled, referred to henceforth as the ‘Animal_keywords’ (see Supplementary Table S7). An animal model denoted here is a non-human species used to evaluate protective immunity of a protein candidate when challenged with an infection. A list of frequently used words or terms when describing protective immunity was compiled and referred to henceforth as the ‘Protection_keywords’ (see Supplementary Table S7.

### Evidence abstracts

A conventional manual process was performed to find publications that met the following requirement: stated at least one parasite protein that induced a protective response in an animal model. For example, the process involved using PubMed search terms and keywords and/or finding pertinent publications through citations in other publications. References to publications were also obtained from the Protegen database.

### In-house parasite protein database creation

The files ‘uniprot_sprot_invertebrates.dat.gz’ (manually annotated and reviewed proteins –38.9 MB compressed) and ‘uniprot_trembl_invertebrates.dat.gz’ (automatically annotated and not reviewed proteins—8.3 GB compressed) were downloaded from: https://ftp.uniprot.org/pub/databases/uniprot/current_release/knowledgebase/taxonomic_divisions/. The uncompressed files were over 48.2 GB and contained all proteins associated with invertebrate organisms. An explanation of the keywords used in the files can be found in https://www.uniprot.org/docs/keywlist. An in-house Python script was used to parse the uncompressed files to generate one file with one protein per row under the main column headings: UniProt ID, Protein Name, and Taxonomy ID. Only proteins with a Taxonomy ID in the taxonomy_ disease_ link were extracted. In summary: 28,285 (reviewed) and 12,688,175 (unreviewed) invertebrate-related proteins were read, and 1,058 (reviewed) and 1,462,608 (reviewed) proteins linked to 174 Taxonomy IDs were recorded in a file referred to henceforth as ‘parasite_proteins’.

### In-house parasite gene database creation

The file ‘gene_info.gz (647 MB compressed, 4 GB uncompressed) was downloaded from: https://ftp.ncbi.nlm.nih.gov/gene/DATA/gene_info.gz. An in-house Python script was used to parse the uncompressed files to generate one file with one gene per row under the main column headings: Gene ID, Gene Name, and Taxonomy ID. Only genes with a Taxonomy ID in the taxonomy_ disease_ link were extracted. In summary: 26,379,168 genes were read, and 596,571 genes linked to 174 Taxonomy IDs were recorded in a file referred to henceforth as ‘parasite_genes’.

### Abstract classification using a rule-based approach

An in-house Python script (source code provided: ‘score_abstract.py’) classified an abstract based on whether it contained matching words to the following four criteria words: (1) genus or species names from taxonomy_ disease_ link; (2) Protection_keywords; (3) Animal_keywords, and (4) Disease_keywords. Abstracts with at least one count for each of the four criteria were classified as ‘abstracts of interest’. These abstracts were further processed to check if they contained words matching to protein or gene names in parasite_proteins and/or parasite_genes (see the following method).

### Protein name extraction using a rule-based approach

An in-house pipeline consisting of Linux and Python scripts were used to extract parasite protein or gene names from the abstracts of interest (pipeline source code provided: rule_based_protein_extraction). More specifically, the aim is to associate the extracted name with a UniProt ID or NCBI Gene ID.

The pipeline uses both parasite_proteins and parasite_genes for searches. parasite_proteins contains the following columns: UniProt ID, Protein name, Alternative name, NCBI Protein ID, NCBI Gene_ID, Gene name, Taxonomy ID; and parasite_genes contains: Taxonomy NCBI Gene_ID, Symbol, LocusTag, Gene Name. Note that ‘Protein name’ and ‘Gene name’ can consist of one or more words.

The pipeline incorporates two methods to obtain UniProt ID or NCBI Gene IDs. Steps in Method #1 for each abstract: (1) punctuation (e.g., []}{,.;)?!:-) are removed; (2) genus or species names from taxonomy_ disease_ link are checked for matches; (3) every ‘Protein name’ from parasite_proteins that is specific to the previously matched genus or species is checked for a match. An exception is that single word protein names matching to common words with English meaning in the abstract are ignored. For example, poorly annotated single names such as ‘protein’, ’antigen’, ‘putative’, ‘raw’, ’dead’, ‘sand’. A Python script included the module ‘enchant’, which provides functionality to check the spelling of words via in-built dictionaries of different languages. Here, when checking for single word protein name, any name in the enchant US dictionary or in ‘Ignored_names (see Supplementary Table S7) is ignored. Single word protein names not ignored but listed in Check_names (see Supplementary Table S7) an additional check is performed, whereby a name is accepted if in the abstract it is capitalised, or preceded or followed with one of the following words: ‘gene’, ‘protein’, or ‘antigen’; (4) a UniProt ID is recorded for all matches; (5) as per step #3, but every ‘Gene name’ from parasite_genes is checked for a match and a NCBI Gene_ID recorded for matches.

Steps in Method #2 for each abstract: (1) genus or species names from taxonomy_ disease_ link are checked for matches; (2) punctuation (e.g., []{},.;)?!:-) at start and end of words, numbers, words (191 in total) contained in Ignored_words (see Supplementary Table S7), and words in the enchant US dictionary except those capitalised, or preceded or followed with one of the following words: ‘gene’, ‘protein’, or ‘antigen’ are all removed. The ‘Ignored_words’ are essentially those considered stop words such as ‘the’, ‘a’, ‘that’, and ‘when’; (3) each remaining word in the abstract is checked for a match with UniProt ID, Alternative name, NCBI Protein ID, NCBI Gene_ID in parasite_proteins that is specific to the previously matched genus or species. Some abstract words are preceded by a two letter species abbreviation e.g., ncSAG1 indicating a SAG1 protein from *Neospora caninum*. When no match is found, an additional matching search is performed by removing the first two letters of the word if they are an abbreviation for the species in the abstract; (4) a UniProt ID is recorded for all matches; (5) as per step #3, but each remaining word is checked for a match with NCBI Gene_ID, Symbol, LocusTag in parasite_genes and a NCBI Gene_ID recorded for matches.

Note that the case of a word (i.e., upper or lowercase) was disregarded when determining a match. The pipeline combines the output from both methods to create a single file containing a list of unique UniProt IDs for each PubMed ID (where matched NCBI Gene IDs are mapped to UniProt IDs).

### Creation of machine learning training data

The 2,744 abstracts of interest generated by the rule-based approach were further filtered. An abstract was selected if it contained a parasite protein name or gene that is mentioned in more than five other publications and contained more than two ‘protective immunity’ keywords. An in-house Python script applied the thresholds (source code provided: ‘get_training_data.py’). Abstracts that exceeded the > 5 publications and > 2 keyword thresholds formed the positives training data (1556 in total).

The negatives training data was obtained by randomly selecting 1556 from 15,358 abstracts (from the initial 332,627 downloaded) that had a zero matching count to all words contained in the five keyword files (taxonomy_ disease_ link; Protection_keywords; Animal_keywords, Disease_keywords). Supplementary Table S2 lists the lists the PubMed IDs and their relative keyword counts for the positives and negatives training data.

As part of the ML feature extraction process, the title+abstracts associated with the training data PubMed IDs were converted to a vectorised format (i.e., a numerical representation) using the text vectorization technique, Bag of Words (BoW)^85^. In brief, the technique starts with a list of words (the BoW) that are considered important. The BoW in this instance contained all common words (minus stop words) from the positives’ training abstracts (1011 words). Then, given the ‘title+abstract’ text as input, the output for the training data was a numerical vector consisting of the frequency of each word from the BoW that occurred in the input e.g., for each ‘title+abstract’ input, the training data had 1011 values (features)+a 0 or 1 representing a negative or positive target. The BoW is listed in Supplementary Table S2.

### Abstract classification using machine learning

A pipeline comprising Linux and R scripts performed a binary classification of abstracts i.e., two classes: abstracts of interest (those containing a parasitic protein name shown to induce a protective response in an animal model) and abstracts to be ignored. The ML algorithm used to build the predictive models was support vector machines (SVM)^86^. SVM is a popular ML algorithm for text classification when the amounts of training data are limited^87^. This algorithm was implemented via the ksvm R function, which is contained in the kernlab package. The function used two arguments: a data frame of numeric variables (i.e., the vectorised training dataset) and a numerical class vector, i.e., a vector representing the target label, which had two classes: 1 (positive) and 0 (negative). Given input data (a vectorised ‘title+abstract from a publication), the SVM algorithm generates a probability for each class e.g., class positive = 0.75, class negative = 0.25. This means that if the abstract was classified as a positive, then the classification is considered to be 75% likely to be correct. An important caveat, however, is that a class positive equal to 1.0 does not necessarily mean the associated abstract is unquestionably an abstract of interest. Parameters were tuned empirically with the training and validation datasets. All default parameters for the SVM algorithm were used except for kernel = "rbfdot" and type = "C-svc".

An additional pipeline using the same ksvm R function was created to perform tenfold cross validation. A threshold of 0.5 was applied to the predicted probabilities, whereby a true positive is a probability >  = 0.5 and a false positive is a probability < 0.5 for expected positives, and similarly, a true negative is <  = 0.5 and a false negative is > 0.5 for expected negatives. The measures used to evaluated the SVM model’s predictive performance were accuracy, error rate (misclassification), true positive rate (sensitivity), false positive rate, true negative rate (specificity), precision (positive predictive value, and negative predictive value (see Supplementary Table S2 sheet [SVM performance measures] for formulae). Source code is provided for both pipelines called ‘classification pipeline’, and ‘classification pipeline CV’.

Note that when the classification pipeline was used on the Evidence and Manual abstracts for evaluation, any input PubMed IDs matching the training data PubMed IDs were removed prior to the evaluation.

### Protein name detection using natural language processing

The program spaCy 3.1 was used to build a custom named entity recognition (NER) model. The first step was to convert the abstracts previously used for the ML training positives into an input format suitable for spaCy. This format comprises tagged sentences and the tagging style used was BIO (Beginning, Inside, Outside) e.g., three labels (B, I, O) are used to define the required entity boundaries. For this study, the required entities are protein and gene names e.g., The [O] protein [O] name [O] is [O] **dense** [B] **granule** [I] **protein** [I] and [O] the [O] symbol [O] is [O] **GRA14** [O] in [O] the [O] species [O] *Toxoplasma* [O] *gondii* [O]; where ‘dense granule protein’ and ‘GRA14’ are the required entities to extract. The source code to add tags to sentences is provided via the Python script ‘get_bio_format.py’ (output file: ‘bio_format.iob’); where the tags are protein and gene names extracted from the training data using the rule-based approach (source code: ‘get_bio_words.py’), and the sentences for tagging are those only containing a protein or gene name (obtained with source code: ‘get_sentences.py’).

The next step was to convert the BIO formatted sentences (‘bio_format.iob’) into a spaCy binary format: python -m spacy convert -c iob bio_format.iob/train (use ‘python -m spacy convert –help’ for details on syntax). To create a trained NER model: python -m spacy train acc_config.cfg—output/train/output (use ‘python -m spacy train—help’ for details). Note that the computational time to build the model is multiple hours e.g., over 24 h to model 5099 lines of BIO formatted sentences using the study’s computer platform. The built NER model was named ‘custom_NER_model’ and is provided.

The final step is a Python script (source code provided: ‘NER_protein_extract.py’) that takes ‘title+abstract’ per PubMed ID as input. The script uses the module ‘spacy’ to load the custom NER model, which determines the entities within the input text. Each entity is checked for a match in parasite_proteins and parasite_genes. The output file contains a list of matched protein and gene names for each PubMed ID.

### Calculating h-index and g-index

The h-index (also known as Hirsch index) is a leading author-level research metric, where ‘h’ denotes the number of papers having at least an ‘h’ number of citations^88^. In this study, the protein is the equivalent of the author. For example, a protein name is extracted from seven publications. The number of citations for each of the seven publications in descending order is 130, 85, 24, 15, 9, 4, and 1. In this example, the protein has an h-index of 5, meaning there are five publications with more than 5 citations i.e., the protein does not have six publications with 6 or more citations. The h-index is insensitive to the highly cited work^89^ e.g.; the h-index does not increase if the top five cited publications in the previous example continue to be cited. The ‘g-index’ allocates more weight to highly cited papers. The g-index is defined as ‘the largest number such that the top ‘g’ publications received together at least g^2^ citations^90^ e.g., the g-index for previous example would be 7, since the sum of the seven citations (268) is more than 49 (i.e. 7^2^).

### Collecting and predicting characteristics for representative proteins

The column heading names of the 96 characteristics collected or predicted are listed in the ReadMe file of Supplementary Table S5. The source and/or description for each column are now described:

[1] UniProt ID from UniProtKB; [2] Entry_name from UniProtKB; [3] Cluster # from study’s CD-HIT analysis; [4] Representative Protein Name is the majority protein name in Cluster #; [5] Warning (0 or 1) where ‘1’ indicates a questionable protein were the h-index is less than or equal to one and its associated publication is more than 5 years old; [6] accessibility score calculated in-house (see section ‘Calculating immune system accessibility score’; [7] class of parasite (ectoparasite, helminthic, or protozoan); [8] phylum (Apicomplexa, Arthropoda, Euglenozoa, Nematoda or Platyhelminthes) from NCBI Taxonomy; [9] number of publications with reference to the representative or related protein (calculated in-house); [10] citation counts from Scopus; [11, 12] see section on ‘Calculating h-index and g-index’; [13–16] publication years from PubMed; [17–20] organism, protein length, gene names, and protein existence from UniProtKB; [21, 22] diseases (see section ‘Keywords’); [23] TMs predicted by Phobius (1.0)^91^; [24] TMs predicted by TMHMM (2.0)^92^; [25–27] TM domains from UniProtKB; [28] SP predicted by Phobius (1.0); [29] SP predicted by TargetP (2.0)^93^; [30, 31] SP and cleavage site predicted by signalP (5.0)^94^; [32] SP from UniProtKB; [33] Vacceed^33^; [34–36] GO terms from UniProtKB; [37, 38] PredGPI (version 1.0)^95^. Note GPI FPrate < 0.001 is highly probable, < 0.005 is probable, < 0.01 is weakly probable, and >  = 0.01 is not GPI-anchored; [39] Subcellular location from UniProtKB; [40–44] Immune Epitope Database and Analysis Resource (IEDB)^96^; [45–96] all taken from UniProtKB.

### Calculating immune system accessibility score

An in-house Python script calculated the immune system accessibility score, which represents a protein’s potential ‘accessibility to the immune system’ based on a selection of the 96 characteristics previously described. For each representative protein, a ‘1’ is added for each immune system accessibility indicator (in this case a particular column entry from Supplementary Table S5) if a SP, TM, GPI-anchor is present in the protein, and if it has a subcellular location related to membrane and/or secretion; and a GO term related to pathogenesis. More precisely, inputs to the script were values from 12 columns: phobius_TM, TMHMM, TMCount, phobius_SP, targetP, signalP’ Vacceed, GO biological process, GO cellular component, GO molecular function, GPI_Desc, Subcellular location, NoOfEpitopes; where a ‘1’ is added for a true case in each of the following conditional statements: (1) if phobius_TM > 0 and TMHMM > 0 and TMCount > 0; (2) if phobius_SP > 0 and targetP >  = 0.5 and signalP >  = 0.5; (3) if Vacceed >  = 0.5; (4) if "GPI", "adhesion", "pathogenesis", or "immune" in GO biological process; (5) if "cell surface", "component of membrane", "extracellular", or "GPI-anchor" in GO cellular component; (6) if "cell surface" or "transmembrane" in GO molecular function; (7) if GPI-anchor description not equal to "Not GPI-anchored"; (8) if "membrane", "GPI-anchor", "surface", or "secreted" in Subcellular location; and (9) if ‘number of published epitopes’ > 0.

### Predicting vaccine candidates for three apicomplexan species using Vacceed

All 5460 *P. falciparum* (strain 3D7) and 8322 T*. gondii* (strain ME49) proteins were downloaded in a FASTA format from PlasmoDB (release 47) and ToxoDB (release 47), respectively, which are database members of Eukaryotic Pathogen Databases (EuPathDB)^97^. All 3706 currently available protein sequences for *B. bovis* T2Bo were downloaded in a FASTA format from PiroPlasmaDB (release 47), which is also a database member of EuPathDB.

Vacceed was downloaded from https://github.com/goodswen/vacceed/releases. The original training data was replaced with positives formed from the 485 proteins identified in this study as potential candidates, and negatives formed from 485 proteins with known and/or predicted cell interior locations such as the cytoplasm or nucleus—see Supplementary Table S6 sheet [Negatives]. The protein locations were determined by first downloading tabbed results from an advanced UniProtKB search using terms ‘organism:plasmodium OR toxoplasma’ and including Subcellular location [CC] as one of the output columns. An in-house Python script then parsed the results and selected 485 proteins that included in the Subcellular location column, ‘Cytoplasm’, ‘Nucleus’, or ,for a UniProt reviewed protein, a location within the interior of the cell e.g. Golgi apparatus, Lysosome. The new training data, formatted for Vacceed and named ‘train_profiles’, was copied into the expected Vacceed’s directory structure: /vacceed/*species_name*/pipeline/evidence/training_files, where ‘*species_name’* is a user-defined name for the target species such as plasmodium, toxoplasma, or babesia. The new ‘train_profiles’ is provided via GitHub: https://github.com/goodswen/abstract_classification.

Note that many of the training proteins were also proteins in the three apicomplexan species. In such cases, their probabilities were independently determined by running Vacceed with a training dataset that excluded the training protein for which the exposure probability was sought e.g., Vacceed in effect was executed with only one input training protein sequence at a time whereby the same protein was removed from the training data.

### Annotation analysis

UniProtKB provides a heuristic measure of the annotation, although the curators claim they cannot define the 'correct annotation' for any given protein (https://www.uniprot.org/help/annotation_score: last viewed October 2021). UniProtKB assign an annotation score from one to five to every protein, where five is considered the best-annotated entry (annotations with experimental evidence score higher than equivalent predicted/inferred annotations). With an understanding UniProtKB annotation scores are only a guideline of annotation quality, we checked scores for all 5460 *P. falciparum* (strain 3D7), 8322 T*. gondii* (strain ME49), and 3706 *B. bovis* T2Bo proteins (see Supplementary Table S6 for annotation score per protein).

Considering all *P. falciparum* proteins*:* 70.2% scored 1, 23.8% scored 2, 5.0% scored 3, 0.5% scored 4, and 0.5% scored 5; *T. gondii:* 86.9% scored 1, 12.0% scored 2, 1.0% scored 3, 0.1% scored 4, and 0.01% scored 5; and *B. bovis:* 86.5% scored 1, 12.4% scored 2, 1.0% scored 3, 0.05% scored 4, and 0% scored 5.

UniProtKB also indicates the type of evidence that supports the existence of the protein (see https://www.uniprot.org/help/protein_existence: last viewed October 2021). However, this is not an indication of the accuracy or correctness of the protein sequence. Considering all *P. falciparum* proteins: 72.4% predicted, 22.5% inferred from homology, 0.1% with experimental evidence at transcript level, and 4.9% with experimental evidence at protein level; *T. gondii*: 78.5% predicted, 21.3% inferred from homology, 0.02% with experimental evidence at transcript level, and 0.13% with experimental evidence at protein level; and *B. bovis*: 57.0% predicted, 20.4% inferred from homology, 22.4% with experimental evidence at transcript level, and 0.13% with experimental evidence at protein level (see Supplementary Table S6 for protein existence per protein).

## Supplementary Information

Supplementary Information 1.

Supplementary Information 2.

Supplementary Information 3.

Supplementary Information 4.

Supplementary Information 5.

Supplementary Information 6.

Supplementary Information 7.

Supplementary Information 8.

Supplementary Information 9.

Supplementary Information 10.

Supplementary Information 11.

## References

[1] Frank SA (1996). Models of parasite virulence. Q. Rev. Biol..

[2] Prenter J, MacNeil C, Dick JTA, Dunn AM (2004). Roles of parasites in animal invasions. Trends Ecol. Evol..

[3] Price PW (1980). Evolutionary biology of parasites. Monogr. Popul. Biol..

[4] Poulin R, Morand S (2000). The diversity of parasites. Q. Rev. Biol..

[5] May RM (2007). Parasites, people and policy: Infectious diseases and the Millennium Development Goals. Trends Ecol. Evol..

[6] Stothard JR, Adams E (2014). A preface on advances in diagnostics for infectious and parasitic diseases: Detecting parasites of medical and veterinary importance. Parasitology.

[7] Rappuoli R, Mandl CW, Black S, De Gregorio E (2011). Vaccines for the twenty-first century society. Nat. Rev. Immunol..

[8] Bloom DE, Cadarette D (2019). Infectious disease threats in the twenty-first century: Strengthening the global response. Front. Immunol..

[9] Chapman HD (2000). Practical use of vaccines for the control of coccidiosis in the chicken. Worlds Poult. Sci. J..

[10] Reichel MP, Ayanegui-Alcérreca MA, Gondim LF, Ellis JT (2013). What is the global economic impact of Neospora caninum in cattle–the billion dollar question. Int. J. Parasitol..

[11] Delany I, Rappuoli R, De Gregorio E (2014). Vaccines for the 21st century. EMBO Mol. Med..

[12] Sallusto F, Lanzavecchia A, Araki K, Ahmed R (2010). From vaccines to memory and back. Immunity.

[13] Kaech SM, Wherry EJ, Ahmed R (2002). Effector and memory T-cell differentiation: Implications for vaccine development. Nat. Rev. Immunol..

[14] McAllister MM (2014). Successful vaccines for naturally occurring protozoal diseases of animals should guide human vaccine research. A review of protozoal vaccines and their designs. Parasitology.

[15] O'Hagan DT, MacKichan ML, Singh M (2001). Recent developments in adjuvants for vaccines against infectious diseases. Biomol. Eng..

[16] Zhou B (2016). Reversion of cold-adapted live attenuated influenza vaccine into a pathogenic virus. J. Virol..

[17] Moyle PM, Toth I (2013). Modern subunit vaccines: Development, components, and research opportunities. ChemMedChem.

[18] Lee S, Nguyen MT (2015). Recent advances of vaccine adjuvants for infectious diseases. Immune Netw..

[19] Rathinasamy V, Poole WA, Bastos RG, Suarez CE, Cooke BM (2019). Babesiosis vaccines: Lessons learned, challenges ahead, and future glimpses. Trends Parasitol..

[20] Rappuoli R (2001). Reverse vaccinology, a genome-based approach to vaccine development. Vaccine.

[21] Goodswen SJ, Kennedy PJ, Ellis JT (2013). A guide to in silico vaccine discovery for eukaryotic pathogens. Brief. Bioinform..

[22] Goodswen SJ, Kennedy PJ, Ellis JT (2013). A novel strategy for classifying the output from an in silico vaccine discovery pipeline for eukaryotic pathogens using machine learning algorithms. Bmc Bioinform..

[23] Bowman BN (2011). Improving reverse vaccinology with a machine learning approach. Vaccine.

[24] Blythe MJ, Flower DR (2005). Benchmarking B cell epitope prediction: Underperformance of existing methods. Protein Sci..

[25] Deavin AJ, Auton TR, Greaney PJ (1996). Statistical comparison of established T-cell epitope predictors against a large database of human and murine antigens. Mol. Immunol..

[26] Wang P (2008). A systematic assessment of MHC class II peptide binding predictions and evaluation of a consensus approach. Plos Comput. Biol..

[27] Yang B, Sayers S, Xiang ZS, He YQ (2011). Protegen: A web-based protective antigen database and analysis system. Nucleic Acids Res..

[28] Webster RG, Laver WG (1966). Influenza virus subunit vaccines - immunogenicity and lack of toxicity for rabbits of ether- and detergent-disrupted virus. J. Immunol..

[29] Neumann, M., King,D., Beltagy, I., Ammar, W. ScispaCy: Fast and robust models for biomedical natural language processing. In *Proceedings of the 18th BioNLP Workshop and Shared Task*, pages 319–327, Florence, Italy. Association for Computational Linguistics. https://aclanthology.org/W19-5034.pdf (2019).

[30] Bateman A (2015). UniProt: A hub for protein information. Nucleic Acids Res..

[31] Li W, Godzik A (2006). Cd-hit: A fast program for clustering and comparing large sets of protein or nucleotide sequences. Bioinformatics.

[32] Flower DR, Macdonald IK, Ramakrishnan K, Davies MN, Doytchinova IA (2010). Computer aided selection of candidate vaccine antigens. Immunome Res..

[33] Goodswen SJ, Kennedy PJ, Ellis JT (2014). Vacceed: A high-throughput in silico vaccine candidate discovery pipeline for eukaryotic pathogens based on reverse vaccinology. Bioinformatics.

[34] Montoya JG, Liesenfeld O (2004). Toxoplasmosis. Lancet.

[35] Gohil S, Kats LM, Sturm A, Cooke BM (2010). Recent insights into alteration of red blood cells by Babesia bovis: Moovin' forward. Trends Parasitol..

[36] Suarez CE (2019). Unravelling the cellular and molecular pathogenesis of bovine babesiosis: Is the sky the limit?. Int. J. Parasitol..

[37] Beeson JG (2016). Merozoite surface proteins in red blood cell invasion, immunity and vaccines against malaria. FEMS Microbiol. Rev..

[38] Sheehy SH (2012). ChAd63-MVA-vectored blood-stage malaria vaccines targeting MSP1 and AMA1: Assessment of efficacy against mosquito bite challenge in humans. Mol. Ther..

[39] Sirima SB, Cousens S, Druilhe P (2011). Protection against malaria by MSP3 candidate vaccine. N. Engl. J. Med..

[40] Tinto H (2015). Efficacy and safety of RTS, S/AS01 malaria vaccine with or without a booster dose in infants and children in Africa: Final results of a phase 3, individually randomised, controlled trial. Lancet.

[41] Sirima SB (2016). A phase 2b randomized, controlled trial of the efficacy of the GMZ2 malaria vaccine in African children. Vaccine.

[42] Palacpac NMQ, Arisue N, Tougan T, Ishii KJ, Horii T (2011). Plasmodium falciparum serine repeat antigen 5 (SE36) as a malaria vaccine candidate. Vaccine.

[43] Healer J (2013). Vaccination with conserved regions of erythrocyte-binding antigens induces neutralizing antibodies against multiple strains of plasmodium falciparum. PLoS ONE.

[44] Arumugam TU (2011). Discovery of GAMA, a plasmodium falciparum merozoite micronemal protein, as a novel blood-stage vaccine candidate antigen. Infect. Immun..

[45] Ntege EH (2017). Blood-stage malaria vaccines: Post-genome strategies for the identification of novel vaccine candidates. Expert Rev. Vaccines.

[46] Daubersies P (2000). Protection against Plasmodium falciparum malaria in chimpanzees by immunization with the conserved preerythrocytic liver-stage antigen 3. Nat. Med..

[47] Pirahmadi S (2019). Cell-traversal protein for ookinetes and sporozoites (CelTOS) formulated with potent TLR adjuvants induces high-affinity antibodies that inhibit Plasmodium falciparum infection in Anopheles stephensi. Malar. J..

[48] John CC (2005). Correlation of high levels of antibodies to multiple pre-erythrocytic Plasmodium falciparum antigens and protection from infection. Am. J. Trop. Med. Hyg..

[49] Lopez C, Yepes-Perez Y, Diaz-Arevalo D, Patarroyo ME, Patarroyo MA (2018). The in vitro antigenicity of plasmodium vivax Rhoptry Neck Protein 2 (PvRON2) B- and T-epitopes selected by HLA-DRB1 binding profile. Front. Cell. Infect. Microbiol..

[50] Swearingen KE (2016). Interrogating the plasmodium sporozoite surface: Identification of surface-exposed proteins and demonstration of glycosylation on CSP and TRAP by mass spectrometry-based proteomics. Plos Pathog..

[51] Fidock DA (1997). Plasmodium falciparum sporozoite invasion is inhibited by naturally acquired or experimentally induced polyclonal antibodies to the STARP antigen. Eur. J. Immunol..

[52] Gilson PR (2006). Identification and stoichiometry of glycosylphosphatidylinositol-anchored membrane proteins of the human malaria parasite Plasmodium falciparum. Mol. Cell. Proteom..

[53] Ito D (2013). RALP1 Is a Rhoptry Neck erythrocyte-binding protein of plasmodium falciparum merozoites and a potential blood-stage vaccine candidate antigen. Infect. Immun..

[54] Douglas AD (2015). A PfRH5-based vaccine is efficacious against heterologous strain blood-stage plasmodium falciparum infection in aotus monkeys. Cell Host Microbe.

[55] Genton B (2002). A recombinant blood-stage malaria vaccine reduces Plasmodium falciparum density and exerts selective pressure on parasite populations in a phase 1–2b trial in Papua New Guinea. J. Infect. Dis..

[56] Maier AG, Cooke BM, Cowman AF, Tilley L (2009). Malaria parasite proteins that remodel the host erythrocyte. Nat. Rev. Microbiol..

[57] Ahmadpour E (2017). Enhancing immune responses to a DNA vaccine encoding Toxoplasma gondii GRA14 by calcium phosphate nanoparticles as an adjuvant. Immunol. Lett..

[58] Ching XT, Fong MY, Lau YL (2016). Evaluation of immunoprotection conferred by the subunit vaccines of GRA2 and GRA5 against acute toxoplasmosis in BALB/c mice. Front. Microbiol..

[59] Golkar M (2007). Evaluation of protective effect of recombinant dense granule antigens GRA2 and GRA6 formulated in monophosphoryl lipid A (MPL) adjuvant against Toxoplasma chronic infection in mice. Vaccine.

[60] Hiszczynska-Sawicka E (2011). Evaluation of immune responses in sheep induced by DNA immunization with genes encoding GRA1, GRA4, GRA6 and GRA7 antigens of Toxoplasma gondii. Vet. Parasitol..

[61] Quan JH (2012). Induction of protective immune responses by a multiantigenic DNA vaccine encoding GRA7 and ROP1 of toxoplasma gondii. Clin. Vaccine Immunol..

[62] Dautu G (2007). Toxoplasma gondii: DNA vaccination with genes encoding antigens MIC2, WAP, AMA1 and BAG1 and evaluation of their immunogenic potential. Exp. Parasitol..

[63] Dodangeh S (2021). Protective efficacy by a novel multi-epitope vaccine, including MIC3, ROP8, and SAG1, against acute Toxoplasma gondii infection in BALB/c mice. Microb. Pathog..

[64] Yuan Z-G (2011). Protective effect against toxoplasmosis in mice induced by DNA immunization with gene encoding Toxoplasma gondii ROP18. Vaccine.

[65] Doskaya M (2018). Discovery of new Toxoplasma gondii antigenic proteins using a high throughput protein microarray approach screening sera of murine model infected orally with oocysts and tissue cysts. Parasites Vectors.

[66] Lourenco EV (2006). Immunization with MIC1 and MIC4 induces protective immunity against Toxoplasma gondii. Microbes Infect..

[67] Hiszczynska-Sawicka E (2010). The immune responses of sheep after DNA immunization with, Toxoplasma gondii MAGI antigen-with and without co-expression of ovine interleukin 6. Vet. Immunol. Immunopathol..

[68] Sonaimuthu P, Ching XT, Fong MY, Kalyanasundaram R, Lau YL (2016). Induction of protective immunity against toxoplasmosis in BALB/c mice vaccinated with toxoplasma gondii Rhoptry-1. Front. Microbiol..

[69] Gao Q (2018). Immune response and protective effect against chronic Toxoplasma gondii infection induced by vaccination with a DNA vaccine encoding profilin. Bmc Infect. Dis..

[70] Antonio Alvarez J (2010). Immunization of Bos taurus Steers with Babesia bovis Recombinant Antigens MSA-1, MSA-2c and 12D3. Transbound. Emerg. Dis..

[71] Altangerel K (2012). Phylogenetic relationships of Mongolian Babesia bovis isolates based on the merozoite surface antigen (MSA)-1, MSA-2b, and MSA-2c genes. Vet. Parasitol..

[72] Gimenez AM (2016). A recombinant multi-antigen vaccine formulation containing Babesia bovis merozoite surface antigens MSA-2a(1), MSA-2b and MSA-2c elicits invasion-inhibitory antibodies and IFN-gamma producing cells. Parasites Vectors.

[73] Terkawi MA (2013). Molecular characterization of a new babesia bovis thrombospondin-related anonymous protein (BbTRAP2). PLoS ONE.

[74] Terkawi MA (2011). Secretion of a new spherical body protein of Babesia bovis into the cytoplasm of infected erythrocytes. Mol. Biochem. Parasitol..

[75] Norimine J (2003). Stimulation of T-helper cell gamma interferon and immunoglobulin G responses specific for Babesia bovis rhoptry-associated protein 1 (RAP-1) or a RAP-1 protein lacking the carboxy-terminal repeat region is insufficient to provide protective immunity against virulent B-bovis challenge. Infect. Immun..

[76] Gohil S (2013). Bioinformatic prediction of the exportome of Babesia bovis and identification of novel proteins in parasite-infected red blood cells. Int. J. Parasitol..

[77] Allred DR (2000). The ves multigene family of B-bovis encodes components of rapid antigenic variation at the infected erythrocyte surface. Mol. Cell.

[78] Brayton KA (2007). Genome sequence of babesia bovis and comparative analysis of apicomplexan hemoprotozoa. PLoS Pathog..

[79] Berna L (2021). Reevaluation of the Toxoplasma gondii and Neospora caninum genomes reveals misassembly, karyotype differences, and chromosomal rearrangements. Genome Res..

[80] Reid AJ (2012). Comparative genomics of the apicomplexan parasites toxoplasma gondii and neospora caninum: Coccidia differing in host range and transmission strategy. Plos Pathog..

[81] Miyamoto M (2014). Performance comparison of second- and third-generation sequencers using a bacterial genome with two chromosomes. Bmc Genom..

[82] Torresen OK (2019). Tandem repeats lead to sequence assembly errors and impose multi-level challenges for genome and protein databases. Nucleic Acids Res..

[83] Xiao T, Zhou W (2020). The third generation sequencing: The advanced approach to genetic diseases. Transl. Pediatr..

[84] Cock PJA (2009). Biopython: freely available Python tools for computational molecular biology and bioinformatics. Bioinformatics.

[85] Tiwari, B., Das, P., Das, A. K. A comparative study on various text classification methods. In *Computational Intelligence in Pattern Recognition: Proceedings of CIPR 2020* Vol. 1120 *Advances in Intelligent Systems and Computing* (eds A. K. Das *et al.*) 539–549. https://link-springercom.ezproxy.lib.uts.edu.au/book/10.1007/978-981-15-2449-3 (2020).

[86] Burges CJC (1998). A tutorial on Support Vector Machines for pattern recognition. Data Min. Knowl. Discov..

[87] Nalepa J, Kawulok M (2019). Selecting training sets for support vector machines: A review. Artif. Intell. Rev..

[88] Roldan-Valadez E, Salazar-Ruiz SY, Ibarra-Contreras R, Rios C (2019). Current concepts on bibliometrics: A brief review about impact factor, Eigenfactor score, CiteScore, SCImago journal rank, source-normalised impact per paper, H-index, and alternative metrics. Ir. J. Med. Sci..

[89] Ali MJ (2021). Understanding the 'g-index' and the 'e-index'. Semin. Ophthalmol..

[90] Egghe L (2006). Theory and practise of the g-index. Scientometrics.

[91] Kall L, Krogh A, Sonnhammer ELL (2004). A combined transmembrane topology and signal peptide prediction method. J. Mol. Biol..

[92] Krogh A, Larsson B, von Heijne G, Sonnhammer ELL (2001). Predicting transmembrane protein topology with a hidden Markov model: Application to complete genomes. J. Mol. Biol..

[93] Emanuelsson O, Brunak S, von Heijne G, Nielsen H (2007). Locating proteins in the cell using TargetP SignalP and related tools. Nat. Protoc..

[94] Armenteros JJA (2019). SignalP 5.0 improves signal peptide predictions using deep neural networks. Nat. Biotechnol..

[95] Pierleoni A, Martelli PL, Casadio R (2008). PredGPI: A GPI-anchor predictor. Bmc Bioinform..

[96] Peters B (2005). The immune epitope database and analysis resource: From vision to blueprint. PLoS Biol..

[97] Aurrecoechea C (2010). EuPathDB: A portal to eukaryotic pathogen databases. Nucleic Acids Res..

