# Supplementary material for: Compilation of parasitic immunogenic proteins from 30 years of published research using machine learning and natural language processing
Source: Sci Rep. 2022 Jun 20;12:10349. doi: 10.1038/s41598-022-13790-1 (PMC9208253; doi:10.1038/s41598-022-13790-1)

Supplementary Data S1

Stephen J. Goodswen, Paul J. Kennedy, John T. Ellis

Introduction

This document contains graphs showing the counts (or frequency) of words relating to animal models, diseases caused by parasites, or the genus of the disease causing parasites that are found in PubMed derived ‘title + abstract’ text from publications. The ‘title + abstract’ text is the output from the machine learning abstract classification stage of the pipeline. Given an initial input of 332,627 ‘title + abstract’ texts, 64,986 had a classification probability greater than or equal to 50% and were deemed ‘abstracts of interest’. The words in these ‘abstracts of interest’ were used for the word counts.

Number of publications from 1991 to 2021

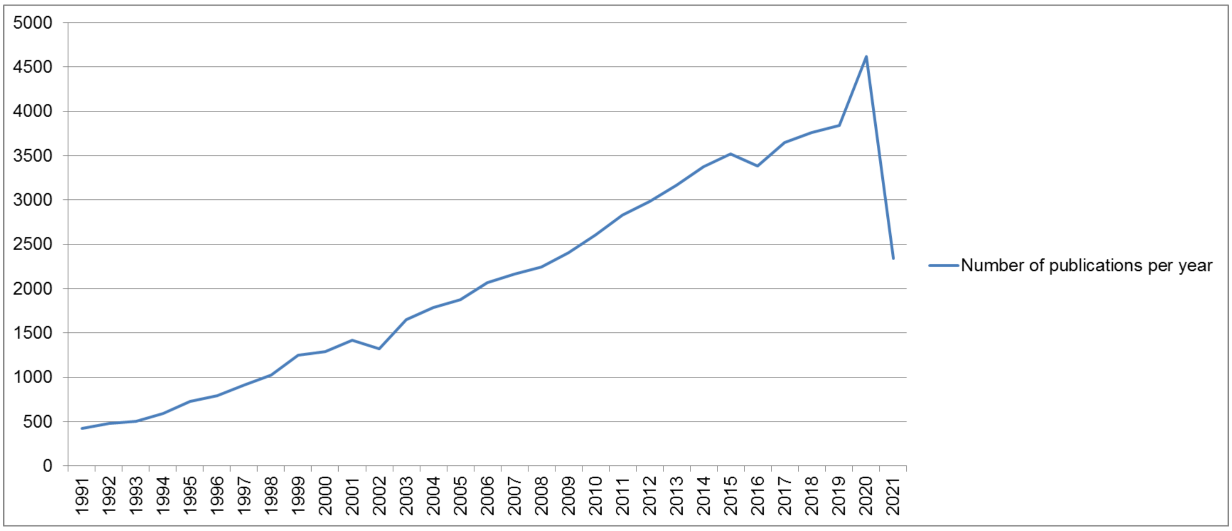

Animal models (1991 to 2021)

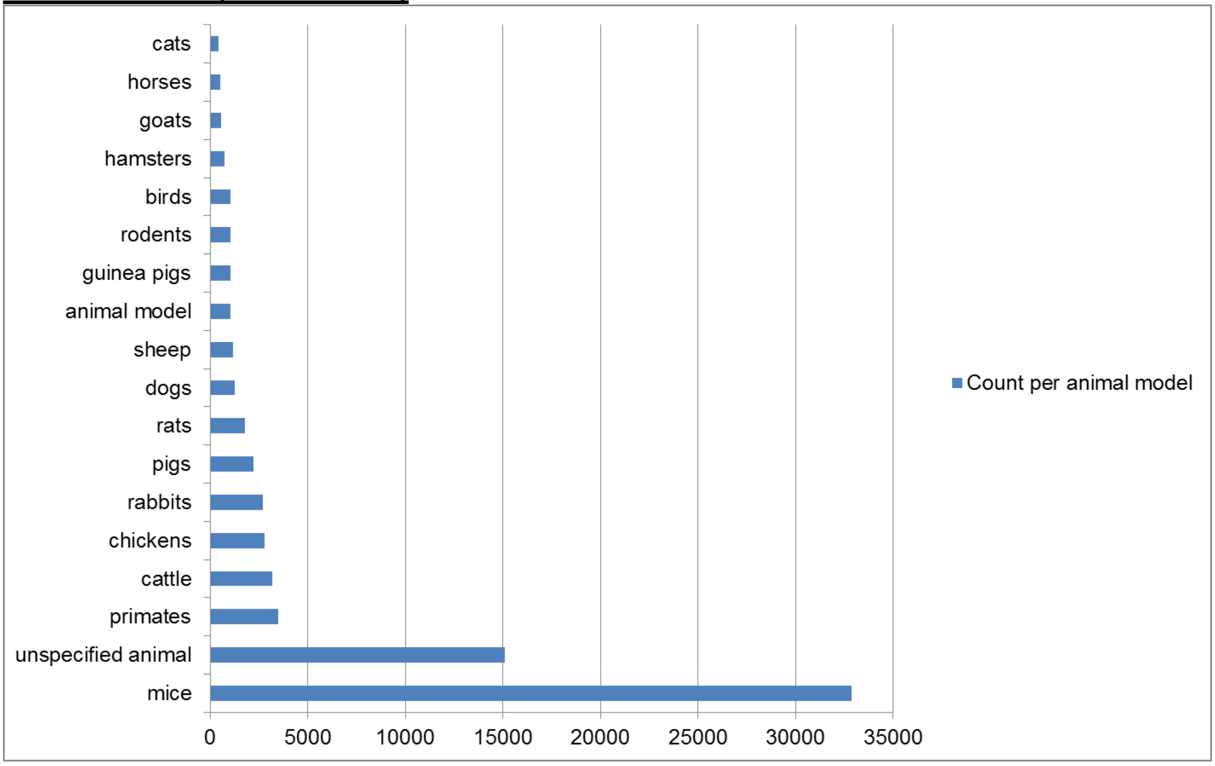

‘Animal model’ counts over three decades from 1991 to 2021

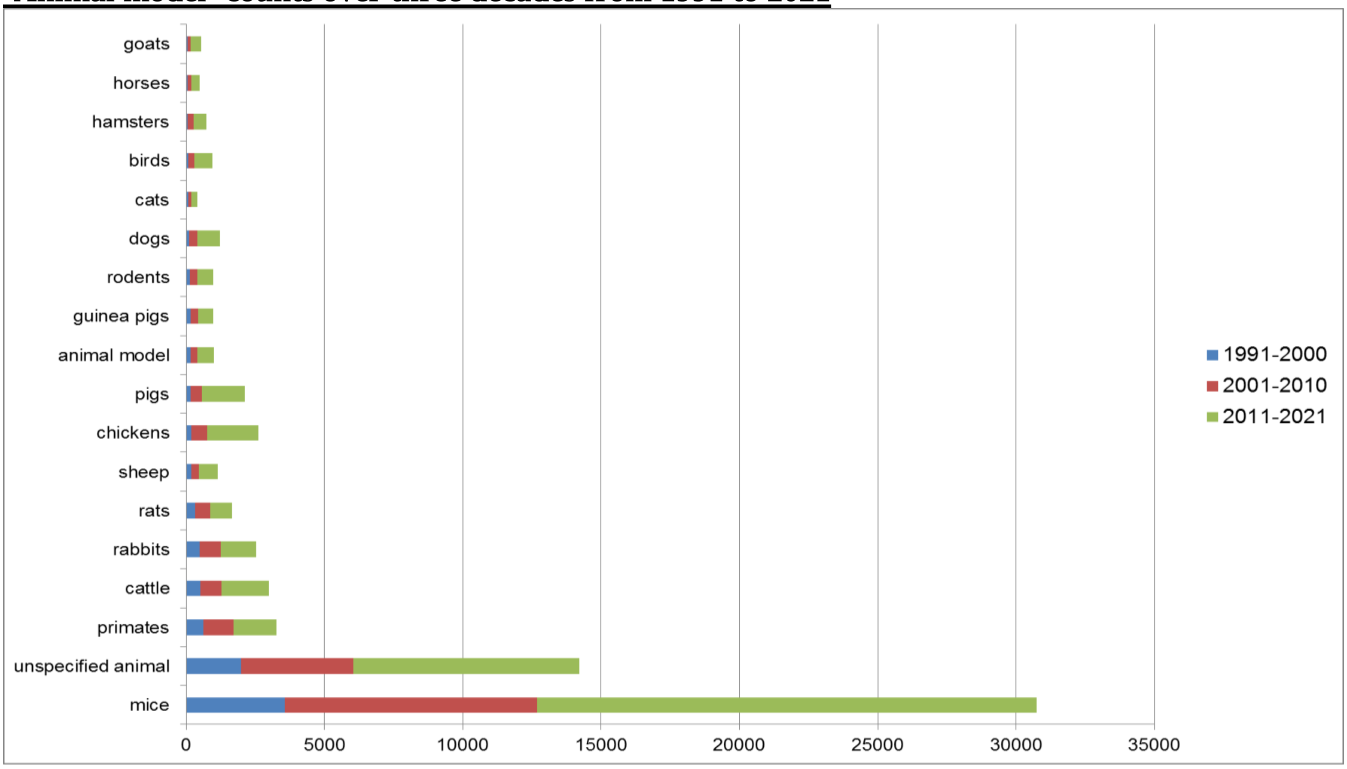

Diseases (1991 to 2021)

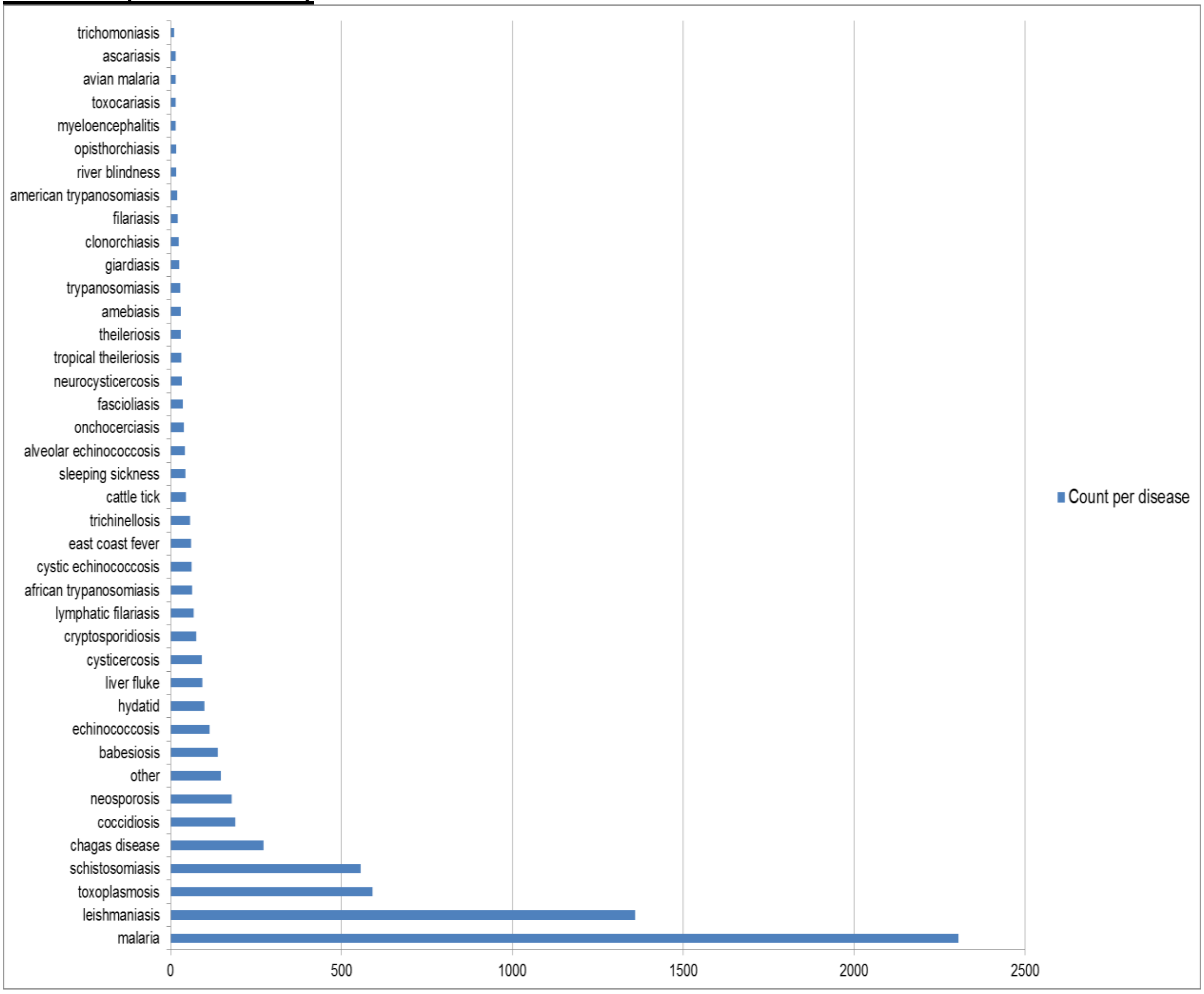

<u>Disease counts over three decades from 1991 to 2021</u>

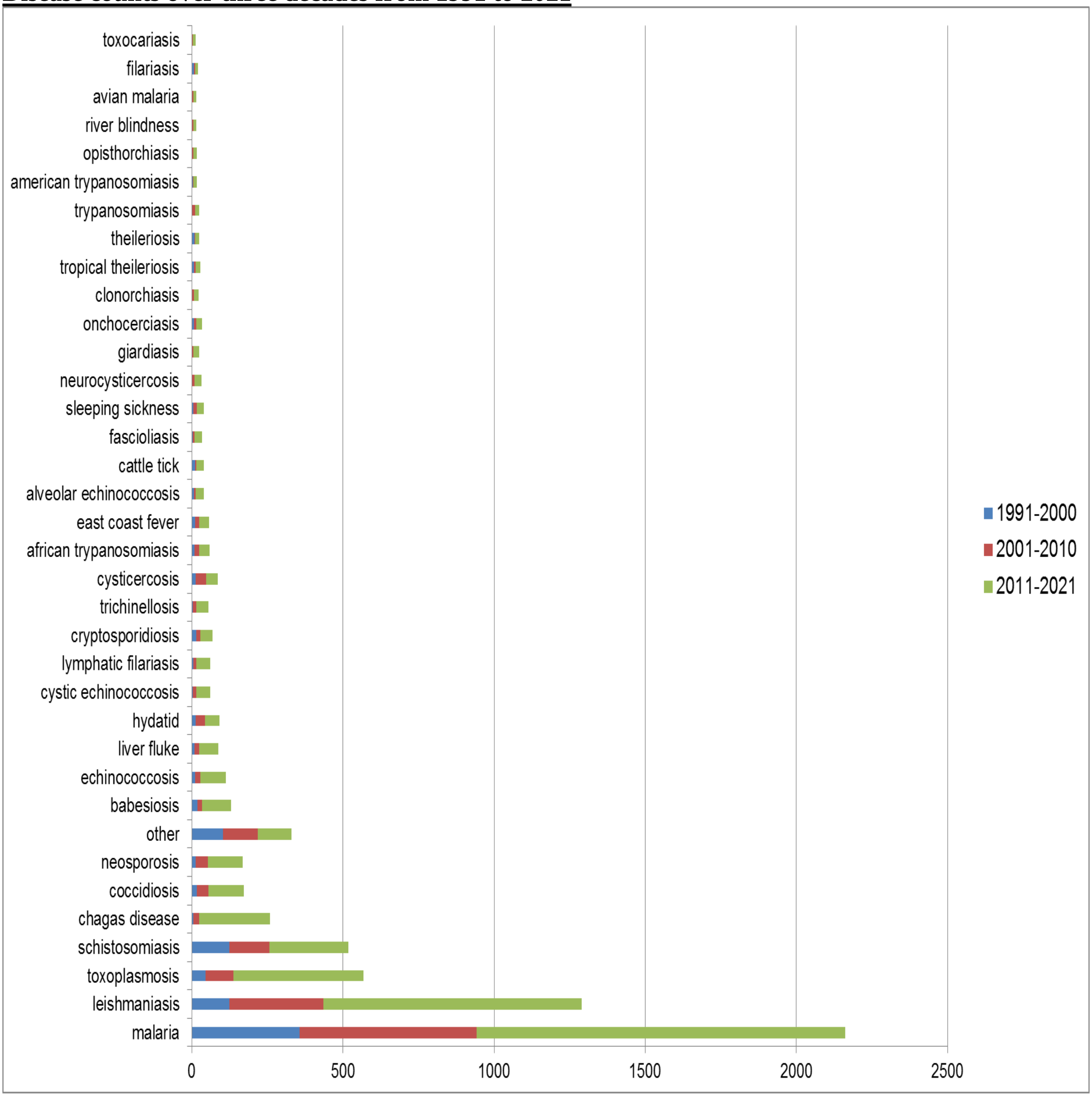

Genus (1991 to 2021)

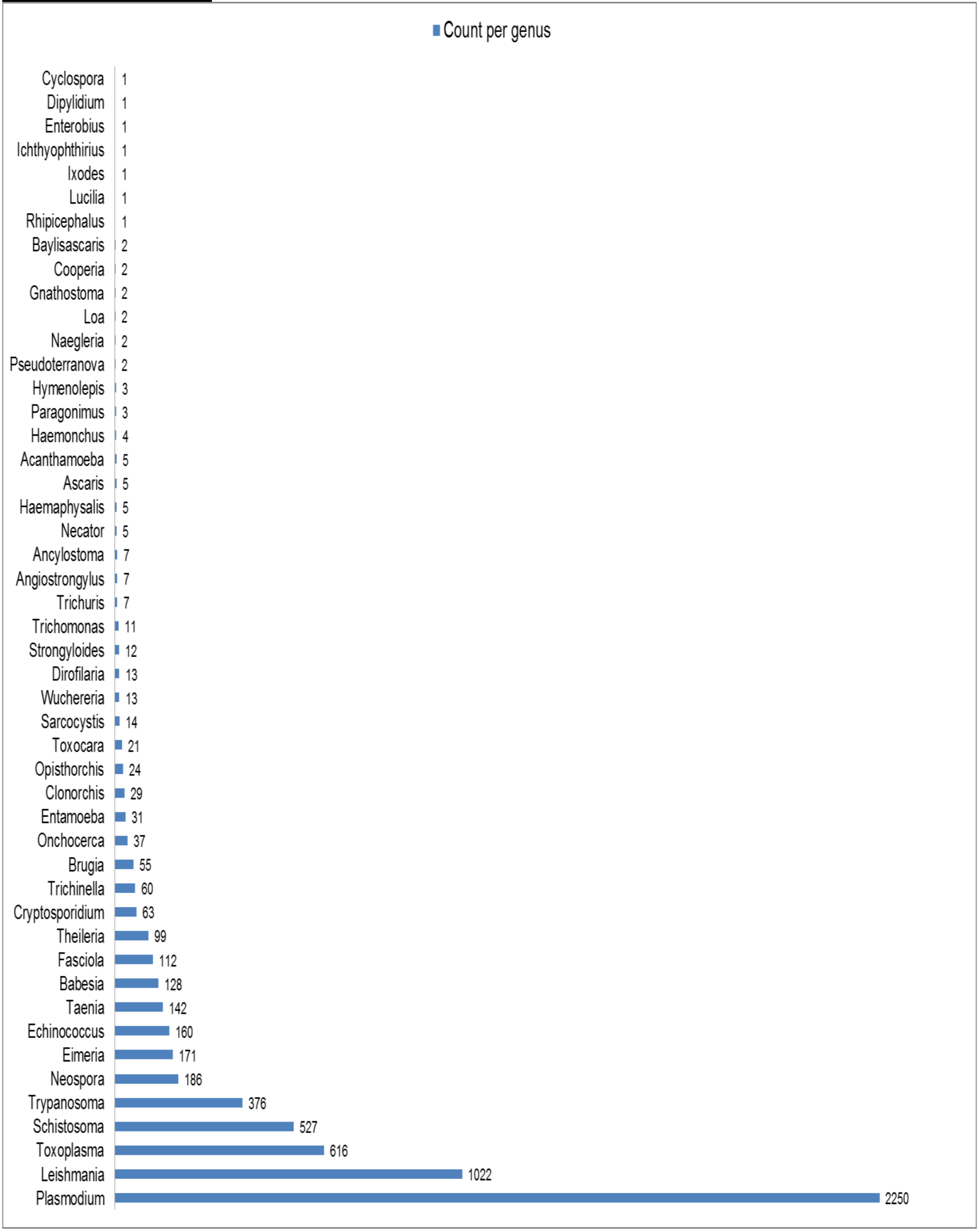

Supplement: Supplementary file 1 — Supplementary Information 1. [file 41598_2022_13790_MOESM1_ESM.pdf]
