# Supplementary material for: Compilation of parasitic immunogenic proteins from 30 years of published research using machine learning and natural language processing
Source: Sci Rep. 2022 Jun 20;12:10349. doi: 10.1038/s41598-022-13790-1 (PMC9208253; doi:10.1038/s41598-022-13790-1)
Supplement: Supplementary file 2 — Supplementary Information 2. [file 41598_2022_13790_MOESM2_ESM.pdf]

Supplementary Data S2

Stephen J. Goodswen, Paul J. Kennedy, John T. Ellis

Introduction

Two example abstracts were processed using the following spaCy named entity recognition (NER) models: web_sm and web_lg (see (https://spacy.io/models); and scispaCy NER models: sci_sm, sci_lg, and scibert (see https://allenai.github.io/scispacy/); and a custom NER model trained with protein and gene names. The aim was to identify in the abstracts named entities specific to the symbol or name of proteins and genes. The expected entities to identify are EG95 in Example #1, and TgPI-1, ROP2, GRA4, and serine protease inhibitor-1 in Example #2 (expected entities are highlighted in **bold**). Following each examples are the identified entities per NER model.

Example #1

Abstract:

In this study we investigated the efficacy of the **EG95** recombinant vaccine produced in Morocco by vaccination of sheep, including a combined vaccine incorporating **EG95** and clostridia antigens.Vaccination with **EG95** either as a monovalent vaccine or combined with clostridia antigens, protected sheep against a challenge infection with E. granulosus eggs and induced a strong, long lasting, and specific antibody response against the **EG95** antigen.

spaCy model = web_sm:

Morocco

spaCy model = web_lg:

Morocco

scispaCy model = sci_sm:

study
investigated
efficacy
EG95
recombinant vaccine
Morocco
vaccination
sheep
vaccine
EG95
clostridia antigens
Vaccination
EG95
monovalent
vaccine
clostridia antigens
sheep

E. granulosus
eggs
induced
strong
long lasting
antibody response
EG95 antigen

scispaCy model = sci_lg:

study
investigated
efficacy
EG95
recombinant vaccine
Morocco
vaccination
sheep
vaccine
EG95
clostridia antigens
Vaccination
EG95
monovalent
vaccine
clostridia antigens
sheep
infection
E. granulosus
eggs
induced
long lasting
antibody
response
EG95 antigen

***scispaCy model = scibert*:**

study
investigated
efficacy
EG95
recombinant vaccine
Morocco
vaccination
sheep
combined vaccine
EG95
clostridia antigens
Vaccination
EG95
monovalent vaccine
combined
clostridia antigens

sheep
challenge infection
E. granulosus
eggs
induced
strong
long lasting
specific
antibody
response
EG95 antigen

Custom NER model

EG95

Example #2

Abstract:

Vaccine potential of antigen cocktails composed of recombinant Toxoplasma gondii **TgPI-1**, **ROP2** and **GRA4** proteins against chronic toxoplasmosis in C3H mice. The development of an effective and safe vaccine to prevent Toxoplasma gondii infection is an important aim due to the great clinical and economic impact of this parasitosis. We have previously demonstrated that immunization with the **serine protease inhibitor-1** (**TgPI-1**) confers partial protection to C3H/HeN and C57BL/6 mice. In order to improve the level of protection, in this work, we combined this novel antigen with **ROP2** and/or **GRA4** recombinant proteins (rTgPI-1+rROP2, rTgPI-1+rGRA4, rTgPI-1+rROP2+rGRA4) to explore the best combination against chronic toxoplasmosis in C3H/HeN mice. All tested vaccine formulations, administered following a homologous prime-boost protocol that combines intradermal and intranasal routes, conferred partial protection as measured by the reduction of brain cyst burden following oral challenge with tissue cysts of Me49 T. gondii strain. The highest level of protection was achieved by the mixture of rTgPI-1 and rROP2 proteins with an average parasite burden reduction of 50% compared to the unvaccinated control group. The vaccine-induced protective effect was related to the elicitation of systemic cellular and humoral immune responses that included antigen-specific spleen cell proliferation, the release of Th1/Th2 cytokines, and the generation of antigen-specific antibodies in serum. Additionally, mucosal immune responses were also induced, characterized by secretion of antigen-specific IgA antibodies in intestinal lavages and specific mesenteric lymph node cell proliferation. Our results demonstrate that rTgPI-1+rROP2 antigens seem a promising mixture to be combined with other immunogenic proteins in a multiantigenic vaccine formulation against toxoplasmosis.

spaCy model = web_sm:

Toxoplasma
TgPI-1
ROP2
GRA4
Toxoplasma
TgPI-1
ROP2
GRA4

rTgPI-1+rROP2+rGRA4
rTgPI-1
50%

spaCy model = web_lg:

Toxoplasma
ROP2
GRA4
Toxoplasma
ROP2
GRA4
50%
Th1
IgA

scispaCy model = sci_sm:

Vaccine
potential
antigen cocktails
recombinant Toxoplasma gondii
TgPI-1
ROP2
GRA4
proteins
chronic toxoplasmosis
C3H
mice
development
effective
vaccine
Toxoplasma gondii
clinical
economic impact
parasitosis
immunization
serine protease inhibitor-1
TgPI-1
confers
protection
C3H/HeN
C57BL/6
mice
level
protection
antigen
ROP2
GRA4
recombinant proteins
rTgPI-1+rROP2
rTgPI-1+rGRA4
rTgPI-1+rROP2+rGRA4
combination
chronic toxoplasmosis
C3H/HeN mice
tested
vaccine
formulations

administered
homologous
prime-boost protocol
intradermal
intranasal routes
partial
protection
measured
reduction
brain cyst
burden
oral
tissue cysts
Me49 T.
strain
level
protection
rROP2 proteins
average
parasite
burden reduction
compared
unvaccinated control group
vaccine-induced
protective effect
elicitation
systemic cellular
humoral immune responses
antigen-specific spleen
cell proliferation
release
Th1/Th2
cytokines
generation
antigen-specific antibodies
serum
mucosal immune responses
induced
characterized
secretion
antigen-specific IgA antibodies
intestinal
lavages
mesenteric lymph node cell proliferation
results
rTgPI-1+rROP2 antigens
immunogenic proteins
multiantigenic vaccine formulation
toxoplasmosis

scispaCy model = sci_lg:

Vaccine
potential
antigen

cocktails
recombinant Toxoplasma gondii
TgPI-1
ROP2
GRA4
proteins
chronic toxoplasmosis
C3H mice
development
effective
vaccine
Toxoplasma gondii
infection
clinical
economic impact
parasitosis
immunization
serine protease inhibitor-1
TgPI-1
partial
protection
C3H/HeN
C57BL/6 mice
improve
level
protection
antigen
ROP2
GRA4
recombinant proteins
rTgPI-1+rROP2
rTgPI-1+rGRA4
chronic toxoplasmosis
C3H/HeN mice
vaccine
formulations
administered
homologous
prime-boost protocol
intradermal
intranasal
routes
partial
protection
reduction
brain cyst
burden
oral
tissue cysts
Me49 T. gondii
strain
level
protection
rTgPI-1
rROP2 proteins

average
parasite
burden
reduction
unvaccinated
control group
vaccine-induced
protective effect
elicitation
systemic
cellular
humoral immune responses
antigen-specific spleen cell
proliferation
release
Th1/Th2
cytokines
antigen-specific antibodies
serum
mucosal
immune responses
induced
characterized
secretion
antigen-specific
intestinal lavages
mesenteric lymph node cell proliferation
results
rTgPI-1+rROP2 antigens
immunogenic proteins
multiantigenic
vaccine
formulation
toxoplasmosis

scispaCy model = scibert:

Vaccine
potential
antigen cocktails
recombinant Toxoplasma gondii
TgPI-1
ROP2
GRA4 proteins
chronic toxoplasmosis
C3H
mice
development
effective
vaccine
prevent
Toxoplasma gondii infection
clinical
economic impact
parasitosis

immunization
serine protease inhibitor-1
TgPI-1
partial
protection
C3H/HeN
C57BL/6
mice
improve
level
protection
novel
antigen
ROP2
GRA4
recombinant proteins
rTgPI-1+rROP2
rTgPI-1+rGRA4
combination
chronic toxoplasmosis
C3H/HeN mice
tested
vaccine formulations
administered
homologous
intradermal
intranasal routes
partial
protection
measured
reduction
brain cyst
burden
oral
tissue cysts
Me49 T.
gondii strain
level
protection
mixture
rTgPI-1
rROP2 proteins
average
parasite
burden reduction
compared
unvaccinated control group
vaccine-induced
protective effect
elicitation
systemic cellular
humoral immune responses
antigen-specific spleen
cell proliferation
release

Th1/Th2
cytokines
generation
antigen-specific antibodies
serum
mucosal
induced
characterized
secretion
antigen-specific IgA antibodies
intestinal
lavages
specific
mesenteric lymph node cell proliferation
results
rTgPI-1+rROP2 antigens
immunogenic proteins
multiantigenic vaccine
toxoplasmosis

Custom NER model

ROP2
GRA4
serine protease inhibitor-1
Me49
