# Supplementary material for: Compilation of parasitic immunogenic proteins from 30 years of published research using machine learning and natural language processing
Source: Sci Rep. 2022 Jun 20;12:10349. doi: 10.1038/s41598-022-13790-1 (PMC9208253; doi:10.1038/s41598-022-13790-1)
Supplement: Supplementary file 3 — Supplementary Information 3. [file 41598_2022_13790_MOESM3_ESM.pdf]

Supplementary Data S3

Stephen J. Goodswen, Paul J. Kennedy, John T. Ellis

Introduction

The number and quality of potential vaccine candidates obtained by the application of the study's pipeline is governed by the selection criteria and thresholds used. Namely:

- Start year for publications
- End year for publications
- A threshold applied to a probability score that the abstract contains words related to a protective response in an animal model i.e. the probability output from the ML abstract classification.
- A threshold applied to the number of publications that contain a candidate protein
- A threshold applied to the number of animal models referred to in the abstract.

Note: study's pipeline = 'title + abstract' classification using ML → 'rule-based + custom NER' entity extraction → gene and protein database entity checking → candidate list

The following examples show the impact on the number of classified 'abstracts of interest' and the number of extracted protein names when changing thresholds. Note also the comments for each example.

Example #1

Input: 50 Verified abstracts

Probability threshold >= 0.0 Publication threshold >= 1 Animal model threshold >= 0
Year Start >= 1991 Year End <= 2021

Number of validation IDs: 50
Number of abstracts: 50
Number of 'abstracts of interest': **50**
Warning: NO animal model for 24349483
Warning: NO animal model for 15908397
Number of abstracts with protein: **42**
Number of protein candidate names: 95

Example #2

Input: 50 Verified abstracts

Probability threshold >= **0.7** Publication threshold >= 1 Animal model threshold >= **1**
Year Start >= 1991 Year End <= 2021

Number of validation IDs: 50
Number of abstracts: 50
Number of 'abstracts of interest': 49
Number of abstracts with protein: **40**
Number of protein candidate names: 88

<u>Example #3</u>

Input: 50 Verified abstracts

Probability threshold >= **0.9** Publication threshold >= 1 Animal model threshold >= **1**
Year Start >= 1991 Year End <= 2021

Number of validation IDs: 50
Number of abstracts: 50
Number of 'abstracts of interest': **44**
Number of abstracts with protein: **37**
Number of protein candidate names: **84**

Comments on Examples 1-3:

The key statistic above is the 'Number of abstracts with protein' i.e., how many abstracts were identified to contain a protein in the 'abstract of interest' (one that reports a protein(s) to have a protective response in an animal model). The first point to note from Example #1 is that the pipeline is not perfect even when using no threshold restrictions. This means that we can expect a percentage of false positives and negatives given any number of input abstracts. Example #2 and #3 show that the more stringent the selection criteria, the more false negatives and potentially less false positives (note that all the Evidence abstracts are expected to be all 'abstracts of interest' and contain vaccine candidates).

<u>Example #4</u>

Input: All publications over the last 30 years that contained either the word 'parasite', 'vaccine', 'vaccinated', or 'vaccination' in its title or abstract text (332,627 in total)

Probability threshold >= **0.5** Publication threshold >= 1 Animal model threshold >= 1
Year Start >= 1991 Year End <= 2021

Number of abstracts: 332627
Number of 'abstracts of interest': 64690
Number of abstracts with protein: 2761
Number of protein candidate names: **1598**

<u>Example #5</u>

Input: As per Example #4 (332,627 'title + abstracts')

Probability threshold >= **0.99** Publication threshold >= **3** Animal model threshold >= 1
Year Start >= 1900 Year End <= 2021

Number of abstracts: 332627
Number of 'abstracts of interest': 11275
Number of abstracts with protein: 1776
Number of protein candidate names: **403**

Comments on Examples 4-5:

The 'Number of abstracts with protein' is huge in Example #4 and too many to manually verify. Applying stricter threshold restrictions greatly reduces the numbers as shown in Example #5. Note that to obtain the 403 protein candidate names, the 'Publication threshold >= 3'. This means a protein candidate is selected only if it is reported in three or more abstracts. The assumption is that a candidate reported in many publications is more likely a true positive than one reported in only one or two. This assumption of course presents a dilemma because it potentially excludes a true and perhaps novel candidate.

<u>Example #6</u>

Input: 100 'positive' Verified abstracts

Probability threshold >= **0.9** Publication threshold >= 1 Animal model threshold >= 1
Year Start >= 1991 Year End <= 2021
Number of validation IDs: 100
Number of abstracts: 100
Number of 'abstracts of interest': **99**
Number of abstracts with protein: **96**
Number of protein candidate names: 401

<u>Example #7</u>

Input: 100 'negative' Verified abstracts

Probability threshold >= **0.9** Publication threshold >= 1 Animal model threshold >= 1
Year Start >= 1991 Year End <= 2021
Number of validation IDs: 100
Number of abstracts: 100
Number of 'abstracts of interest': **9**
Number of abstracts with protein: **9**
Number of protein candidate names: 21

Comments on Examples 6-7:

With respect to the 'Number of abstracts with protein', Example #6 and Example #7 show 4% false negatives and 9% false positives. These outcomes provide a crude guideline as to the potential false outcomes from the pipeline when applying a high probability threshold (note that in such a small test it is not feasible to increase the 'Publication threshold', however a larger threshold such as '3' is proposed when processing 1000s of abstracts).
