# Supplementary material for: Compilation of parasitic immunogenic proteins from 30 years of published research using machine learning and natural language processing
Source: Sci Rep. 2022 Jun 20;12:10349. doi: 10.1038/s41598-022-13790-1 (PMC9208253; doi:10.1038/s41598-022-13790-1)
Supplement: Supplementary file 4 — Supplementary Information 4. [file 41598_2022_13790_MOESM4_ESM.pdf]

Supplementary Data S4

Stephen J. Goodswen, Paul J. Kennedy, John T. Ellis

Introduction

This document contains comparative tables and graphs to highlight differences between vaccine candidate proteins with respect to the phylum and/or genus of the protein's source organism. These proteins were identified using the study's pipeline. Differences are highlighted through the comparison of particular protein characteristics such as their associated parasitic disease, subcellular location, presence of transmembrane domains, GPI-anchors, and signal peptides. The contents below provide the links to the characteristics compared.

Note that the total number of candidates compared here are **1099**, which are associated with **5 phyla**: **Apicomplexa, Arthropoda, Euglenozoa, Nematoda, and Platyhelminthes.**

Note: study's pipeline = 'title + abstract' classification using ML → 'rule-based + custom NER' entity extraction → gene and protein database entity checking → candidate list

Contents

Number and percentage of publications per phylum 2
Number and percentage of publications per phylum per genus 2
Number of candidates per phylum 3
Number of candidates per phylum per genus 3
Number of candidates per phylum per genus per species 4
Average protein lengths per genus 5
Number of candidates per phylum per parasitic disease 6
Apicomplexa candidates per parasitic disease 7
Nematoda candidates per parasitic disease 7
Platyhelminthes candidates per parasitic disease 7
Euglenozoa candidates per parasitic disease 8
Number of candidates per phylum per subcellular location 8
Apicomplexan candidates per subcellular location 9
Platyhelminthes candidates per subcellular location 10
Transmembrane domains per phylum per genus 11
Average percentage of transmembrane domains (TM) in Apicomplexan candidates per genus 12
Average percentage of transmembrane (TM) domains in Platyhelminthes candidates per genus 13
Signal peptides per phylum per genus 13
Average percentage of signal peptides (SPs) in Apicomplexan candidates per genus 14
Average percentage of signal peptides (SPs) in Platyhelminthes candidates per genus 14
Average cleavage site location of signal peptides per phylum per genus 14
Average cleavage site location of signal peptides for Apicomplexan candidates 15

Average cleavage site location of signal peptides for Platyhelminthes candidates 15
Number and percentage of GPI-anchored proteins predicted by *PredGPI* per phylum per genus 16
Percentage of GPI-anchored types predicted by *PredGPI* for Apicomplexa phylum 17
Number and percentage of predicted vaccine candidates by *Vacceed* per phylum per genus 18
Percentage of predicted vaccine candidates by *Vacceed* for Apicomplexa phylum 18
Percentage of predicted vaccine candidates by *Vacceed* for Platyhelminthes phylum 19
Published epitopes per phylum per genus 19
Number of candidates per phylum per GO biological process 20
Apicomplexan candidates per GO biological process 22
Platyhelminthes candidates per GO biological process 23
Number of candidates per phylum per GO cellular component 23
Apicomplexan candidates per GO cellular component 26
Platyhelminthes candidates per GO cellular component 27
Number of candidates per phylum per GO molecular function 27
Apicomplexan candidates per GO molecular function 32
Platyhelminthes candidates per GO molecular function 33

Number and percentage of publications per phylum

Apicomplexa	5217	81.4
Platyhelminthes	896	13.9
Euglenozoa	208	3.2
Nematoda	81	1.2
Arthropoda	4	0.1

Number and percentage of publications per phylum per genus

**Apicomplexa**			
	Plasmodium	4055	63.3
	Toxoplasma	709	11.07
	Babesia	185	2.89
	Theileria	101	1.58
	Neospora	96	1.5
	Eimeria	55	0.86
	Cryptosporidium	10	0.16
	Sarcocystis	6	0.09
**Platyhelminthes**			
	Schistosoma	375	5.85
	Echinococcus	291	4.54
	Fasciola	201	3.14
	Clonorchis	26	0.41
	Taenia	2	0.03
	Opisthorchis	1	0.02
**Euglenozoa**			

	Leishmania	184	2.87
	Trypanosoma	24	0.37
**Nematoda**			
	Brugia	33	0.52
	Trichinella	22	0.34
	Haemonchus	11	0.17
	Wuchereria	10	0.16
	Onchocerca	3	0.05
	Toxocara	1	0.02
	Strongyloides	1	0.02
**Arthropoda**			
	Haemaphysalis	4	0.06

Number of candidates per phylum

Apicomplexa	621
Platyhelminthes	355
Euglenozoa	69
Nematoda	50
Arthropoda	4

Number of candidates per phylum per genus

**Apicomplexa**		
	Plasmodium	320
	Toxoplasma	149
	Babesia	61
	Neospora	33
	Eimeria	32
	Theileria	18
	Sarcocystis	4
	Cryptosporidium	4
**Platyhelminthes**		
	Schistosoma	171
	Echinococcus	99
	Fasciola	68
	Clonorchis	15
	Taenia	1
	Opisthorchis	1
**Euglenozoa**		
	Leishmania	54
	Trypanosoma	15
**Nematoda**		
	Brugia	13
	Trichinella	12
	Wuchereria	10
	Haemonchus	10
	Onchocerca	3
	Toxocara	1
	Strongyloides	1
**Arthropoda**		
	Haemaphysalis	4

Number of candidates per phylum per genus per species

**Apicomplexa**	
Plasmodium	
Plasmodium falciparum	103
Plasmodium vivax	57
Plasmodium knowlesi	41
Plasmodium berghei	34
Plasmodium falciparum (isolate 3D7)	33
Plasmodium yoelii	20
Plasmodium chabaudi chabaudi	11
Plasmodium gallinaceum	4
Plasmodium berghei (strain Anka)	4
Plasmodium cynomolgi (strain B)	3
Plasmodium sp. gorilla clade G2	2
Plasmodium malariae	2
Plasmodium coatneyi	2
Plasmodium vivax (strain Salvador I)	1
Plasmodium relictum	1
Plasmodium knowlesi (strain H)	1
Plasmodium fragile	1
Toxoplasma	
Toxoplasma gondii (strain ATCC 50611 / Me49)	87
Toxoplasma gondii	62
Neospora	
Neospora caninum (strain Liverpool)	20
Neospora caninum (Coccidian parasite)	13
Babesia	
Babesia bovis	52
Babesia microti	4
Babesia bigemina	3
Babesia microti (strain RI)	2
Eimeria	
Eimeria tenella (Coccidian parasite)	21
Eimeria maxima (Coccidian parasite)	6
Eimeria acervulina (Coccidian parasite)	5
Theileria	
Theileria parva (East coast fever infection agent)	14
Theileria annulata	4
Cryptosporidium	
Cryptosporidium parvum	3
Cryptosporidium parvum (strain Iowa II)	1
Sarcocystis	
Sarcocystis neurona	4
**Platyhelminthes**	
Schistosoma	
Schistosoma japonicum (Blood fluke)	88
Schistosoma mansoni (Blood fluke)	82
Schistosoma haematobium (Blood fluke)	1
Echinococcus	
Echinococcus granulosus (Hydatid tapeworm)	60
Echinococcus multilocularis (Fox tapeworm)	39
Fasciola	
Fasciola hepatica (Liver fluke)	51
Fasciola gigantica (Giant liver fluke)	17

- *Clonorchis*
  - *Clonorchis sinensis (Chinese liver fluke)* 15
- *Opisthorchis*
  - *Opisthorchis viverrini* 1
- *Taenia*
  - *Taenia solium (Pork tapeworm)* 1

Euglenozoa

- *Leishmania*
  - *Leishmania infantum* 32
  - *Leishmania donovani* 22
- *Trypanosoma*
  - *Trypanosoma cruzi (strain CL Brener)* 9
  - *Trypanosoma brucei gambiense (strain MHOM/CI/86/DAL972)* 3
  - *Trypanosoma brucei brucei (strain 927/4 GUTat10.1)* 3

Nematoda

- *Brugia*
  - *Brugia malayi (Filarial nematode worm)* 13
- *Haemonchus*
  - *Haemonchus contortus (Barber pole worm)* 10
- *Trichinella*
  - *Trichinella spiralis (Trichina worm)* 12
- *Strongyloides*
  - *Strongyloides ratti (Parasitic roundworm)* 1
- *Onchocerca*
  - *Onchocerca volvulus* 3
- *Wuchereria*
  - *Wuchereria bancrofti* 10
- *Toxocara*
  - *Toxocara canis (Canine roundworm)* 1

Arthropoda

- *Haemaphysalis*
  - *Haemaphysalis longicornis (Bush tick)* 4

Average protein lengths per genus

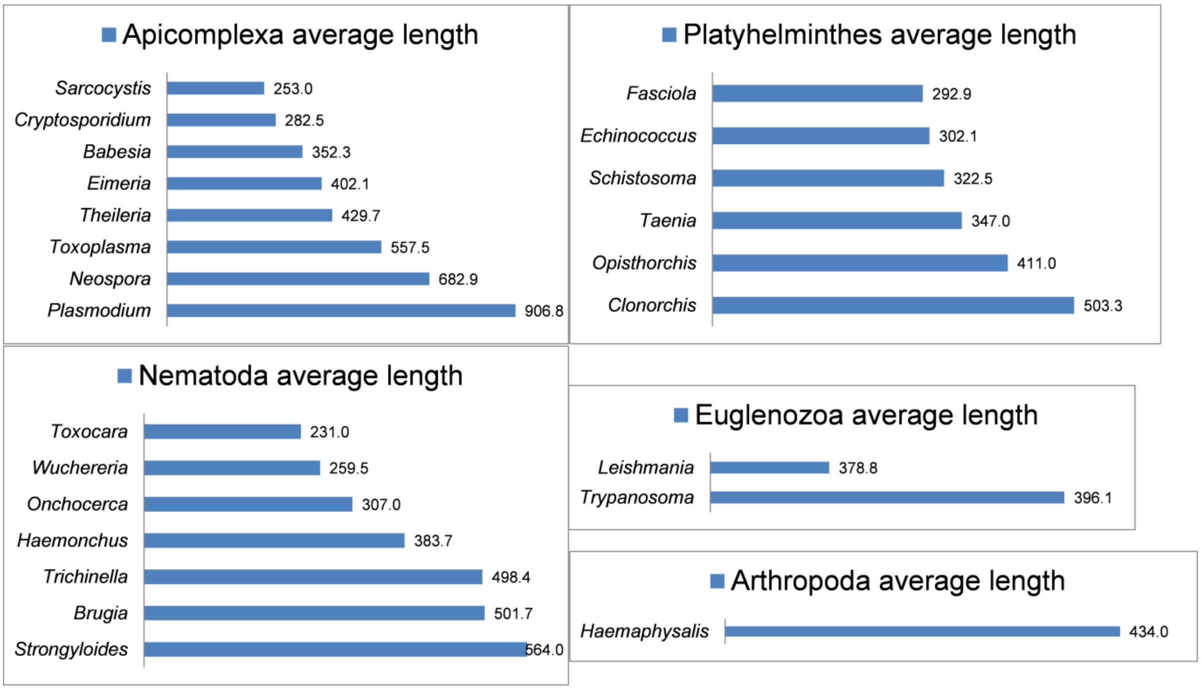

Number of candidates per phylum per parasitic disease

**Apicomplexa**		
	Malaria	320
	Toxoplasmosis	149
	Babesiosis	61
	Neosporosis	33
	Coccidiosis	32
	Theileriosis	18
	Sarcocystosis	4
	Cryptosporidiosis	4
**Platyhelminthes**		
	Schistosomiasis	171
	Fascioliasis	68
	Cystic echinococcosis	60
	Alveolar echinococcosis	39
	Clonorchiasis	15
	Cysticercosis	1
	Opisthorchiasis	1
**Euglenozoa**		
	Leishmaniasis	54
	Trypanosomiasis	15
**Nematoda**		
	Filariasis	23
	Trichinellosis	12
	Haemonchosis	10
	Onchocerciasis	3
	Toxocariasis	1
	Strongyloidiasis	1
**Arthropoda**		
	Bush tick	4

<u>Apicomplexa</u> candidates per <u>parasitic disease</u>

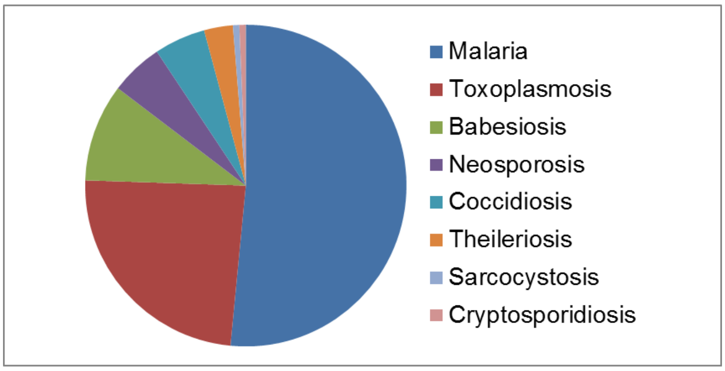

<u>Nematoda</u> candidates per <u>parasitic disease</u>

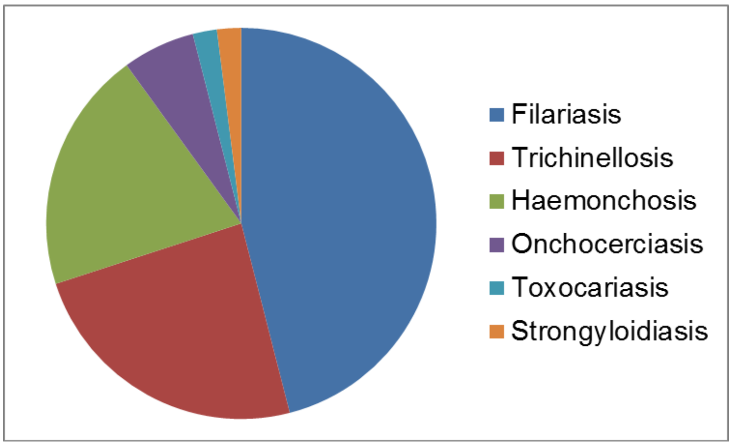

<u>Platyhelminthes</u> candidates per <u>parasitic disease</u>

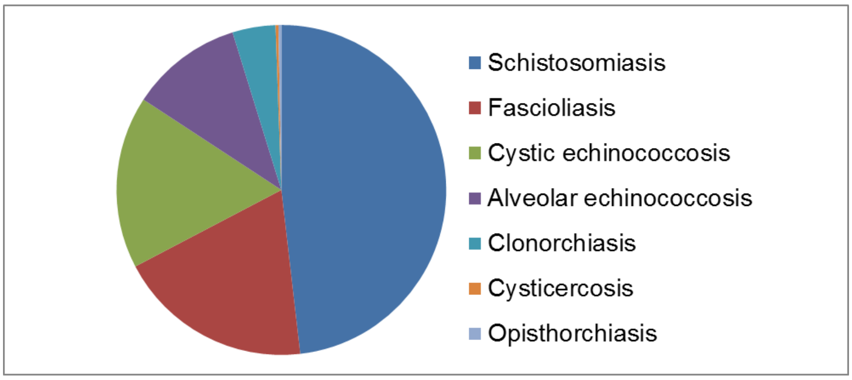

<u>Euglenozoa</u> candidates per <u>parasitic disease</u>

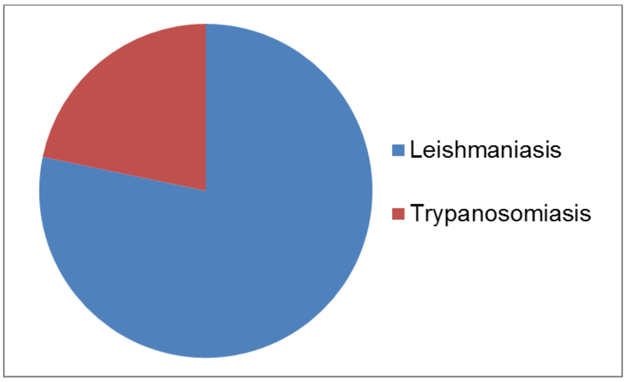

Number of candidates per <u>phylum</u> per <u>subcellular location</u>

Apicomplexa		
	unknown	521
	Cell membrane:Lipid-anchor:GPI-anchor:Membrane	35
	Membrane:Single-pass type I membrane protein	18
	Cell surface	8
	Cytoplasm:Nucleus	6
	Membrane:Multi-pass membrane protein	5
	Nucleus	3
	Membrane:Multi-pass membrane protein:Mitochondrion inner membrane	2
	Endoplasmic reticulum lumen	2
	Cytoplasm:cytoskeleton	2
	Cytoplasm	2
	Parasitophorous vacuole membrane	1
	Note=Erythrocyte cytoplasm of schizonts:Released into the culture supernatant at the time of schizont rupture:Only a minor fraction of GBP was found to be loosely associated with merozoites (By similarity)	1
	Mitochondrion inner membrane	1
	Cell surface:Cell membrane:Lipid-anchor:GPI-anchor:Note=Specifically present on the surface of female gametocytes	1
	Cell surface:Cell membrane:Lipid-anchor:GPI-anchor:Note=Present on the surface of merozoite	1
	Cell surface:Cell membrane	1
	Cell membrane:Lipid-anchor:GPI-anchor:Note=In microneme/rhoptry complexes	1
Nematoda		
	unknown	46
	Nucleus	2
	Endoplasmic reticulum lumen	1
	Cytoplasm:myofibril:Note=Thick filaments of the myofibrils	1
Platyhelminthes		
	unknown	240
	Membrane:Multi-pass membrane protein	69
	Cytoplasm:cytoskeleton	27
	Cytoplasm:myofibril	6

	Cytoplasm:Nucleus	5
	Cytoplasm	2
	Nucleus inner membrane:Multi-pass membrane protein:Nucleoplasmic side	1
	Lysosome	1
	Endoplasmic reticulum membrane:Multi-pass membrane protein:Membrane	1
	Endoplasmic reticulum lumen	1
	Cytoplasm:perinuclear region	1
	Cell membrane:Multi-pass membrane protein	1
Euglenozoa		
	unknown	53
	Cell membrane:Lipid-anchor:GPI-anchor:Membrane	5
	Mitochondrion inner membrane	2
	Endoplasmic reticulum lumen	2
	Cytoplasm	2
	Chromosome:Nucleus	2
	Nucleus	1
	Endoplasmic reticulum membrane:Multi-pass membrane protein:Membrane	1
	Cytoplasm:cytoskeleton	1
Arthropoda		
	unknown	3
	Endoplasmic reticulum lumen	1

Apicomplexan candidates per subcellular location

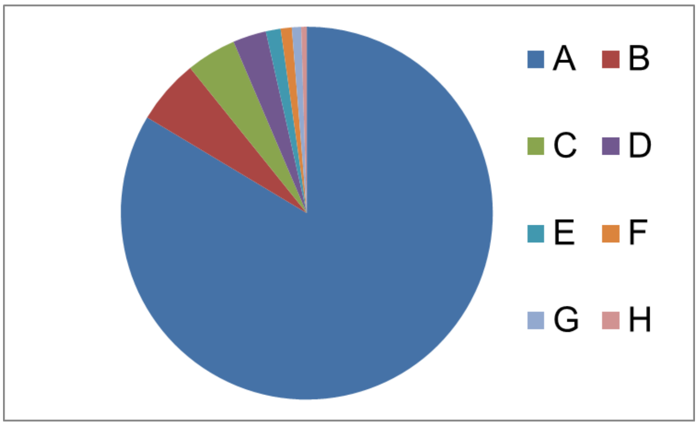

A	519	Unknown
B	35	Cell membrane:Lipid-anchor:GPI-anchor:Membrane
C	27	Other
D	18	Membrane:Single-pass type I membrane protein
E	8	Cell surface
F	6	Cytoplasm:Nucleus
G	5	Membrane:Multi-pass membrane protein
H	3	Nucleus

Platyhelminthes candidates per subcellular location

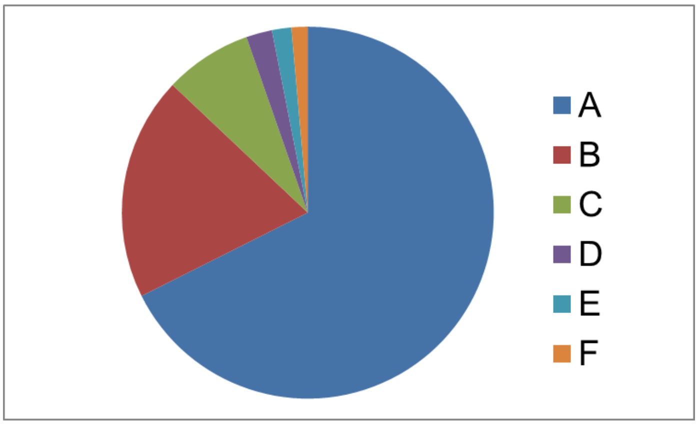

A	240	Unknown
B	69	Membrane:Multi-pass membrane protein
C	27	Cytoplasm:cytoskeleton
D	8	Other
E	6	Cytoplasm:myofibril
F	5	Cytoplasm:Nucleus

<u>Transmembrane domains</u> per <u>phylum</u> per <u>genus</u>

			TMHMM				Phobius				UniProt			
		No. of Candidates	with TMs	%	Total TMs	Avg. TMs	with TMs	%	Total TMs	Avg. TMs	with TMs	%	Total TMs	Avg. TMs
**Apicomplexa**														
	Plasmodium	320	173	54.1	250	1.5	223	69.7	314	1.4	157	49.1	223	1.4
	Toxoplasma	149	44	29.5	78	1.8	43	28.9	88	2.1	29	19.5	56	1.9
	Theileria	18	18	100.0	35	1.9	10	55.6	28	2.8	9	50.0	26	2.9
	Neospora	33	10	30.3	14	1.4	13	39.4	17	1.3	7	21.2	9	1.3
	Babesia	61	8	13.1	13	1.6	14	23.0	16	1.1	6	9.8	8	1.3
	Eimeria	32	3	9.4	3	1.0	2	6.2	2	1.0	2	6.2	2	1.0
	Sarcocystis	4	1	25.0	1	1.0	1	25.0	1	1.0	1	25.0	1	1.0
	Cryptosporidium	4	1	25.0	1	1.0	0	0.0	0	0.0	0	0.0	0	0.0
**Nematoda**														
	Trichinella	12	3	25.0	8	2.7	5	41.7	9	1.8	2	16.7	6	3.0
	Haemonchus	10	3	30.0	3	1.0	3	23.1	4	1.3	3	30.0	3	1.0
	Brugia	13	2	15.4	3	1.5	3	30.0	3	1.0	2	15.4	3	1.5
	Wuchereria	10	2	20.0	2	1.0	1	10.0	1	1.0	1	10.0	1	1.0
	Toxocara	1	0	0.0	0	0.0	0	0.0	0	0.0	0	0.0	0	0.0
	Strongyloides	1	0	0.0	0	0.0	0	0.0	0	0.0	0	0.0	0	0.0
	Onchocerca	3	0	0.0	0	0.0	0	0.0	0	0.0	0	0.0	0	0.0
**Platyhelminthes**														
	Schistosoma	171	56	32.7	226	4.0	55	32.2	237	4.3	53	31.0	216	4.1
	Echinococcus	99	39	39.4	131	3.4	38	38.4	130	3.4	37	37.4	126	3.4
	Fasciola	68	5	7.4	5	1.0	5	7.4	6	1.2	3	4.4	3	1.0
	Taenia	1	0	0.0	0	0.0	1	6.7	3	3.0	0	0.0	0	0.0
	Opisthorchis	1	0	0.0	0	0.0	0	0.0	0	0.0	0	0.0	0	0.0
	Clonorchis	15	0	0.0	0	0.0	0	0.0	0	0.0	0	0.0	0	0.0

**Euglenozoa**														
	Leishmania	54	21	38.9	32	1.5	8	14.8	17	2.1	7	13.0	15	2.1
	Trypanosoma	15	1	6.7	1	1.0	1	6.7	1	1.0	1	6.7	1	1.0
**Arthropoda**														
	Haemaphysalis	4	0	0.0	0	0.0	0	0.0	0	0.0	0	0.0	0	0.0

Average percentage of transmembrane (TM) domains in Apicomplexan candidates per genus

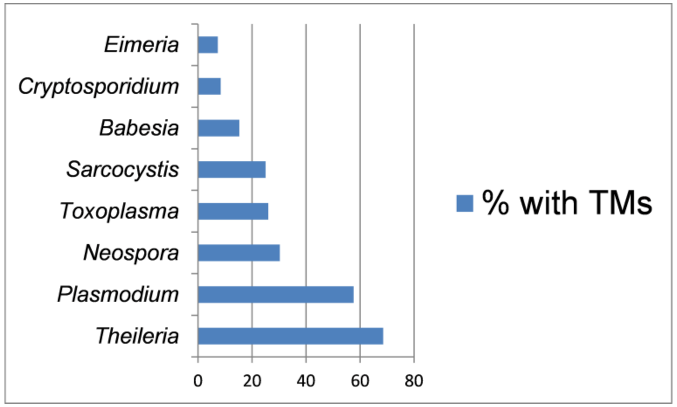

Average percentage of transmembrane (TM) domains in Platyhelminthes candidates per genus

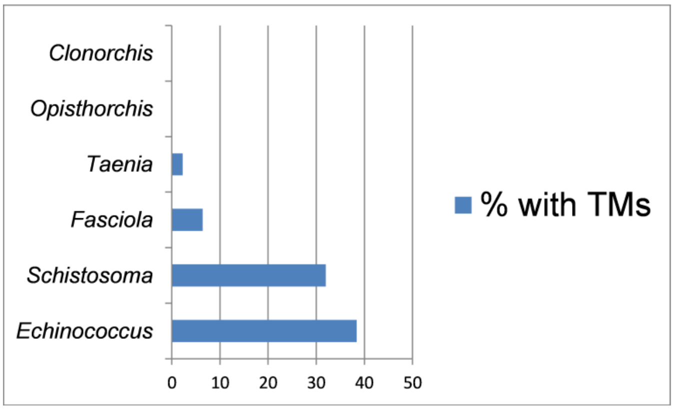

Signal peptides per phylum per genus

			TargetP		SignalP		Phobius	
		No. of Candidates	with SPs	%	with SPs	%	with SPs	%
**Apicomplexa**								
	Plasmodium	320	207	64.7	204	63.7	200	62.5
	Babesia	61	54	88.5	53	86.9	59	39.6
	Toxoplasma	149	52	34.9	49	32.9	55	90.2
	Theileria	18	16	88.9	16	88.9	16	88.9
	Neospora	33	15	45.5	10	30.3	14	42.4
	Eimeria	32	12	37.5	11	34.4	14	43.8
	Sarcocystis	4	4	100.0	3	75.0	4	100.0
	Cryptosporidium	4	1	25.0	1	25.0	2	50.0
**Nematoda**								
	Trichinella	12	7	58.3	7	58.3	5	41.7
	Haemonchus	10	3	30.0	3	30.0	3	30.0
	Brugia	13	2	15.4	2	15.4	3	23.1
	Wuchereria	10	1	10.0	1	10.0	1	10.0
	Toxocara	1	0	0.0	0	0.0	0	0.0
	Onchocerca	3	0	0.0	0	0.0	0	0.0
	Strongyloides	1	0	0.0	0	0.0	0	0.0
**Platyhelminthes**								
	Fasciola	68	20	29.4	19	27.9	18	26.5
	Schistosoma	171	15	8.8	15	8.8	16	9.4
	Echinococcus	99	14	14.1	12	12.1	15	15.2
	Clonorchis	15	0	0.0	0	0.0	0	0.0
	Taenia	1	0	0.0	0	0.0	0	0.0
	Opisthorchis	1	0	0.0	0	0.0	0	0.0
**Euglenozoa**								
	Leishmania	54	13	24.1	13	24.1	20	37.0
	Trypanosoma	15	5	33.3	4	26.7	5	33.3
**Arthropoda**								
	Haemaphysalis	4	1	25.0	1	25.0	1	25.0

Average percentage of signal peptides (SPs) in Apicomplexan candidates per genus

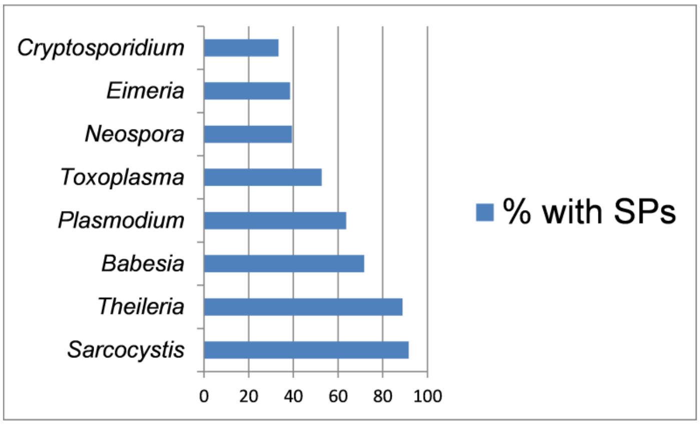

Average percentage of signal peptides (SPs) in Platyhelminthes candidates per genus

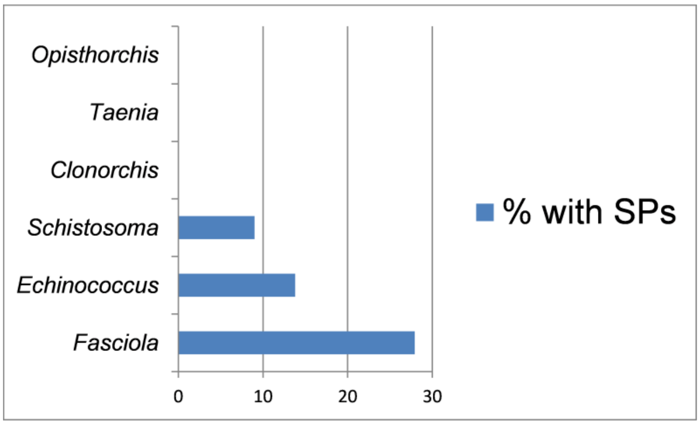

Average cleavage site location of signal peptides per phylum per genus

		Location (AAs)
**Apicomplexa**		
	Plasmodium	22.0
	Toxoplasma	26.2
	Babesia	21.1
	Theileria	20.6
	Neospora	27.0
	Eimeria	21.2
	Sarcocystis	24.3
	Cryptosporidium	22.0
**Nematoda**		
	Trichinella	20.0
	Haemonchus	16.7
	Brugia	21.0
	Wuchereria	23.0

	Toxocara	0.0
	Strongyloides	0.0
	Onchocerca	0.0
**Platyhelminthes**		
	Fasciola	19.2
	Schistosoma	21.1
	Echinococcus	18.9
	Taenia	0.0
	Opisthorchis	0.0
	Clonorchis	0.0
**Euglenozoa**		
	Leishmania	25.8
	Trypanosoma	23.8
**Arthropoda**		
	Haemaphysalis	16.0

Average cleavage site location of signal peptides for Apicomplexan candidates

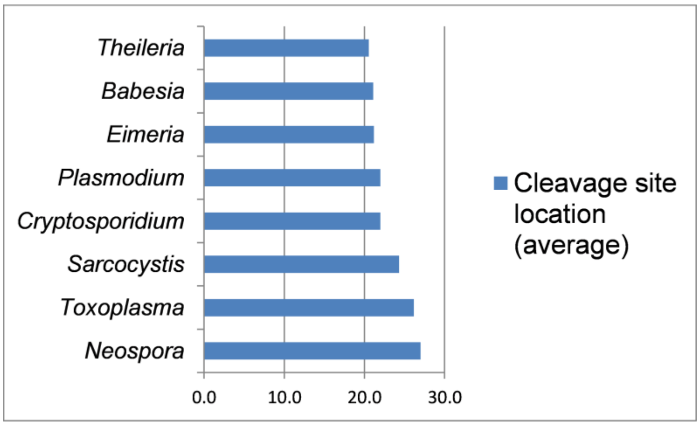

Average cleavage site location of signal peptides for Platyhelminthes candidates

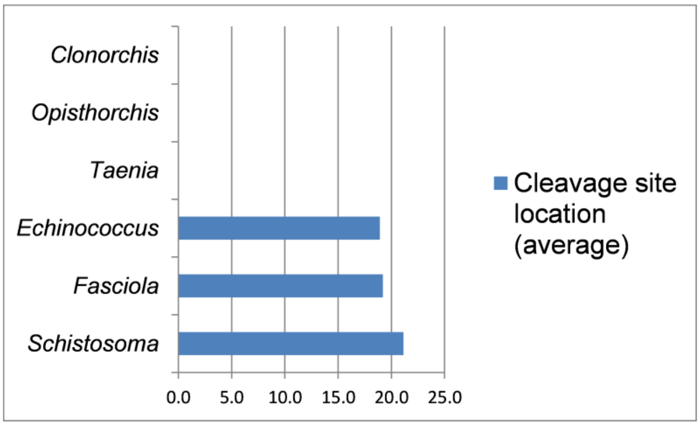

Number and percentage of GPI-anchored proteins predicted by *PredGPI* per phylum per genus

		No. of candidates	Not GPI-anchored	% not GPI-anchored	Weakly	%weakly	Probable	%probable	Highly	%highly
**Apicomplexa**										
	Plasmodium	320	200	62.5	4	1.2	40	12.5	76	23.8
	Toxoplasma	149	133	89.3	2	1.3	10	6.7	4	2.7
	Eimeria	32	29	90.6	0	0.0	2	6.2	1	3.1
	Neospora	33	25	75.8	3	9.1	5	15.2	0	0.0
	Babesia	61	17	27.9	0	0.0	12	19.7	32	52.5
	Theileria	18	16	88.9	1	5.6	0	0.0	1	5.6
	Cryptosporidium	4	4	100.0	0	0.0	0	0.0	0	0.0
	Sarcocystis	4	0	0.0	0	0.0	3	75.0	1	25.0
**Nematoda**										
	Trichinella	12	12	100.0	0	0.0	0	0.0	0	0.0
	Brugia	13	12	92.3	0	0.0	0	0.0	1	7.7
	Wuchereria	10	10	100.0	0	0.0	0	0.0	0	0.0
	Haemonchus	10	10	100.0	0	0.0	0	0.0	0	0.0
	Onchocerca	3	3	100.0	0	0.0	0	0.0	0	0.0
	Toxocara	1	1	100.0	0	0.0	0	0.0	0	0.0
	Strongyloides	1	1	100.0	0	0.0	0	0.0	0	0.0
**Platyhelminthes**										
	Schistosoma	171	166	97.1	1	0.6	4	2.3	0	0.0
	Echinococcus	99	87	87.9	5	5.1	7	7.1	0	0.0
	Fasciola	68	67	98.5	1	1.5	0	0.0	0	0.0
	Clonorchis	15	15	100.0	0	0.0	0	0.0	0	0.0
	Taenia	1	1	100.0	0	0.0	0	0.0	0	0.0
	Opisthorchis	1	1	100.0	0	0.0	0	0.0	0	0.0
**Euglenozoa**										
	Leishmania	54	50	92.6	1	1.9	1	1.9	2	3.7

	Trypanosoma	15	12	80.0	1	6.7	1	6.7	1	6.7
**Arthropoda**										
	Haemaphysalis	4	4	100.0	0	0.0	0	0.0	0	0.0

Percentage of <u>GPI-anchored</u> types predicted by <u>*PredGPI*</u> for <u>Apicomplexa phylum</u>

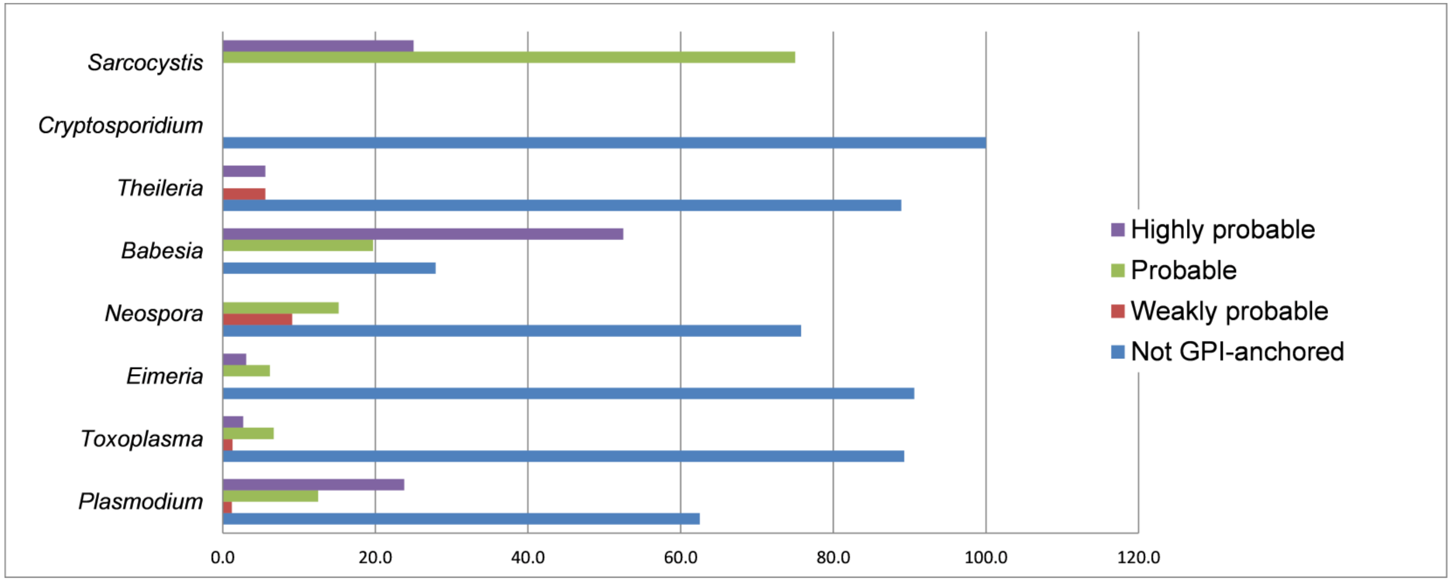

Number and percentage of predicted vaccine candidates by *Vacceed* per phylum per genus

		No. of Candidates	No. Predicted	% Predicted
**Apicomplexa**				
	Plasmodium	320	257	80.3
	Toxoplasma	149	79	53.0
	Babesia	61	58	95.1
	Neospora	33	20	60.6
	Theileria	18	18	100.0
	Eimeria	32	12	37.5
	Sarcocystis	4	4	100.0
	Cryptosporidium	4	1	25.0
**Nematoda**				
	Trichinella	12	10	83.3
	Haemonchus	10	6	60.0
	Brugia	13	5	38.5
	Wuchereria	10	2	20.0
	Onchocerca	3	0	0.0
	Toxocara	1	0	0.0
	Strongyloides	1	0	0.0
**Platyhelminthes**				
	Schistosoma	171	67	39.2
	Echinococcus	99	47	47.5
	Fasciola	68	23	33.8
	Clonorchis	15	1	6.7
	Taenia	1	0	0.0
	Opisthorchis	1	0	0.0
**Euglenozoa**				
	Leishmania	54	22	40.7
	Trypanosoma	15	6	40.0
**Arthropoda**				
	Haemaphysalis	4	1	25.0

Percentage of predicted vaccine candidates by *Vacceed* for Apicomplexa phylum

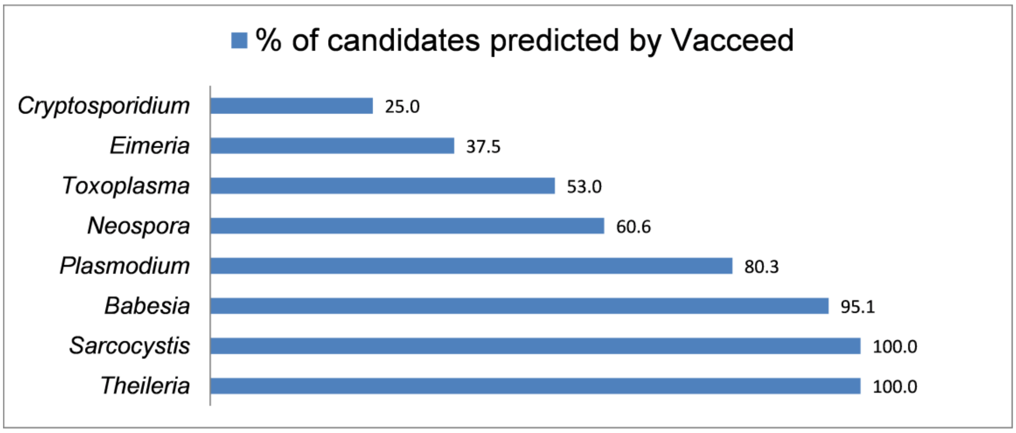

Percentage of predicted vaccine candidates by *Vacceed* for Platyhelminthes phylum

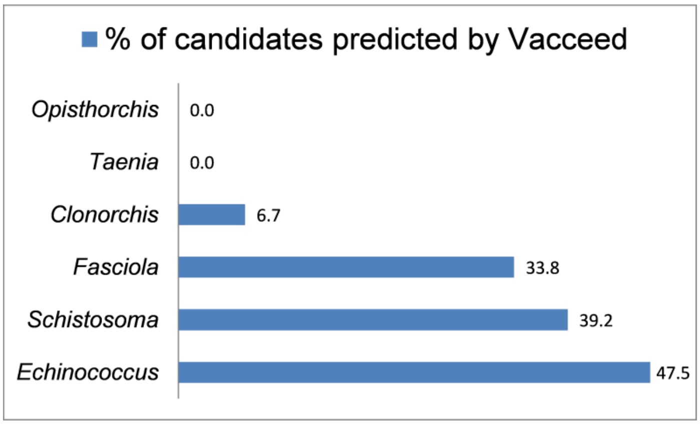

Published epitopes per phylum per genus

		No. of Candidates	with Epitopes	%	Total No. epitopes	Avg. No.
**Apicomplexa**						
	Plasmodium	320	32	10.0	382	11.9
	Toxoplasma	149	14	9.4	14	1.0
	Babesia	61	2	3.3	14	7.0
	Theileria	18	0	0.0	0	0.0
	Sarcocystis	4	0	0.0	0	0.0
	Neospora	33	0	0.0	0	0.0
	Eimeria	32	0	0.0	0	0.0
	Cryptosporidium	4	0	0.0	0	0.0
**Nematoda**						
	Onchocerca	3	1	33.3	11	11.0
	Wuchereria	10	1	10.0	1	1.0
	Brugia	13	1	7.7	1	1.0
	Trichinella	12	0	0.0	0	0.0
	Toxocara	1	0	0.0	0	0.0
	Strongyloides	1	0	0.0	0	0.0
	Haemonchus	10	0	0.0	0	0.0
Platyhelminthes						
	Schistosoma	171	4	2.3	28	7.0
	Echinococcus	99	2	2.0	2	1.0
	Fasciola	68	1	1.5	1	1.0
	Taenia	1	0	0.0	0	0.0
	Opisthorchis	1	0	0.0	0	0.0
	Clonorchis	15	0	0.0	0	0.0
**Euglenozoa**						
	Leishmania	54	9	16.7	20	2.2
	Trypanosoma	15	0	0.0	0	0.0
**Arthropoda**						
	Haemaphysalis	4	0	0.0	0	0.0

Number of candidates per <u>phylum</u> per <u>GO biological process</u>

<u>Apicomplexa</u>

unknown	460
pathogenesis	68
cell adhesion	14
glycerol ether metabolic process	5
'de novo' AMP biosynthetic process:'de novo' IMP biosynthetic process	5
protein folding	4
entry into host	4
carbohydrate metabolic process:carboxylic acid metabolic process	4
ubiquitin-dependent protein catabolic process	3
translation	3
signal transduction	3
glutathione metabolic process	3
aromatic amino acid family biosynthetic process: cellular amino acid biosynthetic process	3
respiratory electron transport chain	2
glycolytic process	2
entry into host:pathogenesis	2
dTMP biosynthetic process:methylation:one-carbon metabolic process: tetrahydrofolate biosynthetic process	2
cytoplasmic translational elongation	2
cytoadherence to microvasculature, mediated by symbiont protein	2
actin filament bundle assembly	2
actin cytoskeleton organization: cytoplasmic actin-based contraction involved in cell motility	2
DNA repair	2
tetrahydrobiopterin biosynthetic process	1
regulation of immune response	1
proteolysis	1
protein refolding	1
protein import into nucleus	1
protein folding:response to endoplasmic reticulum stress	1
proteasome-mediated ubiquitin-dependent protein catabolic process	1
nucleotide-excision repair: transcription initiation from RNA polymerase II promoter	1
nucleoside metabolic process	1
heme biosynthetic process	1
gluconeogenesis:glycolytic process	1
exit from host cell:proteolysis:proteolysis involved in cellular protein catabolic process: regulation of immune response	1
evasion of host immune response:modulation by symbiont of host cellular process: suppression by symbiont of host innate immune response	1
cellular response to unfolded protein:chaperone cofactor-dependent protein refolding: response to heat	1
cellular response to unfolded protein:chaperone cofactor-dependent protein refolding	1
cellular amino acid metabolic process	1
cell redox homeostasis	1
cell adhesion:pathogenesis	1
carbohydrate transport	1
calcium-mediated signaling	1
bioluminescence:generation of precursor metabolites and energy	1
attachment of GPI anchor to protein	1

actin filament depolymerization	1
L-serine biosynthetic process	1
**Nematoda**	
unknown	41
epidermal growth factor receptor signaling pathway	2
translation	1
response to heat	1
regulation of transcription, DNA-templated	1
protein refolding	1
locomotion:positive regulation of ovulation:positive regulation of sarcomere organization: skeletal muscle myosin thick filament assembly	1
glutathione metabolic process	1
defecation:endoplasmic reticulum unfolded protein response:hemidesmosome assembly: multicellular organismal reproductive process: negative regulation of oxidative stress-induced neuron death: programmed cell death:protein folding:regulation of gene expression:response to ethanol:response to heat	1
**Platyhelminthes**	
unknown	230
microtubule-based process	46
glutathione metabolic process	15
glycerol ether metabolic process	12
response to oxidative stress	7
biosynthetic process:cellular amino acid metabolic process	7
CTP biosynthetic process:GTP biosynthetic process:UTP biosynthetic process	7
ubiquitin-dependent protein catabolic process	4
gluconeogenesis:glycolytic process	4
glycolytic process	3
cellular amino acid metabolic process	3
regulation of catalytic activity	2
vesicle-mediated transport	1
vacuolar proton-transporting V-type ATPase complex assembly	1
transmembrane transport	1
small GTPase mediated signal transduction	1
retrograde vesicle-mediated transport, Golgi to endoplasmic reticulum	1
proteolysis involved in cellular protein catabolic process	1
proteasome-mediated ubiquitin-dependent protein catabolic process	1
nucleobase-containing compound metabolic process	1
glucose metabolic process:glycolytic process	1
cell redox homeostasis	1
calcium-mediated signaling	1
biosynthetic process	1
aromatic amino acid family metabolic process:dopamine biosynthetic process: neurotransmitter biosynthetic process	1
G protein-coupled receptor signaling pathway:response to pheromone	1
'de novo' AMP biosynthetic process:'de novo' IMP biosynthetic process	1
**Euglenozoa**	
unknown	50
protein folding	4
cell adhesion	3
protein refolding	1
protein initiator methionine removal	1
microtubule-based process	1
methylation:steroid biosynthetic process	1
metabolic process	1
glutathione metabolic process	1

	gluconeogenesis:glycolytic process	1
	evasion of host immune response	1
	defense response:positive regulation of cell population proliferation	1
	cellular lipid metabolic process	1
	cell redox homeostasis	1
	biosynthetic process:cellular amino acid metabolic process	1
**Arthropoda**		
	glutathione metabolic process	2
	protein folding	1
	unknown	1

Apicomplexan candidates per GO biological process

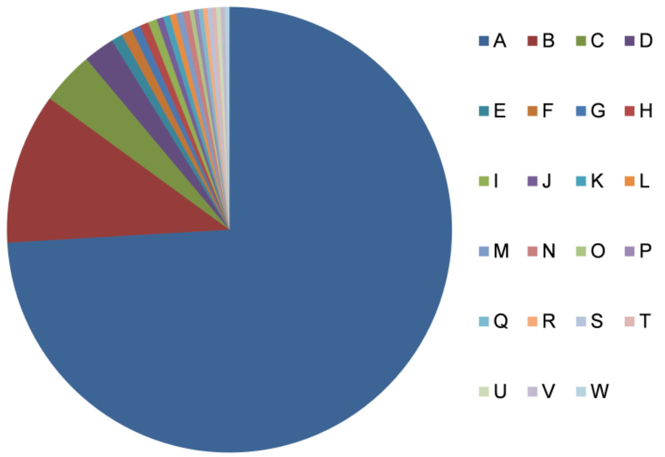

A	460	unknown
B	68	pathogenesis
C	24	other
D	14	cell adhesion
E	5	glycerol ether metabolic process
F	5	'de novo' AMP biosynthetic process:'de novo' IMP biosynthetic process
G	4	protein folding
H	4	entry into host
I	4	carbohydrate metabolic process:carboxylic acid metabolic process
J	3	ubiquitin-dependent protein catabolic process
K	3	translation
L	3	signal transduction
M	3	glutathione metabolic process
N	3	aromatic amino acid family biosynthetic process: cellular amino acid biosynthetic process
O	2	respiratory electron transport chain
P	2	glycolytic process
Q	2	entry into host:pathogenesis
R	2	dTMP biosynthetic process:methylation:one-carbon metabolic process: tetrahydrofolate biosynthetic process
S	2	cytoplasmic translational elongation
T	2	cytoadherence to microvasculature, mediated by symbiont protein
U	2	actin filament bundle assembly
V	2	actin cytoskeleton organization: cytoplasmic actin-based contraction involved in cell motility
W	2	DNA repair

Platyhelminthes candidates per GO biological process

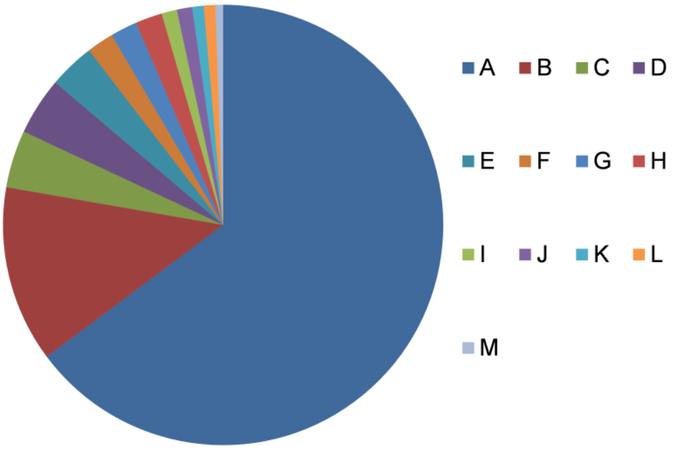

A	230	unknown
B	46	microtubule-based process
C	15	glutathione metabolic process
D	15	other
E	12	glycerol ether metabolic process
F	7	response to oxidative stress
G	7	biosynthetic process:cellular amino acid metabolic process
H	7	CTP biosynthetic process:GTP biosynthetic process:UTP biosynthetic process
I	4	ubiquitin-dependent protein catabolic process
J	4	gluconeogenesis:glycolytic process
K	3	glycolytic process
L	3	cellular amino acid metabolic process
M	2	regulation of catalytic activity

Number of candidates per phylum per GO cellular component

Apicomplexa

unknown	294
integral component of membrane	141
cell surface:integral component of membrane	60
integral component of membrane:plasma membrane	21
membrane	17
anchored component of membrane:plasma membrane	15
cell surface	9
cytoplasm:nucleus:proteasome core complex, alpha-subunit complex	4
cell surface:membrane	4
endoplasmic reticulum lumen	3
cytoplasm:nucleus	3
Ino80 complex:NuA4 histone acetyltransferase complex:R2TP complex	3
integral component of membrane:mitochondrial inner membrane:respirasome	2
extracellular region	2
endoplasmic reticulum	2
cytosolic large ribosomal subunit	2

anchored component of membrane:cell surface:plasma membrane	2
actin cytoskeleton:cytoplasm	2
transcription factor TFIIH core complex	1
small ribosomal subunit	1
ribosome	1
rhoptry neck:symbiont-containing vacuole	1
rhoptry neck	1
proteasome complex	1
phosphopyruvate hydratase complex	1
nucleus	1
mitochondrion	1
mitochondrial inner membrane	1
membrane:symbiont-containing vacuole membrane	1
integral component of membrane:symbiont-containing vacuole membrane:transport vesicle	1
integral component of membrane:symbiont-containing vacuole membrane	1
integral component of membrane:rhoptry neck	1
host cell plasma membrane:merozoite dense granule:symbiont-containing vacuole	1
host cell cytoplasm	1
extracellular space:lysosome:plasma membrane:symbiont-containing vacuolar space:symbiont-containing vacuole	1
extracellular region:integral component of membrane	1
endoplasmic reticulum:integral component of membrane	1
cytoplasmic vesicle:extracellular region:integral component of membrane:microneme membrane	1
cytoplasmic vesicle:extracellular region:host cell membrane:host cell plasma membrane:host extracellular space:microneme:microneme lumen:protein-containing complex	1
cytoplasm:nucleus:ribosome	1
cytoplasm:mitochondrion:PTEX complex:symbiont-containing vacuole	1
cytoplasm:mitochondrion	1
cytoplasm:host cell endoplasmic reticulum:integral component of membrane:symbiont-containing vacuole membrane	1
cytoplasm:extracellular region:host cell plasma membrane:integral component of membrane	1
cell surface:membrane:symbiont-containing vacuole	1
apical part of cell:bicellular tight junction:cytoplasmic vesicle:extracellular region:host cell membrane:host cell plasma membrane:host extracellular space:protein-containing complex:rhoptry:rhoptry lumen	1
anchored component of plasma membrane:rhoptry	1
anchored component of plasma membrane:integral component of membrane:rhoptry neck	1
anchored component of plasma membrane:cell surface	1
anchored component of plasma membrane:apical part of cell:cytoplasmic vesicle:extracellular region:host cell plasma membrane:host extracellular space:microneme:microneme lumen:protein-containing complex	1
anchored component of plasma membrane:apical part of cell:cell surface:symbiont-containing vacuolar space	1
actin cytoskeleton	1

	GPI-anchor transamidase complex	1
**Nematoda**		
	unknown	31
	integral component of membrane	5
	nucleus	2
	cytoskeleton	2
	collagen trimer	2
	troponin complex	1
	myofibril:myosin complex:myosin filament	1
	extracellular space	1
	endoplasmic reticulum lumen	1
	cytoskeleton:integral component of membrane:ribosome	1
	cytoplasm:myosin complex:myosin filament	1
	collagen trimer:integral component of membrane	1
	A band:myosin complex:myosin filament	1
**Platyhelminthes**		
	unknown	189
	integral component of membrane	92
	cytoplasm:dynein complex:microtubule	27
	dynein complex	19
	cytoplasm:nucleus:proteasome core complex, alpha-subunit complex	5
	cytoplasm	5
	myofibril:myosin complex:myosin filament	3
	myofibril:myosin complex	3
	nucleus	2
	extracellular region	2
	phosphopyruvate hydratase complex	1
	perinuclear region of cytoplasm	1
	lysosome	1
	integral component of membrane:plasma membrane	1
	integral component of membrane:nuclear inner membrane	1
	extracellular space	1
	endoplasmic reticulum membrane:integral component of membrane	1
	endoplasmic reticulum lumen	1
**Euglenozoa**		
	unknown	47
	integral component of membrane	4
	nucleosome:nucleus	3
	plasma membrane	2
	integral component of membrane:plasma membrane	2
	endoplasmic reticulum lumen	2
	cytoplasm	2
	ribosome	1
	mitochondrial inner membrane	1
	microtubule	1
	integral component of membrane:mitochondrial inner membrane	1

	endoplasmic reticulum membrane:integral component of membrane	1
	cytoplasm:microtubule	1
	anchored component of membrane:plasma membrane	1
**Arthropoda**		
	unknown	2
	myosin complex	1
	endoplasmic reticulum lumen	1

<u>Apicomplexan</u> candidates per <u>GO cellular component</u>

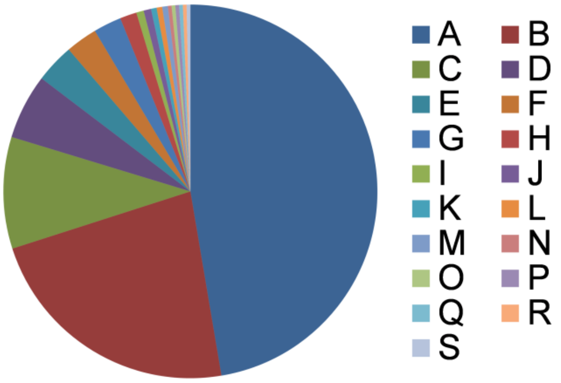

A	294	unknown
B	141	integral component of membrane
C	60	cell surface:integral component of membrane
D	35	other
E	21	integral component of membrane:plasma membrane
F	17	membrane
G	15	anchored component of membrane:plasma membrane
H	9	cell surface
I	4	cytoplasm:nucleus:proteasome core complex, alpha-subunit complex
J	4	cell surface:membrane
K	3	endoplasmic reticulum lumen
L	3	cytoplasm:nucleus
M	3	Ino80 complex:NuA4 histone acetyltransferase complex:R2TP complex
N	2	integral component of membrane:mitochondrial inner membrane:respirasome
O	2	extracellular region
P	2	endoplasmic reticulum
Q	2	cytosolic large ribosomal subunit
R	2	anchored component of membrane:cell surface:plasma membrane
S	2	actin cytoskeleton:cytoplasm

Platyhelminthes candidates per GO cellular component

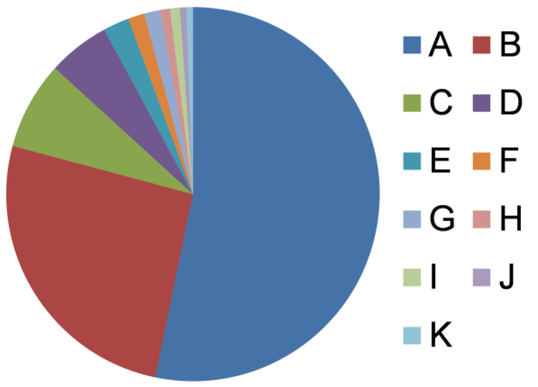

A	189	unknown
B	92	integral component of membrane
C	27	cytoplasm:dynein complex:microtubule
D	19	dynein complex
E	8	other
F	5	cytoplasm:nucleus:proteasome core complex, alpha-subunit complex
G	5	cytoplasm
H	3	myofibril:myosin complex:myosin filament
I	3	myofibril:myosin complex
J	2	nucleus
K	2	extracellular region

Number of candidates per phylum per GO molecular function

Apicomplexa

unknown	414
host cell surface receptor binding	29
serine-type endopeptidase activity	26
ATP binding:protein kinase activity	13
ATP binding:MAP kinase activity:protein serine kinase activity:protein threonine kinase activity	11
kinase activity	6
ATPase activity:ATP binding	6
protein disulfide oxidoreductase activity	5
(S)-2-(5-amino-1-(5-phospho-D-ribosyl)imidazole-4-carboxamido)succinate AMP-lyase (fumarate-forming) activity:N6-(1,2-dicarboxyethyl)AMP AMP-lyase (fumarate-forming) activity	5
oxidoreductase activity, acting on the CH-OH group of donors, NAD or NADP as acceptor	4
hydrolase activity	4
calcium ion binding	4
structural constituent of ribosome	3
protein disulfide isomerase activity	3
metal ion binding:superoxide dismutase activity	3
cysteine-type peptidase activity	3
chorismate synthase activity	3

ATP binding:myosin light chain kinase activity	3
ATP binding:calcium ion binding:calmodulin-dependent protein kinase activity	3
5'-3' DNA helicase activity:ATP binding:hydrolase activity	3
transferase activity, transferring acyl groups, acyl groups converted into alkyl on transfer	2
metal ion binding:NAD-dependent histone deacetylase activity (H3-K14 specific)	2
glutathione transferase activity	2
electron transfer activity:metal ion binding	2
dihydrofolate reductase activity:thymidylate synthase activity	2
calcium ion binding:serine-type endopeptidase activity	2
aspartic-type endopeptidase activity	2
actin monomer binding:phospholipid binding	2
actin filament binding	2
actin binding	2
ATPase-coupled transmembrane transporter activity:ATP binding	2
ATPase activity:ATP binding:unfolded protein binding	2
ATPase activity:ATP binding:heat shock protein binding:misfolded protein binding:protein folding chaperone:unfolded protein binding	2
ATP binding:kinase activity:magnesium ion binding:potassium ion binding:pyruvate kinase activity	2
ATP binding:calmodulin-dependent protein kinase activity	2
ATP binding:calcium ion binding:protein kinase activity	2
zinc ion binding	1
uridine phosphorylase activity	1
transmembrane transporter activity	1
transferase activity	1
threonine-type endopeptidase activity	1
serine-type endopeptidase activity:structural constituent of ribosome	1
protein-disulfide reductase activity	1
phosphoprotein phosphatase activity	1
phosphoglycerate mutase activity	1
peroxidase activity:peroxiredoxin activity	1
peptidase activity	1
oxidoreductase activity, acting on a sulfur group of donors, disulfide as acceptor:protein disulfide oxidoreductase activity:protein-disulfide reductase activity	1
nucleotide binding:oxidoreductase activity, acting on the CH-NH2 group of donors, NAD or NADP as acceptor	1
metal ion binding	1
magnesium ion binding:phosphopyruvate hydratase activity	1
hydrolase activity:zinc ion binding	1
host cell surface receptor binding:metal ion binding	1
host cell surface binding:host cell surface receptor binding	1
heparin binding:host cell surface receptor binding	1
glucose-6-phosphate isomerase activity	1
ferrochelatase activity	1

	electron transfer activity:protein disulfide isomerase activity	1
	cysteine-type endopeptidase activity:cysteine-type peptidase activity:kinase binding:serine-type peptidase activity	1
	cysteine-type endopeptidase activity	1
	copper ion transmembrane transporter activity	1
	calcium ion binding:transferase activity, transferring acyl groups	1
	calcium ion binding:kinase activity	1
	aspartic-type endopeptidase activity:serine-type endopeptidase activity	1
	arginase activity:metal ion binding	1
	RNA binding:structural constituent of ribosome	1
	NAD+ binding:transferase activity	1
	NAD binding:phosphoglycerate dehydrogenase activity	1
	ATPase activity:ATP binding:hydrolase activity:unfolded protein binding	1
	ATPase activity:ATP binding:hydrolase activity	1
	ATP binding:protein serine/threonine kinase activity	1
	ATP binding:nucleosome-dependent ATPase activity	1
	ATP binding:calcium ion binding:calmodulin-dependent protein kinase activity:starch binding	1
	4-alpha-hydroxytetrahydrobiopterin dehydratase activity	1
**Nematoda**		
	unknown	27
	peroxidase activity:peroxiredoxin activity	3
	metal ion binding:superoxide dismutase activity	3
	serine-type endopeptidase activity	2
	epidermal growth factor receptor binding:receptor ligand activity	2
	deoxyribonuclease II activity	2
	ATPase activity:ATP binding	2
	structural constituent of ribosome	1
	structural constituent of cuticle	1
	serine-type endopeptidase inhibitor activity	1
	glutathione transferase activity	1
	cytoskeletal protein binding:protein kinase binding:SH3 domain binding	1
	cysteine-type peptidase activity	1
	calcium ion binding:unfolded protein binding	1
	aminopeptidase activity:metallopeptidase activity:zinc ion binding	1
	DNA-binding transcription factor activity:sequence-specific DNA binding:zinc ion binding	1
**Platyhelminthes**		
	unknown	165
	calcium ion binding	37
	calcium-dependent phospholipid binding:calcium ion binding	20
	cysteine-type peptidase activity	16
	glutathione transferase activity	14
	protein disulfide oxidoreductase activity	10
	serine-type endopeptidase inhibitor activity	7
	glutathione peroxidase activity	7
	L-aspartate:2-oxoglutarate aminotransferase activity:pyridoxal phosphate	7

binding	
nucleoside diphosphate kinase activity	6
cysteine-type endopeptidase activity	4
alkaline phosphatase activity	4
ATP binding:kinase activity:magnesium ion binding:potassium ion binding:pyruvate kinase activity	4
triose-phosphate isomerase activity	3
metal ion binding:superoxide dismutase activity	3
hydrolase activity	3
glutamate dehydrogenase (NAD+) activity:glutamate dehydrogenase (NADP+) activity:nucleotide binding	3
nuclear receptor activity:sequence-specific DNA binding:zinc ion binding	2
manganese ion binding:metalloaminopeptidase activity	2
actin binding	2
ATPase activity:ATP binding	2
ATP binding:phosphoglycerate kinase activity	2
toxic substance binding	1
threonine-type endopeptidase activity	1
serine-type endopeptidase activity	1
ribonuclease T2 activity:RNA binding	1
pyridoxal phosphate binding:transaminase activity	1
oxidoreductase activity, acting on a sulfur group of donors, NAD(P) as acceptor	1
metal ion binding:protein disulfide oxidoreductase activity	1
magnesium ion binding:phosphopyruvate hydratase activity	1
lipid binding	1
isomerase activity:protein disulfide oxidoreductase activity	1
iron ion binding:tyrosine 3-monooxygenase activity	1
identical protein binding:triose-phosphate isomerase activity	1
identical protein binding:peroxidase activity:peroxiredoxin activity	1
growth factor activity	1
glyceraldehyde-3-phosphate dehydrogenase (NAD+) (phosphorylating) activity:NAD binding:NADP binding	1
glutathione transferase activity:translation elongation factor activity	1
glutathione transferase activity:isomerase activity	1
flavin-linked sulfhydryl oxidase activity	1
flavin adenine dinucleotide binding:protein disulfide oxidoreductase activity:thioredoxin-disulfide reductase activity	1
electron transfer activity:glutathione transferase activity	1
cysteine-type endopeptidase inhibitor activity	1
chitin binding:serine-type endopeptidase inhibitor activity	1
calcium-dependent phospholipid binding:calcium ion binding:phospholipase inhibitor activity	1
calcium-dependent phospholipid binding:calcium ion binding:identical protein binding:phospholipase inhibitor activity	1
calcium-dependent cysteine-type endopeptidase activity:calcium ion binding	1
aminopeptidase activity	1
adenylate kinase activity:ATP binding	1

	NAD binding:oxidoreductase activity, acting on the aldehyde or oxo group of donors, NAD or NADP as acceptor	1
	GTPase activity:GTP binding	1
	ATP binding:nucleoside diphosphate kinase activity	1
	ATP binding:nucleobase-containing compound kinase activity	1
	(S)-2-(5-amino-1-(5-phospho-D-ribosyl)imidazole-4-carboxamido)succinate AMP-lyase (fumarate-forming) activity:N6-(1,2-dicarboxyethyl)AMP AMP-lyase (fumarate-forming) activity	1
**Euglenozoa**		
	unknown	20
	ATP binding:MAP kinase activity:protein serine kinase activity:protein threonine kinase activity	11
	metal ion binding:superoxide dismutase activity	6
	metal ion binding:metalloendopeptidase activity	3
	cysteine-type peptidase activity	3
	DNA binding:protein heterodimerization activity	3
	ATPase activity:ATP binding:unfolded protein binding	3
	ATPase activity:ATP binding	3
	protein disulfide isomerase activity	2
	triose-phosphate isomerase activity	1
	purine nucleosidase activity	1
	peroxidase activity:peroxiredoxin activity	1
	methyltransferase activity	1
	metal ion binding:transketolase activity	1
	metal ion binding:metalloaminopeptidase activity	1
	kinase activity	1
	glycerone-phosphate O-acyltransferase activity	1
	flavin adenine dinucleotide binding:trypanothione-disulfide reductase activity	1
	electron transfer activity:identical protein binding	1
	cysteine-type endopeptidase activity	1
	calcium ion binding:unfolded protein binding	1
	calcium ion binding	1
	L-aspartate:2-oxoglutarate aminotransferase activity:pyridoxal phosphate binding	1
	GTPase activity:GTP binding:structural constituent of cytoskeleton	1
**Arthropoda**		
	transferase activity	1
	unknown	1
	glutathione transferase activity	1
	calcium ion binding:unfolded protein binding	1

Apicomplexan candidates per GO molecular function

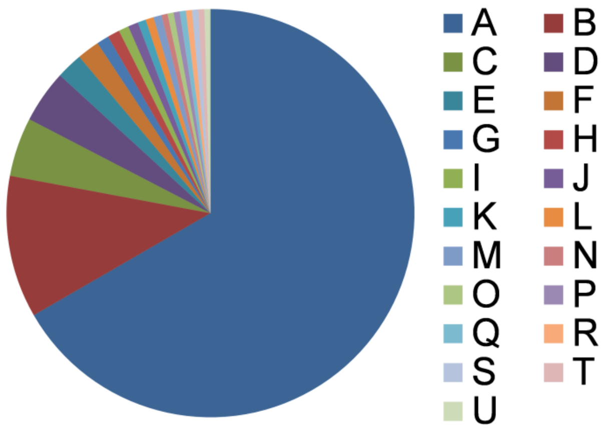

A	414	unknown
B	70	other
C	29	host cell surface receptor binding
D	26	serine-type endopeptidase activity
E	13	ATP binding:protein kinase activity
F	11	ATP binding:MAP kinase activity:protein serine kinase activity: protein threonine kinase activity
G	6	kinase activity
H	6	ATPase activity:ATP binding
I	5	protein disulfide oxidoreductase activity
J	5	(S)-2-(5-amino-1-(5-phospho-D-ribosyl)imidazole-4-carboxamido)succinate AMP-lyase (fumarate-forming) activity:N6-(1,2-dicarboxyethyl)AMP AMP-lyase (fumarate-forming) activity
K	4	oxidoreductase activity, acting on the CH-OH group of donors, NAD or NADP as acceptor
L	4	hydrolase activity
M	4	calcium ion binding
N	3	structural constituent of ribosome
O	3	protein disulfide isomerase activity
P	3	metal ion binding:superoxide dismutase activity
Q	3	cysteine-type peptidase activity
R	3	chorismate synthase activity
S	3	ATP binding:myosin light chain kinase activity
T	3	ATP binding:calcium ion binding:calmodulin-dependent protein kinase activity
U	3	5'-3' DNA helicase activity:ATP binding:hydrolase activity

<u>Platyhelminthes</u> candidates per <u>GO molecular function</u>

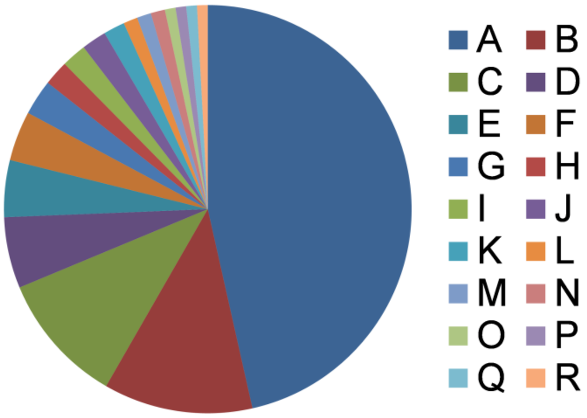

A	165	unknown
B	42	other
C	37	calcium ion binding
D	20	calcium-dependent phospholipid binding:calcium ion binding
E	16	cysteine-type peptidase activity
F	14	glutathione transferase activity
G	10	protein disulfide oxidoreductase activity
H	7	serine-type endopeptidase inhibitor activity
I	7	glutathione peroxidase activity
J	7	L-aspartate:2-oxoglutarate aminotransferase activity:pyridoxal phosphate binding
K	6	nucleoside diphosphate kinase activity
L	4	cysteine-type endopeptidase activity
M	4	alkaline phosphatase activity
N	4	ATP binding:kinase activity:magnesium ion binding:potassium ion binding:pyruvate kinase activity
O	3	triose-phosphate isomerase activity
P	3	metal ion binding:superoxide dismutase activity
Q	3	hydrolase activity
R	3	glutamate dehydrogenase (NAD+) activity:glutamate dehydrogenase (NADP+) activity:nucleotide binding
